# Phosphorylation of 17β-hydroxysteroid dehydrogenase 13 at serine 33 attenuates nonalcoholic fatty liver disease in mice

**DOI:** 10.1038/s41467-022-34299-1

**Published:** 2022-11-02

**Authors:** Wen Su, Sijin Wu, Yongliang Yang, Yanlin Guo, Haibo Zhang, Jie Su, Lei Chen, Zhuo Mao, Rongfeng Lan, Rong Cao, Chunjiong Wang, Hu Xu, Cong Zhang, Sha Li, Min Gao, Xiaocong Chen, Zhiyou Zheng, Bing Wang, Yi’ao Liu, Zuojun Liu, Zimei Wang, Baohua Liu, Xinmin Fan, Xiaoyan Zhang, Youfei Guan

**Affiliations:** 1 Department of Pathophysiology, Shenzhen University, Shenzhen, 518060 China; 2 Shenzhen University Health Science Center, Shenzhen University, Shenzhen, 518060 China; 3 State Key Laboratory of Molecular Reaction Dynamics, Dalian Institute of Chemical Physics, Chinese Academy of Sciences, Dalian, 116024 China; 4 Laboratoy of Innovative Drug Discovery, School of Bioengineering, Dalian University of Technology, Dalian, 116023 China; 5 Health Science Center, East China Normal University, Shanghai, 200241 China; 6 Advanced Institute for Medical Sciences, Dalian Medical University, Dalian, 116044 China; 7 Department of Nephrology, The First Affiliated Hospital of Shenzhen University, Shenzhen, 518035 China; 8 Department of Physiology and Pathophysiology, The Province and Ministry Co-sponsored Collaborative Innovation Center for Medical Epigenetics, Tianjin Medical University, Tianjin, China; 9 Medical College, Hebei University of Engineering, Handan, China; 10 Department of Physiology and Pathophysiology, School of Basic Medical Sciences, Dalian Medical University, Dalian, 116044 China

**Keywords:** Fat metabolism, Non-alcoholic fatty liver disease, Metabolic diseases

## Abstract

17β-hydroxysteroid dehydrogenase-13 is a hepatocyte-specific, lipid droplet-associated protein. A common loss-of-function variant of *HSD17B13* (rs72613567: TA) protects patients against non-alcoholic fatty liver disease with underlying mechanism incompletely understood. In the present study, we identify the serine 33 of 17β-HSD13 as an evolutionally conserved PKA target site and its phosphorylation facilitates lipolysis by promoting its interaction with ATGL on lipid droplets. Targeted mutation of Ser33 to Ala (S33A) decreases ATGL-dependent lipolysis in cultured hepatocytes by reducing CGI-58-mediated ATGL activation. Importantly, a transgenic knock-in mouse strain carrying the *HSD17B13* S33A mutation (*HSD17B13*^*33A/A*^) spontaneously develops hepatic steatosis with reduced lipolysis and increased inflammation. Moreover, *Hsd17B13*^*33A/A*^ mice are more susceptible to high-fat diet-induced nonalcoholic steatohepatitis. Finally, we find reproterol, a potential 17β-HSD13 modulator and FDA-approved drug, confers a protection against nonalcoholic steatohepatitis via PKA-mediated Ser33 phosphorylation of 17β-HSD13. Therefore, targeting the Ser33 phosphorylation site could represent a potential approach to treat NASH.

## Abstract (web summary)

A truncating variant of 17β-hydroxysteroid dehydrogenase-13 (17β-HSD13), a lipid droplet -associated protein, associates with lower risk of chronic liver disease. Here the authors identify a phosphorylation site in 17β-HSD13 which promotes lipolysis, and report that a knock-in mouse with a 17β-HSD13 mutant defective for phosphorylation is more susceptible to nonalcoholic steatohepatitis.

## Introduction

NAFLD is strongly associated with the development of obesity, insulin resistance, type 2 diabetes and cardiovascular diseases and represents a leading risk factor for many end-stage liver diseases. The prevalence of NAFLD is rapidly increasing, with a global prevalence more than 25%^1^. Non-alcoholic steatohepatitis (NASH) is an advanced stage of NAFLD, with an prevalence of approximately 25–30% in NAFLD, and is characterized by steatosis, inflammation, hepatocyte injury (swelling), and fibrosis, which may eventually lead to cirrhosis and hepatocellular carcinoma (HCC)^2^. It is predicted that NAFLD/NASH would become a major indication for liver transplantation in the United States and many developed countries^3^. However, there are no established therapies or FDA-approved drugs for NAFLD/NASH. The pathogenesis and the underlying mechanisms of NAFLD/NASH remain largely unknown. Therefore, identifying new pharmacological approaches to effectively treat NAFLD/NASH is essential in clinical practice.

The storage and mobilization of triacylglycerol (TAG) in the liver are critical processes for energy homeostasis throughout the body. Adipose triglyceride lipase (ATGL), a member of the Patatin lipase family, is the rate-limiting lipolytic enzyme in TAG hydrolysis in adipose tissue, muscle, and liver^4^. The dynamic interaction between ATGL and its co-activators is regulated in part by tissue-specific perilipins, which functions as scaffolds to orchestrate this interaction at the surface of lipid droplets (LDs) for fatty acid efflux and oxidation^5^. In adipocytes, perilipin 1 (PLIN1) and PLIN5 sequester comparative gene identification 58 (CGI-58, also known as ABHD5), a co-activator of ATGL, in the basal state. Extracellular signals leading to PLIN1 phosphorylation release CGI-58 to activate ATGL. However, PLIN1 is barely detectable in the liver, suggesting that there are other proteins in hepatocytes responsible for regulating hepatic TAG metabolism. It has been recently reported that Patatin-like phospholipase domain containing 3 (PNPLA3), specifically its I148M variant (*PNPLA3*-rs738409), is an important lipid droplet (LD)-associated protein that dynamically interacts with CGI-58, leading to the inhibition of ATGL catabolism in the liver^6^.

In addition to *PNPLA3*-rs738409, many other genetic variants have been found to be associated with increased risk of NAFLD, including transmembrane 6 superfamily member 2 (*TM6SF2*)-rs58542926 and 17β-hydroxysteroid dehydrogenase-13 (*HSD17B13*)-rs72613567. *HSD17B13* is a newly identified gene encoding a liver-enriched and hepatocyte-specific LD protein that enhances hepatic lipogenesis and promotes the pathogenesis of NAFLD^7^. In the recent years, *HSD17B13* has attracted widespread attention as a genetic risk factor for NAFLD and an useful biomarker for chronic liver diseases in human^8^. In a genome-wide association study (GWAS) of 46,544 Americans by Abul-Husn et al., association between a loss of function variant of *HSD17B13* (rs72613567: TA insertion, allele frequency 26%) and decreased ALT levels and reduced risk of various chronic liver diseases was identified^9^. Subsequently, the hepatoprotective role of the *HSD17B13* rs72613567 variant was confirmed in many independent population-based genetic studies of chronic liver diseases^5–9^. However, to date, the biological role and molecular mechanism of *HSD17B13* in the regulation of hepatic lipid metabolism and the pathogenesis of NAFLD/NASH remain incompletely characterized and poorly understood.

In the present study, we report a critical role of the PKA-induced phosphorylation of 17β-HSD13 at serine 33 (S33), an evolutionally conserved residue, in the regulation of hepatic lipid homeostasis and in the pathogenesis of NAFLD/NASH. We identified 17β-HSD13 as a ATGL binding protein that recruits ATGL on the surface of LDs. In addition, 17β-HSD13 competed for the interaction of CGI-58 with ATGL, thereby inhibiting ATGL-dependent lipolysis in hepatocytes. A mutant defective for the PKA phosphorylation site (17β-HSD13 S33A) further reduced ATGL-dependent lipolysis by sequestering CGI-58, representing a gain-of-function in ATGL binding and lipolysis inhibition. Moreover, the 17β-HSD13 S33A mutation knock-in mice spontaneously developed NAFLD. Furthermore, reproterol, a potential 17β-HSD13 modulator and FDA-approved drug, was found to greatly attenuate high-fat diet (HFD)-induced NAFLD/NASH by enhancing 17β-HSD13 phosphorylation at Ser33, leading to increased ATGL activity. Collectively, our findings reveal the mechanism by which dephosphorylated 17β-HSD13 promotes hepatic lipid accumulation and provide evidence that targeting 17β-HSD13 phosphorylation at Ser33 may represent a therapeutic strategy for the treatment of NAFLD and NASH.

## Results

### PKA phosphorylates 17β-HSD13 at serine 33

**Fig. 1:**
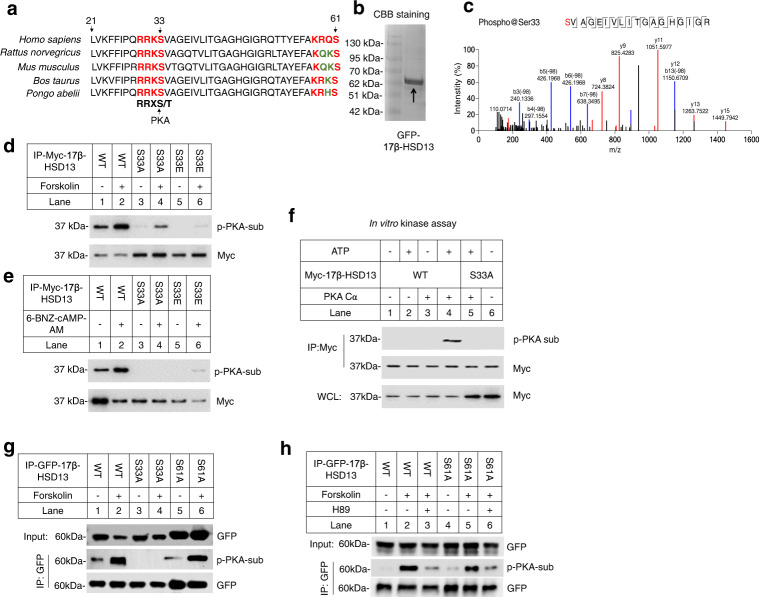
PKA phosphorylates 17β-HSD13 at serine 33 residue. **a** The N terminal amino acid sequences of 17β-HSD13 from *homo sapiens*, *rattus*, *mus*, *bos taurus* and *pongo abelii* are aligned. A conserved serine 33 residue (Ser33) which is predicted to be a potential PKA phosphorylation site was identified among species. **b** HEK293 cells were transfected with a GFP-tagged 17β-HSD13 expression vector for 24 h, then treated with 10 μM forskolin for 30 min, followed by immunoprecipitation (IP) with anti-GFP-beads. The IP product was loaded and stained with Coomassie brilliant blue (CBB). **c** The major band in **b** was cut and digested. Phosphor-peptides were detected by LC-MS/MS. Only the serine 33 was found to be phosphorylated. Data were reproduced in three independent experiments. **d**, **e** HEK293 cells were transfected with an expression vector carrying a full-length myc-tagged 17β-HSD13 wild-type (WT), S33A mutant or S33E mutant, followed by IP with an anti-myc antibody. 10 μM forskolin (**d**) or 10 μM 6-BNZ-cAMP-AM (**e**) was added 30 min before lysis, respectively. Proteins retained on sepharose were blotted with an anti-phospho-(Ser/Thr) PKA substrate antibody. **f** HEK293 cells were transfected with a full-length myc-tagged 17β-HSD13 WT or S33A mutant, followed by IP with an anti-myc antibody. Proteins retained on sepharose were incubated with an active recombinant PKA Cα subunit for the kinase assay and then blotted with an anti-phospho-(Ser/Thr) PKA substrate antibody or anti-myc antibody. **g**, **h** HEK293 cells were transfected with a full-length GFP-tagged 17β-HSD13 WT, S33A or S61A mutant for 24 h and then pretreated with forskolin for 30 min with or without H89 for 1 h as indicated, followed by IP with an anti-GFP antibody. Proteins retained on sepharose were then blotted with an anti-phospho-(Ser/Thr) PKA substrate (p-PKA-sub). *n* = 2 biologically independent experiments for **b–h**.

We first analyzed the amino acid sequence of 17β-HSD13 by the NetPhos 3.1. Sequence analysis of 17β-HSD13 highlighted two potential phosphorylation sites including Ser33 and Ser61. Of these two sites, the Ser33 scored relatively high at 0.884 and the RRKS site is highly conserved across species (Fig. 1a). PKA phosphorylates the substrates with the motif R/K-R/K-X-S/T on their surface^10^. The nano LC MS/MS technique successfully identified two peptides with phosphorylated Ser33 of 17β-HSD13 protein extracted from forskolin-treated 293T cells expressing a GFP-tagged wild-type (WT) 17β-HSD13 vector **(**Fig. 1b, c and Fig. S1). Hence, we first focused on investigating the function of the Ser33 residue. Forskolin is a widely used activator of adenylyl cyclase, and potently activates PKA. We determined whether forskolin induces 17β-HSD13 phosphorylation. HEK293 cells were transfected with either a myc-tagged full-length wild-type (WT) or S33 site-mutated (S33A or S33E) 17β-HSD13 for 24 h followed by the treatment with forskolin or 6-BNZ-cAMP-AM for 30 min (Fig. 1d, e). 17β-HSD13 was then immunoprecipitated using an anti-myc antibody and detected with an antibody specific for the phosphor-(Ser/Thr) PKA substrate. The results showed that forskolin markedly induced the phosphorylation of the WT 17β-HSD13 as a substrate of PKA (Fig. 1d, e). In vitro kinase assay using purified myc-tagged WT 17β-HSD13 and PKA Cα (catalytic subunit) proteins further demonstrated that only wild type (WT), but not the S33A mutant, of 17β-HSD13 could be phosphorylated by PKA Cα (Fig. 1f), suggesting that PKA Cα can directly phosphorylate 17β-HSD13 in vitro. In addition, the S33A mutation but not the S61A mutation dramatically abolished 17β-HSD13 phosphorylation not only at baseline but also under the conditions of PKA activation (Fig. 1g). In parallel, the PKA inhibitor H89 could inhibit forskolin-induced phosphorylation of both wild-type (WT) and S61A mutant forms of HSD17B13 (Fig. 1h). Taken together, these data indicate that the Ser33 residue of 17β-HSD13 is the major phosphorylation site for PKA.

### The Ser33 of 17β-HSD13 is critical for hepatic LD metabolism

**Fig. 2:**
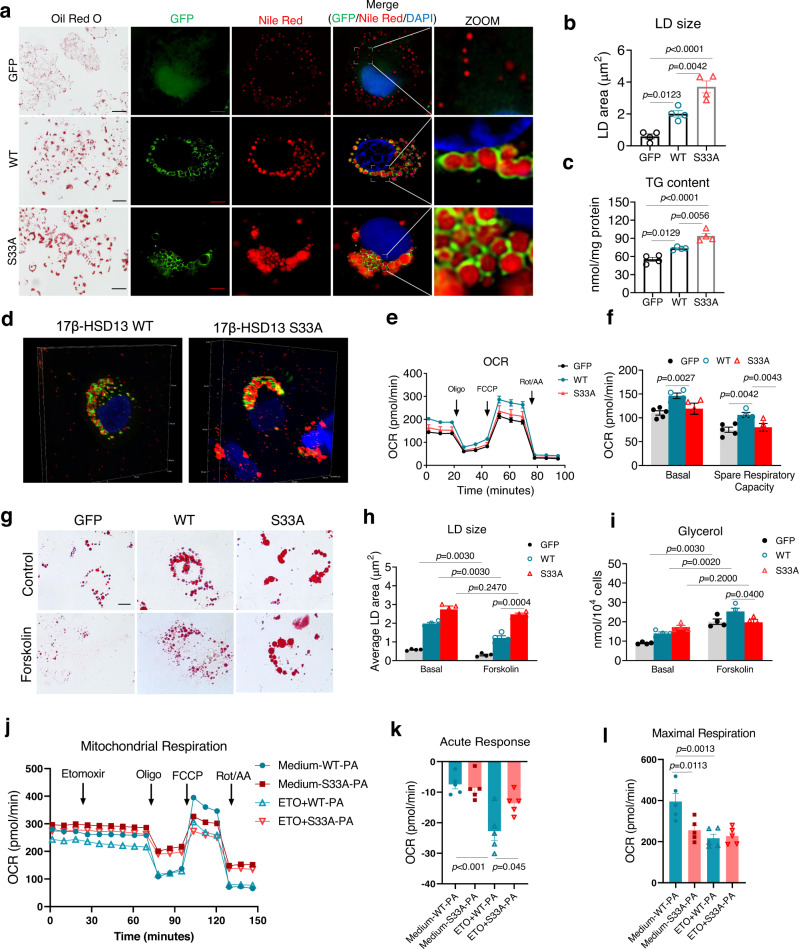
S33A mutant cells exhibit larger lipid droplets with reduced lipolysis and fatty acid oxidation. **a** Huh7 cells were infected with lentiviruses carrying GFP, GFP-tagged 17β-HSD13 WT or GFP-tagged S33A mutant. Oil red O staining (scale bar, 50 μm) and confocal images (scale bar, 5 μm) were analyzed. Lipid droplets were stained by Nile red (red) and the nucleus was stained by DAPI (blue). LD size (**b**) and TG content (**c**) were analyzed. GFP: *n* = 4; WT: *n*  =  4; S33A: *n*  =  4 biologically independent cells. **d** N-SIM-3D reconstruction of the subcellular localization of 17β-HSD13 WT and 17β-HSD13 S33A protein in Huh7 cells. Lipid droplets were stained with Nile red (red) and 17β-HSD13 WT and 17β-HSD13 S33A proteins were visualized by green fluoresce. **e**, **f** Huh7 cells were stably infected with the lentiviruses carrying a full-length GFP (GFP), GFP-tagged 17β-HSD13 WT (WT) or GFP-tagged 17β-HSD13 S33A mutant (S33A). Mitochondrial respiration was analyzed in real-time using the Seahorse XF24 Extracellular Flux Analyzer. The oxygen consumption rate (OCR) (**e**) at different stages of respiration, basal and spare respiratory capacity (**f**) were measured as described in the “Methods” section. Data presented are the mean ± SEM. One-way ANOVA with Bonferroni post hoc analysis was performed. GFP: *n* = 5; WT: *n* = 4; S33A: *n* = 4 biologically independent cells. **g**–**i** Lipolysis was induced by forskolin treatment (10 μM) for 24 h in Huh7 cells with stable expression of GFP, 17β-HSD13 WT (WT) or GFP-tagged 17β-HSD13 S33A mutant (S33A). Oil red O staining was performed (**g**) and the LD sizes (**h**) were analyzed. **i** Glycerol levels in culture medium were measured. GFP: *n* = 5; WT: *n* = 4; S33A: *n* = 4 biologically independent cells. **j**–**l** Oxygen consumption rate (OCR) in the presence of exogenous palmitic acid in WT and the S33A mutant Huh7 cells. Advanced ORC was measured in real-time using the Seahorse® metabolic flux analyzer as described in the section of “Methods”. **j** The OCR curves. The times of addition of etomoxir (ETO), oligomycin (oligo), FCCP, and rotenone + antimycin A (Rot/AA) were indicated at the top. The OCRs in the presence of palmitate-BSA (PA) and ETO were displayed. **k** Acute response due to the addition of ETO under basal condition. **l** Maximal respiration due to the addition of FCCP. Medium-WT-PA: *n* = 5; Medium-S33A-PA: *n* = 4; ETO-WT-PA: *n* = 5; ETO-S33A-PA: *n* = 4 biologically independent cells. Data represent mean ± SEM; Two-tailed student’s t test was performed for **b**, **c**; One-way ANOVA with Bonferroni post hoc analysis was performed for **e**–**l**.

We next thought to explore the effect of the phosphorylation of 17β-HSD13 at Ser33 on lipid metabolism in cultured hepatocytes. A GFP-tagged full-length wild-type (WT) or S33A mutant 17β-HSD13 lentivirus was used to infect Huh7 cells with more than 90% percent infection rate. Under basal conditions, the expression levels of 17β-HSD13 (indicated as GFP) were similar between WT and Ser33A mutant groups as assessed by immunoblot analysis (Fig. S2a). Oil Red O staining revealed that overexpression of the 17β-HSD13 S33A mutant markedly promoted neutral lipid accumulation compared to the WT form (Fig. 2a). In accordance with our previous study, morphological examination by confocal microscopy revealed that 17β-HSD13 was exclusively located on the surface of LDs (Fig. 2a). The average size of LDs was significantly increased in the cells transfected with the 17β-HSD13 S33A mutant compared with the WT form (Fig. 2b). Consistently, TG contents were significantly higher in the 17β-HSD13 S33A mutant-expressing cells than that in 17β-HSD13 WT-expressing cells (Fig. 2c). In addition, 3D reconstruction by using N-SIM clearly showed that the 17β-HSD13 S33A mutant displayed much bigger lipid droplet volume compared with the 17β-HSD13 WT in Huh7 cells (Fig. 2d). These findings indicate that the Ser33 of 17β-HSD13 is critical for lipid droplet formation and lipid metabolism. It is well known that PKA plays a pivotal role in regulating lipolysis via phosphorylation of a variety of LD-associated proteins including PLIN1, PLIN3, PLIN5, ATGL, and HSL. These signaling pathways have been reported to promote triacylglycerol hydrolysis and fatty acid oxidation. To determine the effect of the 17β-HSD13 S33A mutation on mitochondrial respiration, the Huh7 cells with stable expression of the 17β-HSD13 WT and 17β-HSD13 S33A mutant were generated and the mitochondria respiratory capacity was analyzed by the Seahorse XF24 (Fig. 2e). The results showed that while 17β-HSD13 WT cells displayed higher OCR levels compared with the control GFP cells, the S33A mutant-expressing cells showed significantly reduced mitochondria respiratory capacity compared to the 17β-HSD13-WT-expressing cells (Fig. 2e, f). In line with Huh7 data, we observed a similar effect in HepG2 cells (Fig. S2b, c). These findings demonstrate that the 17β-HSD13 S33A mutant-expressing cells have reduced capacity of mitochondrial respiration, possibly due to inhibited LD lipolysis. To further determine the mechanism by which the PKA-mediated S33 phosphorylation of 17β-HSD13 in hepatocyte lipid metabolism, we treated Huh7 cells with forskolin (10 μM) to induce lipolysis (Fig. 2g). Similar to the finding of a previous report^11^, glycerol release was significantly increased and the average size of LDs were gradually decreased after forskolin treatment in 17β-HSD13 WT-transfected cells (Fig. 2h, i). However, the cells expressing the 17β-HSD13 S33A mutant were resistant to forskolin-induced lipolysis and the size of LDs remained unchanged following forskolin treatment (Fig. 2g–i). We next performed a fatty acid-dependent oxygen consumption (OCR) experiment with the combined use of the Palmitate-BSA FAO Substrate and etomoxir (an CPT-1a inhibitor) (Fig. 2j). When exogenous palmitate-BSA was added as energy substrate, the Huh7 cells expressing the S33A-HSD17B13 mutant displayed a comparable basal OCR level (Fig. S2d) but decreased acute response (Fig. 2k), maximal respiration (Fig. 2i) and FA dependent maximal response (Fig. S2e). These findings indicate that the Huh7 cells expressing the S33A-HSD17B13 mutant exhibit reduced capacity of mitochondrial palmitate oxidation compared with WT-HSD17B13 cells, probably due to reduced FA flux from lipid droplet lipolysis. Together, these data demonstrate that the 17β-HSD13 S33A mutant resists forskolin-induced, PKA-dependent phosphorylation, thereby leading to the suppression of lipolysis.

### Targeted knock-in of the 17β-HSD13 S33A mutation augments hepatic LD formation to drive NAFLD pathogenesis

**Fig. 3:**
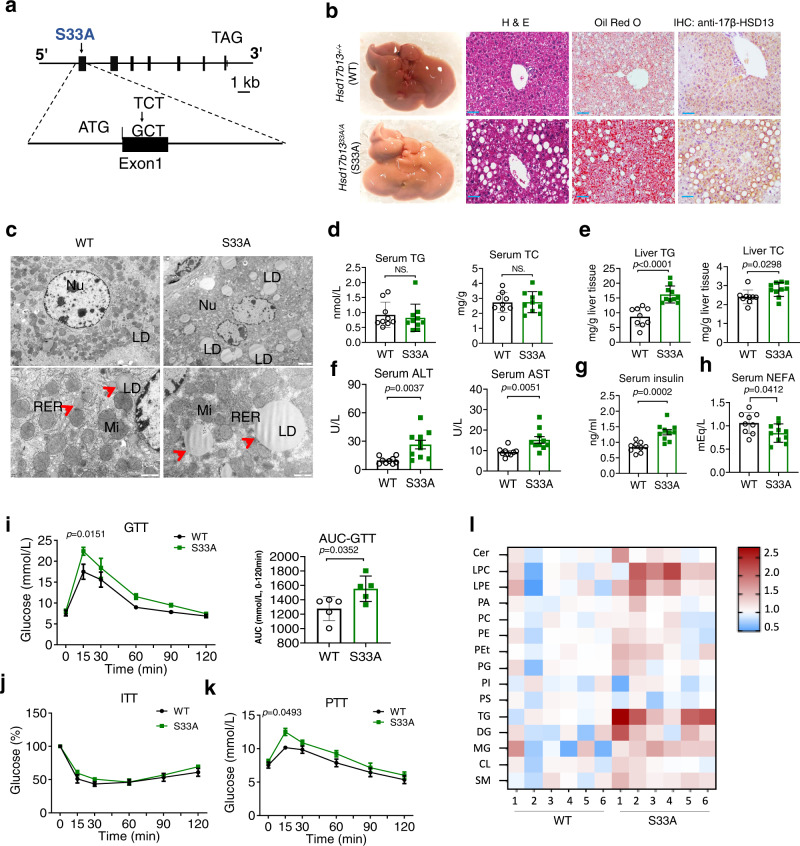
Targeted knock-in of the 17β-HSD13 S33A mutation drives NAFLD pathogenesis. **a** Schematic representation of the gene targeting strategy of 17β-HSD13 S33A. Homologous recombination was used to replace exon 1 with the corresponding mutant sequence, resulting in a substitution of alanine for serine 33 in 17β-HSD13. **b** Representative liver gross view, H&E staining, Oil red O staining, and immunohistochemistry (IHC) pictures in the *Hsd17b13*^33+/+^(WT) and *Hsd17b13*^33A/A^ (S33A) fed a chow at the age of 5 months; Scale bar, 100μm. WT: *n* = 5; S33A: *n* = 5 biologically independent animals. **c** Transmission electron microscopy (TEM) analysis of lipid droplets (LDs) in the livers of WT and S33A mice. Nu nucleus; mi mitochondria; RER Rough endoplasmic reticulum. Scale bar = 2 μm (upper panels); 1 μm (lower panels); WT: *n* = 3; S33A: *n* = 3 biologically independent animals. Serum triglyceride (TG) and cholesterol (TC) levels (**d**), liver TG and TC contents (**e**), serum ALT and AST levels (**f**), serum insulin (**g**) and serum non-esterified fatty acid (NEFA) (**h**) were analyzed in the S33A mutant mice compared with their WT littermates at the age of 5 months. WT: *n* = 9; S33A: *n* = 10 biologically independent animals. Glucose tolerance test (GTT) (**i**), insulin tolerance test (ITT) (**j**), and pyruvate tolerance test (**k**) were performed at the age of 14–15 weeks. AUC, the area under the curve. WT: *n* = 5; S33A: *n* = 5 biologically independent animals. **l** Lipidomics analysis was performed in the livers from WT and the S33A mutant mice. The expression levels of various lipids species were displayed by heat map.WT: *n* = 6; S33A: *n* = 6 biologically independent animals. Data represent mean ± SEM; Significance was calculated by two-tailed student’s t test (**d**–**h**). Two-way ANOVA with Bonferroni post hoc analysis was performed for **i**–**k**.

To determine the functional relevance of the phosphorylation of 17β-HSD13 at Ser33 in mice, a C57BL/6 mouse line with a point mutation (S33A) in the mouse *Hsd17b13* locus (*Hsd17B13*^*33A/A*^ mice) was generated by CRISPR/Cas9-mediated genome engineering (Fig. 3a). As designed, the codon 94 of *Hsd17b13* in mouse genome was altered from TCT to GCT as validated by sequencing, leading to the substitution of alanine for serine (Fig. S3a–c). The mRNA and protein levels of endogenous hepatic 17β-HSD13 were similar between *Hsd17b13*^33A/A^ mice and the littermate WT controls (*Hsd17b13*^+/+^) (Fig. S3d, e). At the age of 5 months, the *Hsd17b13*^33A/A^ mice developed a robust fatty liver phenotype (Fig. 3b). Consistently, both H&E and Oil red O staining showed a significant accumulation of large-sized LDs around the central veins in *Hsd17b13*^33A/A^ mice (Fig. 3b). Transmission electronic microscope (TEM) examination further showed that liver LD size was markedly increased in the *Hsd17b13*^*33A/A*^ mice compared with that in the *Hsd17b13*^+/+^ mice^7^ under normal diet feeding (Fig. 3c). In addition, A slight body weight gain was observed in the *Hsd17b13*^*33A/A*^ mice (Fig. S4a, b). Liver weight (%), serum TG and TC levels were comparable between WT and S33A mice (Fig. S4c and Fig. 3d). Liver TG and cholesterol (TC) contents were significantly higher in the *Hsd17b13*^33A/A^ mice than that in the *Hsd17b13*^+/+^ mice (Fig. 3e). Notably, serum ALT and AST levels in the *Hsd17b13*^*33A/A*^ mice were also increased dramatically under chow diet, indicating that more severe liver injury occurred subsequent to LD metabolism dysfunction (Fig. 3f). Body composition analysis revealed an increase in epigonadal fat (EF) but not subcutaneous fat tissue (SAT) or brown fat tissue (BAT) between two genotypes (Fig. S4d). Consistent with the liver steatosis, the *Hsd17b13*^*33A/A*^ mice increased serum insulin levels (Fig. 3g). We also observed a reduced serum nonesterified free fatty acid (NEFA) levels in the *Hsd17b13*^*33A/A*^ mice, which might be caused by inhibited adipocyte lipolysis in the presence of high serum insulin level (Fig. 3h). In accordance with the higher serum insulin level, the *Hsd17b13*^*33A/A*^ mice displayed impaired glucose tolerance compared with the *Hsd17b13*^+/+^ mice (Fig. 3i). Intriguingly, no difference in systemic insulin sensitivity was observed between two genotypes **(**Fig. 3j). These findings suggest that impaired glucose tolerance may be a result of enhanced hepatic gluconeogenesis as further supported by pyruvate tolerance test (PTT) **(**Fig. 3k) and increased expression of hepatic PEPCK expression (Fig. S4e). We then performed a lipidomic analysis of the livers. Wild-type and *Hsd17b13*^*33A/A*^ mice were sacrificed at the age of 20 weeks. Liver samples were analyzed for lipid composition (Fig. 3l). In line with increased size of LDs in the *Hsd17b13*^*33A/A*^ mouse livers, the levels of lipid species including TG, DG, lysophosphatidylcholine (LPC) and lysophospatidylethanolamines (LPE) were significantly increased. However, the levels of other lipid species were comparable between two genotypes. Our findings are consistent with a previous report in which phospholipids are also markedly altered in peoples with human *HSD17B13* SNP rs72613567^12^, suggesting HSD17B13 might play a role in phospholipid metabolism. Furthermore, immunohistochemistry study showed more F4/80-positive and CD68^+^ cell infiltration in the *Hsd17b13*^*33A/A*^ mice (Fig. S4f–h), suggesting that the 17β-HSD13 S33A mutation caused steatosis and hepatocyte injury may trigger liver inflammation. Collectively, these findings demonstrate that the impairment of the phosphorylation of 17β-HSD13 at Ser33 is sufficient to promote hepatic lipid accumulation and inflammation in mice under normal diet.

### 17β-HSD13 physically interacts with ATGL on LDs

**Fig. 4:**
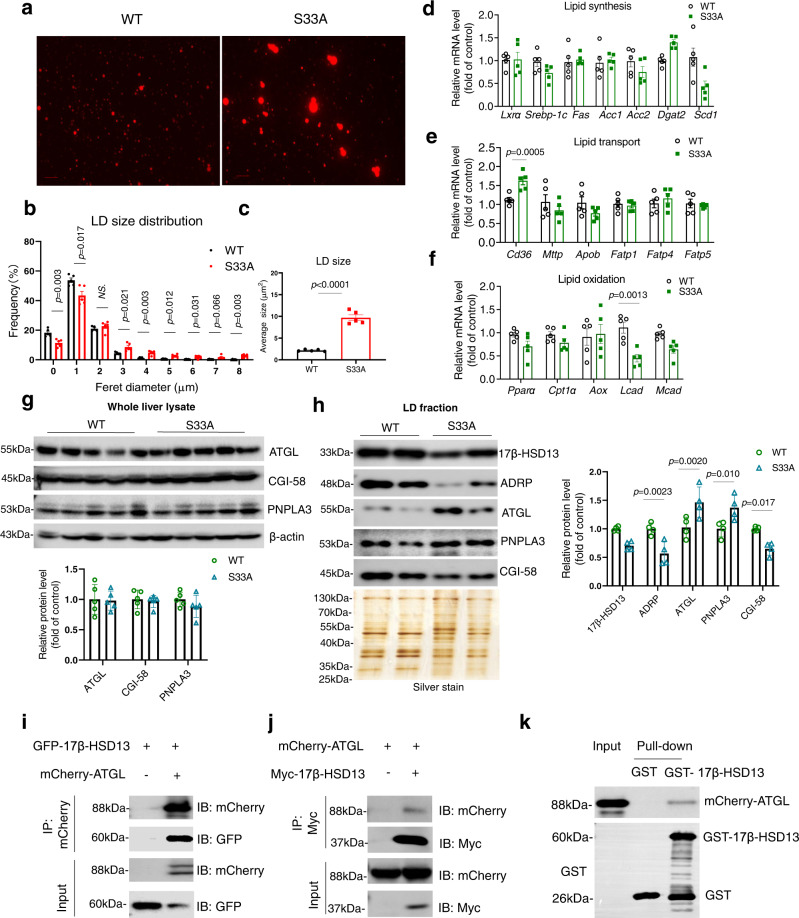
17β-HSD13 physically interacts with ATGL on lipid droplet. **a** Representative Nile red staining of purified LDs from WT and the S33A mutant mice. LD size distribution (**b**) and average LD size (**c**) were analyzed. WT: *n* = 5; S33A: *n* = 5 biologically independent animals. Quantitative RT-PCR analysis showing the expression of genes involved in lipid synthesis (**d**), lipid transport (**e**), and lipid oxidation (**f**) in the *Hsd17b13*^+/+^ and *Hsd17b13*^*33A*/A^ mice. WT: *n* = 5; S33A: *n* = 5 biologically independent animals. **g** Immunoblot assay of hepatic ATGL, PNPLA3, and CGI-58 protein expression showed no difference between two genotypes. Quantitative results were shown below. WT: *n* = 5; S33A: *n* = 5 biologically independent animals. **h** Immunoblot assay of purified LDs from the livers of the *Hsd17b13*^*+/+*^ and *Hsd17b13*^*33A/A*^ mice fed a chow diet (ND). A total of 2 μg of LD protein was immunoblotted for 17β-HSD13, ADRP, ATGL, PNPLA3, and CGI-58 protein expression. Silver staining was used as a loading control. Quantitative results were also shown. WT: *n* = 4; S33A: *n* = 4 biologically independent animals. **i**, **j** Co-IP experiments examining the interaction between 17β-HSD13 and ATGL. An expression vector carrying a GFP-tagged-17β-HSD13, myc-tagged 17β-HSD13 or mcherry-tagged ATGL (MC-ATGL) was transfected into 293T cells and cell lysates were immunoprecipitated using indicated beads, respectively. The immune precipitates were examined by immunoblotting using specific antibodies. **k** GST-pull down experiment showed 17β-HSD13 can directly bind to ATGL in vitro. Lysates of 293T cells overexpressed with mCherry-ATGL (MC-ATGL) (1 μg) were incubated with Glutathione-Sepharose beads precoated with equal amount (1 μg) of GST-17β-HSD13 or GST. The proteins pulled down by the GST-fusion proteins were analyzed by western blot. *n* = 2 biologically independent experiments for **i**–**k**. Data represent mean ± SEM; Two-way ANOVA with Bonferroni post hoc analysis was performed for **b**, (**d**–**h**), or two-tailed student’s t test (**c**).

We next purified the LDs from WT and S33A liver for further analysis. Consistent with the TEM data (Fig. 3c), size distribution analysis of liver LDs revealed a shift from small LDs (<1 μm in diameter) in wild-type mice to large LDs (>2μm in diameter) in the S33A mutant mice LDs (Fig. 4a–c). To explore the mechanism for the giant LDs in *Hsd17b13*^*33A/A*^ mice, real time PCR analysis of liver tissues were performed. The results revealed that little difference was found in the mRNA expression of genes involved in hepatic lipid synthesis, VLDL assembly, and secretion under normal diet between two genotypes. The expression of LCAD which involved in FA oxidation was slightly decreased, while the levels of CD36 responsive for FA uptake were markedly increased in the livers of the *Hsd17b13*^*33A/A*^ mice (Fig. 4d–f and Fig S4i). We anticipated that the function of PKA-induced 17β-HSD13 phosphorylation in the liver is similar to the PKA/perilipin signaling in the adipocytes, in which PKA mediates lipolysis via increased interaction between ATGL and CGI-58. Surprisingly, the protein expression levels of ATGL, CGI-58, and PNPLA3 were comparable in whole liver lysates of *Hsd17b13*^+/+^ and *Hsd17b13*^33A/A^ mice (Fig. 4g). However, immunoblotting assay showed that the protein expression levels of a few major LD-associated proteins were significantly altered in the LD fractions isolated from the *Hsd17b13*^+/+^ and *Hsd17b13*^*33A/A*^. A significant increase in ATGL and a marked reduce in CGI-58 localization were found on the surface of LDs in hepatocytes isolated from the *Hsd17b13*^*33A/A*^ mice compared to that in the *Hsd17b13*^+/+^ littermates (Fig. 4h). Since many LD-associated proteins are involved in the regulation of lipolysis via interacting with a few key regulators, especially ATGL and CGI-58/ABHD5^13^, we test whether 17β-HSD13 could directly interact with ATGL or CGI-58. We first performed co-IP experiments in 293T cells. Both in vitro, IP data and GST-pull down experiment confirmed the interaction of 17β-HSD13 with ATGL (Fig. 4i–k). These data suggest that the induction of large-sized LD in the *Hsd17b13*^*33A/A*^ mouse hepatocytes possibly occurs at the post-transcription level through dynamic regulation of the interaction of 17β-HSD13, ATGL and its co-factor CGI-58.

### 17β-HSD13 S33A mutation blocks the interaction between ATGL and CGI-58 on LDs

**Fig. 5:**
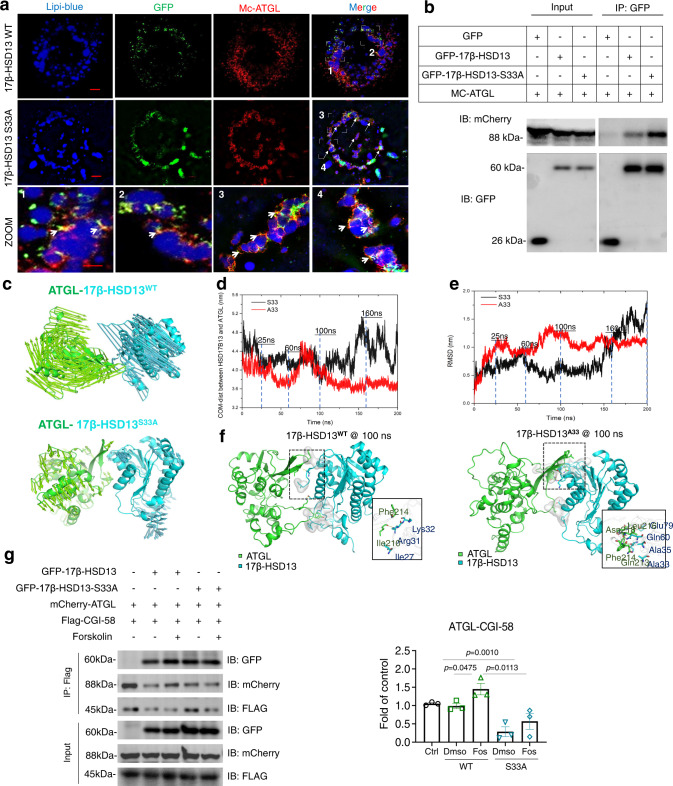
17β-HSD13 S33A mutant enhances its interaction with ATGL. **a** Huh7 cells were stably infected with the GFP-tagged 17β-HSD13 WT, GFP-tagged S33A mutant and MC-ATGL lentiviruses. 150μM oleic acid (OA) was loaded for 16 h. The cells were fixed and LDs were stained with Lipi-blue, an LD tracker. The yellow dots indicate co-localization (Scale bar = 5 μm). The zoomed pictures from each merged image were shown (Scale bar = 2 μm). *n*  = 2 biologically independent experiments. **b** The GFP-tagged 17β-HSD13 WT, S33A and MC-ATGL plasmids were transfected into 293T cells as indicated and cell lysates were immunoprecipitated using anti-GFP-beads. The immunoprecipitates were examined by immunoblotting using anti-GFP and mCherry antibodies. *n* = 2 biologically independent experiments. **c** The porcupine plots of PC1 of the 17β-HSD13 WT and 17β-HSD13 S33A system. ATGL was shown in green and 17β-HSD13^S33A^ shown in cyan. **d** Distances of center of mass along with the time traces of the simulations against the 17β-HSD13 WT and 17β-HSD13 S33A system are shown as black and red curves, respectively. **e** RMSD along with the time traces of the simulations against the 17β-HSD13 WT and 17β-HSD13 S33A system are shown as black and red curves, respectively. **f** The snapshots of the MD simulation of 17β-HSD13 WT-ATGL with the local structure around the S33 in 17β-HSD13 WT (left) and the A33 in 17β-HSD13 S33A (right) at 100 ns. **g** The expression vectors carrying Flag-CGI-58, MC-ATGL, GFP, GFP-tagged 17β-HSD13, and GFP-tagged 17β-HSD13 S33A were transfected into 293T cells, with or without forskolin treatment for 30 min. Cell lysates were immunoprecipitated using anti-flag beads. ATGL in the immune precipitates was examined by immunoblotting using an anti-mCherry antibody. GFP represents the protein levels of the 17β-HSD13 WT and S33A mutant. *n* = 3 biologically independent experiments. Data represent mean ± SEM; One-way ANOVA with Bonferroni post hoc analysis was performed for **g**.

We next thought to determine the effect of the 17β-HSD13 S33A mutation on the interaction of 17β-HSD13 and ATGL. Huh7 cells were infected with the GFP-tagged 17β-HSD13 WT, GFP-tagged 17β-HSD13 S33A and mCherry (MC)-tagged ATGL lentivirus and then stained the LDs with Lipi-blue, a lipid tracer. As previously reported^6^, MC-tagged ATGL was localized in the cytoplasm but accumulated on LDs after lipid loading (Fig. 5a and Fig. S5a). Confocal microscopy clearly showed a co-localization of 17β-HSD13 and ATGL on the surface of LDs. Interestingly, we observed that both WT and 17β-HSD13 S33A mutant can promote the translocation of ATGL from the cytosol to LD fraction (Fig. 5a). However, the S33A mutant recruited significantly more ATGL to the LDs than the WT form as reflected by enhanced co-localization of the 17β-HSD13 S33A with ATGL on the surface of LDs, suggesting distinct roles of phosphorylated and dephosphorylated 17β-HSD13 in ATGL function and hepatocyte lipolysis (Fig. 5a). In accordance with the confocal data, co-IP experiment also showed increased binding of ATGL with the 17β-HSD13 S33A mutant than WT (Fig. 5b). We further performed molecular dynamics simulation analysis. The homology modeling structures of the ATGL N-terminal domain (residues 7–252) and 17β-HSD13, and the CGI-58 structure (residues 49–349) from Alphafold2 were simulated to study the interactions with various simulation methods. By the protein surface potential and the amino acids properties analysis, the β-sheet conformation of ATGL with high hydrophobicity shows the benefit for protein-protein binding, which was confirmed from the protein docking results. The flank region of the β-sheet conformation in ATGL contributes to the main interaction with several helices structure of CGI-58 (residues 196–227), which formed a lid over the α/β hydrolase fold and the activity site of CGI-58 (as shown in Fig. S6a, b). Although the 17β-HSD13 S33A could also form stable interactions with the same region of ATGL, but was in sharp contrast to the wild type of 17β-HSD13, which exhibits significant conformational changes during the MD simulation (as shown in Fig. 5c). Compared the simulation trajectories, we found the polar side chain of S33 in 17β-HSD13 WT possesses a strong conflicting with the hydrophobic interface of ATGL which finally causes the loss of the interaction (Fig. 5c). As the porcupine plots shown in Fig. 5c, the motion tendencies of PCs from the 17β-HSD13 S33A and 17β-HSD13 WT simulation trajectories are dramatically different, and the much longer arrows in the 17β-HSD13 WT system indicate the much more drastic conformational changes (Fig. 5c and Fig. S7). Moreover, the curves of center of mass (COM) distance of the 17β-HSD13 WT and 17β-HSD13 S33A systems also demonstrate the trends. However, the 17β-HSD13 S33A mutant appears to bind more tightly to ATGL than the 17β-HSD13 WT (Fig. 5c–f and Fig. S7). Collectively, these findings demonstrate that the S33A mutant may impair LD lipolysis by enhanced binding to ATGL on the surface of LDs.

CGI-58 is an essential coenzyme for ATGL activation via direct binding. The much stronger interaction of the S33A mutant with ATGL suggests that this mutation may interfere in the interaction of CGI-58 with ATGL to a greater extent than the 17β-HSD13 WT. We suspected that 17β-HSD13 WT might mimic the function of PLIN1, a well-known suppressor of ATGL-CGI-58 interaction^14^. As expected, our results indicated that the 17β-HSD13 S33A mutant bound much stronger to ATGL than the 17β-HSD13 WT form (Fig. 5g). Moreover, the S33A mutant blocked the interaction between ATGL and CGI-58 to a greater extent than the WT form (Fig. 5g). In addition, forskolin treatment, which resulted in 17β-HSD13 S33 phosphorylation (Fig. 5g), significantly enhanced the interaction between ATGL and CGI-58 in 17β-HSD13 WT-expressing cells with little effect in the cells expressing 17β-HSD13 S33A mutant (Fig. 5g). Together, these observations indicate that PKA-mediated Ser33 phosphorylation of 17β-HSD13 increases, while the PKA-targeted S33 residue-disrupted 17β-HSD13 S33A mutation ameliorates, the binding of ATGL to CGI-58.

### Targeted mutation of the Ser33 residue in 17β-HSD13 promotes NASH development

**Fig. 6:**
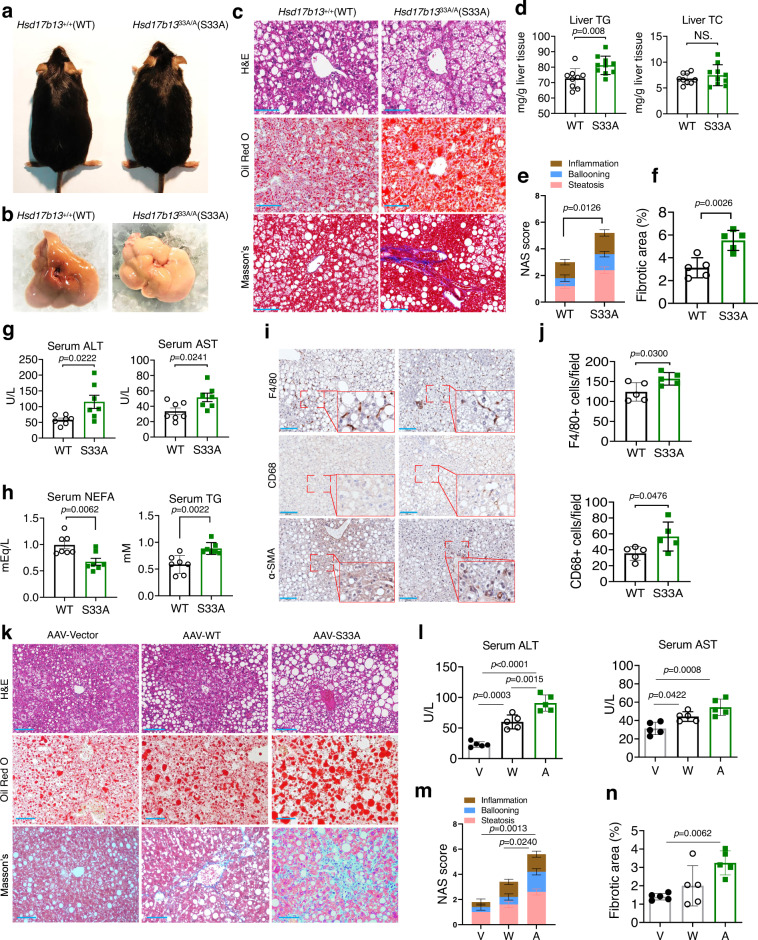
Targeted mutation of the Ser33 residue in 17β-HSD13 promotes NASH development. Representative macroscopic view (**a**), liver gross view (**b**), H&E staining, Oil red O staining, and Masson’s staining (**c**) in the *Hsd17b13*^33+/+^(WT) and *Hsd17b13*^33A/A^ (S33A) mice fed a high-fat diet for 3 months. Scale bar, 100μm. WT: *n* = 5; S33A: *n* = 5 biologically independent animals. **d** Liver TG and TC contents in HFD-treated WT and the S33A mutant mice. WT: *n* = 9; S33A: *n* = 10 biologically independent animals. The NAS score (**e**) and fibrotic area (%) (**f**) in WT and the S33A mutant mice. WT: *n* = 5; S33A: *n* = 5 biologically independent animals. Serum ALT and AST levels (**g**), and serum NEFA and TG levels (**h**) were increased in the S33A mutant mice compared with their WT littermates. WT: *n* = 9 for **g**, *n* = 7 for **h**; S33A: *n* = 10 for **g**, *n* = 7 for **h** biologically independent animals. **i**, **j** Representative immunohistochemical staining of inflammation markers (F4/80 and CD68) and fibrotic marker (α-SMA) (**i**). The semi-quantification data of F4/80 and CD68 (**j**). WT: *n* = 5; S33A: *n* = 5 biologically independent animals. **k**–**n** Global *Hsd17b13* gene deficient mice (KO) were fed with a HFD for 3 months. At the end of second month with HFD, an AAV9 empty vector (AAV-vector, v), an AAV carrying a full-length of WT 17β-HSD13 (AAV-WT, WT) or the S33A mutant (AAV-S33A, S33A) was injected via the tail vein. Representative liver H&E staining, Oil red O staining and Masson’s staining were displayed (**k**). Serum ALT and AST levels (**l**), the NAS score (**m**) and hepatic fibrotic area (%) (**n**) of mice were analyzed. AAV-V: *n* = 5; AAV-WT: *n* = 5; AAV-S33A: *n* = 5 biologically independent animals. Data represent mean ± SEM; Two-tailed student’s t test was performed for **d**–**h**, (**j**); One-way ANOVA with Bonferroni post hoc analysis was performed for **l**–**n**.

We next questioned whether the impairment of LD lipolysis affects NASH development and progression. The *Hsd17b13*^+/+^ and *Hsd17b13*^33A/A^ mice were challenged by a high-fat diet (HFD) for 3 months. Compared to the *Hsd17b13*^+/+^ mice, the *Hsd17b13*^33A/A^ mice gained more body weight and developed more severe fatty liver phenotype (Fig. 6a, b). Histological examination, Oil red O, and Masson’s staining showed that the *Hsd17b13*^33A/A^ mice displayed more severe hepatic lipid accumulation, hepatocyte ballooning, and extracellular matrix deposition than the *Hsd17b13*^+/+^ mice (Fig. 6c). Consistently, liver TG were significantly higher while liver TC unchanged in the *Hsd17b13*^33A/A^ mice than that in *Hsd17b13*^+/+^ mice (Fig. 6d). The NAS score and fibrotic area (%) measurement further identified a NASH phenotype in the S33A mutant mice (Fig. 6e, f). In accordance with the NAS score, serum ALT and AST levels were significantly elevated (Fig. 6g). Notably, serum NEFA levels were decreased while TG levels were increased (Fig. 6h). Moreover, immunohistochemical analysis revealed that inflammatory and fibrotic processes were significantly accelerated in the livers of the *Hsd17b13*^*33A/A*^ mice compared to the WT mice (Fig. 6I, j). Quantitative RT-PCR analysis showed a slight change in CD36 expression and a significant increase in collagen I α1 expression in the livers of the *Hsd17b13*^*33A/A*^ mice (Fig. S8a–e). In addition, metabolic cage studies revealed that there was no difference in whole-body energy metabolism between two genotypes by using an ANCOVA analysis^15, 16^ (Fig. S9).

Given an enhanced interaction of 17β-HSD13 with ATGL and reduced lipolysis in the *Hsd17b13*^*33A/A*^ hepatocytes, we propose that the Ser33 residue is a key phosphorylation site for 17β-HSD13 which may be involved in the regulation of ATGL-dependent lipolysis. In support, we used an AAV9 vector to construct an adeno-associated virus carrying a full-length of WT 17β-HSD13 (AAV-WT) or the S33A mutant (AAV-S33A). Adult male 17β-HSD13 gene global knockout mice (17β-HSD13^−/−^) were used and fed with a high-fat diet for 3 months. At the end of second month with HFD, the AAV9 vector, AAV-WT and AAV-S33A were injected via the tail vein, respectively. The effects of 17β-HSD13 and in particular the S33 phosphorylation site mutation were examined. In accordance with the *Hsd17b13*^*33A/A*^ transgenic mice, we found that overexpression of the 17β-HSD13 S33A mutant markedly accelerated hepatic steatosis, worsened liver function, and promoted fibrosis in the 17β-HSD13^−/−^ mice (Fig. 6k–n).

### 17β-HSD13 S33A mutant hepatocytes are resistant to PKA activation-induced lipolysis

**Fig. 7:**
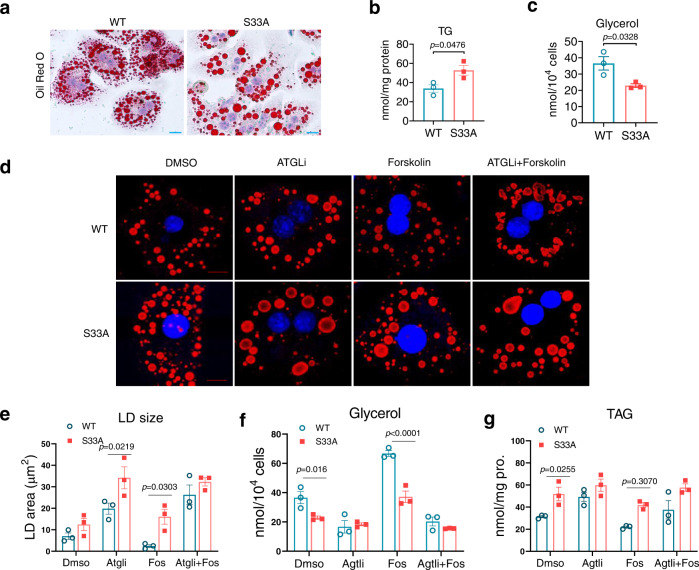
Primary hepatocytes of the *Hsd17b13*^*33A/A*^ mice are resistant to lipolysis via reducing ATGL function. **a** Primary hepatocytes from the *Hsd17b13*^*+/+*^ (WT) and *Hsd17b13*^*33A/A*^ (S33A) mice were isolated and stained for LDs with Oil Red O. At basal condition, the hepatocytes of the S33A mice exhibit larger LD size than that in the WT mice. Triglyceride contents (**b**) were higher, while the glycerol levels (**c**) were lower in culture medium of primary hepatocytes isolated from the S33A mice. WT: *n* =  3; S33A: *n* = 3 biologically independent cells. **d** Confocal micrographs showing the LDs (red) and nuclei (blue) in primary hepatocytes isolated from two genotypes treated with atglistatin, an ATGL inhibitor (ATGLi, 10 μM) and/or forskolin (10μM) for 24 h. **e** Changes of LD areas in WT and S33A mutant primary hepatocytes treated with atglistatin and/or forskolin. In WT cells, forskolin significantly reduced the LD area, which can be abolished by atglistatin treatment. In contrast, in the S33A mutant cells, both forskolin and atglistatin had little effect on the LD area. WT: *n* = 3; S33A: *n* = 3 biologically independent cells. Glycerol contents in culture medium (**f**) and triglyceride (TAG) contents in the primary hepatocytes (**g**) in **d** were examined after 24 h treatment. WT: *n* = 3; S33A: *n* = 3 biologically independent cells. Data represent mean ± SEM; Two-tailed student’s t test was performed for **b**, **c** or Two-way ANOVA with Bonferroni post hoc analysis was performed for **e**–**g**.

To further characterize the underlying mechanism by which the phosphorylation of 17β-HSD13 at Ser33 protects NAFLD, primary hepatocytes from WT and *Hsd17b13*^*33A/A*^ mouse livers were cultured and the lipolysis between two genotypes was measured. In agreement with the animal and cell line data, the *Hsd17b13*^*33A/A*^ hepatocytes displayed significant larger LDs and higher TG contents, along with markedly reduced glycerol release (Fig. 7a–c). Primary hepatocytes were then treated with the PKA agonist forskolin and an ATGL inhibitor (ATGLi). PKA activation by forskolin significantly reduced the sizes of LDs in WT hepatocytes, but not in the *Hsd17b13*^*33A/A*^ hepatocytes (Fig. 7d, e). Consistently, quantitative measurement revealed that TG content in the *Hsd17b13*^+/+^ hepatocytes was significantly reduced after forskolin treatment, but was unchanged in the *Hsd17b13*^*33A/A*^ hepatocytes. Pretreatment with the ATGLi to block ATGL-mediated lipolysis enlarged LDs in both *Hsd17b13*^+/+^ and *Hsd17b13*^*33A/A*^ hepatocytes. Furthermore, ATGL inhibition abolished the forskolin-mediated reduction in LDs size in WT hepatocytes (Fig. 7d, e). This finding is consistent with previous studies that ATGL is the major lipase controlling TG turnover in hepatocytes^17^. In addition, the *Hsd17b13*^*33A/A*^ hepatocytes produced less glycerol than *Hsd17b13*^+/+^ hepatocytes at basal condition or after foskolin treatment, the difference was abolished by the ATGLi treatment (Fig. 7f). These results indicate that the Ser33 residue is a crucial phosphorylation site of 17β-HSD13 for ATGL-mediated lipolysis in LDs and suggest that loss of PKA-mediated Ser33 phosphorylation of 17β-HSD13 is responsible for the steatosis phenotype in the *Hsd17b13*^*33A/A*^ mice. Collectively, these findings demonstrate that targeted mutation of the Ser33 residue in 17β-HSD13 accelerates HFD-induced NASH mainly via the suppression of ATGL-mediated LD lipolysis, with little impact on the expression of genes involved in hepatic lipid metabolism and whole-body energy homeostasis in mice.

### Pharmacological induction of PKA-mediated phosphorylation of 17β-HSD13 at Ser33 protects against NAFLD/NASH

**Fig. 8:**
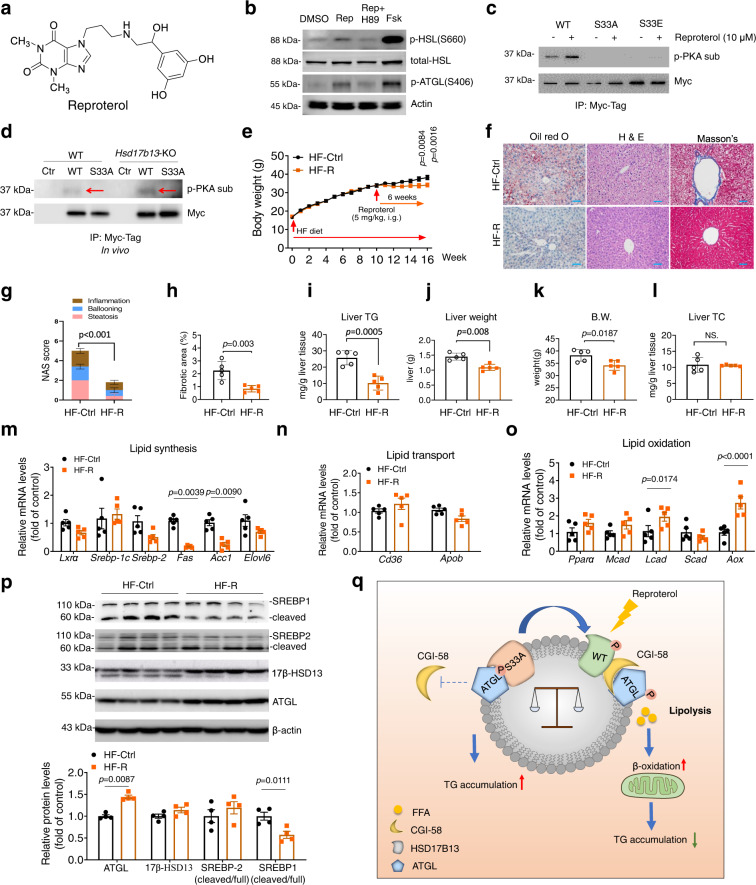
Pharmacological induction of PKA-mediated phosphorylation of 17β-HSD13 at Ser33 protects against NASH. **a** The chemical structure of reproterol. **b** Primary hepatocytes were cultured and treated with 10 μm reproterol (Rep) with or without the PKA inhibitor H89. Phosphorylated ATGL (S406), phosphorylated HSL (S660), and total HSL were immunoblotted. Forskolin (Fsk) was used as a positive control for PKA activation. **c** HEK293 cells were transfected with a full-length myc-tagged 17β-HSD13 WT, S33A mutant, and S33E mutant followed by IP with an anti-myc antibody. 10 μM reproterol or DMSO was added 30 min before cell lysis. Proteins retained on sepharose were blotted with an anti-phospho-(Ser/Thr) PKA substrate antibody. **d** Reproterol treatment increases the phosphorylation of 17β-HSD13 at serine 33 residue via PKA in vivo (see “Methods” for detail). Liver lysates were used for IP assay with an anti-myc antibody. Proteins retained on sepharose were blotted with an anti-phospho-(Ser/Thr) PKA substrate antibody. Red arrowheads indicate the phosphorylated 17β-HSD13 as a PKA-substrate. **e**–**p** Mice were fed with an HFD at 6 weeks of age for 16 weeks. Ten weeks after HFD treatment, mice began to receive reproterol treatment via intragastric administration at the dosage of 5 mg/kg body weight daily for 6 weeks. Body weight were recorded every week (**e**). HF-R, mice receiving both HF diet and reproterol; HF-Ctrl, mice only receiving HFD. HF-Ctrl: *n* = 5; HF-R: *n* = 5 biologically independent animals. Representative Oil red O, H&E, and Masson staining of the livers of HF-Ctrl and HF-R (**f**) and the NAS score (**g**) and fibrotic area (%) (**h**) in HF-Ctrl and HF-R group were shown. (scale bar, 50 μm) Liver TG (**i**) and TC content (**j**), body weight (**k**), and liver weight (**l**) in HF-Ctrl and HF-R group were measured. Expression of genes related with lipid synthesis (**m**), lipid transport (**n**), and lipid oxidation (**o**) was assessed. Protein expression levels of SREBP-1, SREPB-2, 17β-HSD13, ATGL, and β-actin in HF-Ctrl and HF-R groups were analyzed using immunoblot assay and quantitated (**p**). **q** Proposed mechanism by which 17β-HSD13 regulates LD lipolysis. Free fatty acids (FFAs) from extracellular and intracellular sources are packaged into triglycerides and stored in LDs. The S33-dephosphorylated 17β-HSD13 binds tightly to ATGL on the surface of LDs and sequesters CGI-58 to reduce ATGL lipolytic activity, leading to hepatocyte lipid accumulation. In contrast, upon PKA activation, the Ser33 residue of 17β-HSD13 is phosphorylated, which allows more physical interaction between ATGL and CGI-58 to increase ATGL activity, thereby reducing hepatocyte lipotoxicity. Data represent mean ± SEM; Significance was calculated by two-tailed student’s t test (**g**–**l**) or two-way ANOVA with Bonferroni post hoc analysis was performed for **e**, (**m**–**p**).

Since the crystal structure of 17β-HSD11, a member of the 17β-HSD family which has the highest amino acid sequence similarity with 17β-HSD13 (78% similarity), has been reported (PDB ID: 1YB1), we simulated the structure of 17β-HSD13 with the aid of Alpha Fold. We then used computer-aided drug design to screen an FDA-approved small molecular library, which yielded a few drugs with potential 17β-HSD13 binding possibility. A highly ranked drug reproterol, a β-receptor agonist and PKA activator, was chosen for further validation (Fig. 8a). Primary hepatocytes were treated with 10 μM reproterol with or without the PKA inhibitor H89. Reproterol treatment resulted in a marked increase in phosphorylated HSL (S660) and ATGL (S406), which was completely abolished by H89 (Fig. 8b). This finding suggests that reproterol can activate the PKA pathway in liver cells. To test whether reproterol treatment can phosphorylate the S33 residue in 17β-HSD13, HEK293 cells were transfected with a myc-tagged full-length WT, S33A mutant or S33E mutant of 17β-HSD13 followed by IP with an anti-myc antibody and immunoblotting with a phosphor-Ser/Thr PKA substrate antibody. Reproterol treatment for 30 min markedly induced WT 17β-HSD13 S33 phosphorylation, but failed to phosphorylate the S33A and S33E mutants in cultured cells (Fig. 8c). To further address whether reproterol can phosphorylate HSD17B13 in vivo, the AAV9 viruses were injected via tail vein for 1 month to overexpress the WT or S33A mutant form of 17β-HSD13 in the *hsd17b13* gene knockout mice. The mice then received a single intraperitoneal injection of reproterol (20 mg/kg B.W.) and were sacrificed 2 h later. The liver lysates were applied for immunoprecipitation (IP) experiment using the myc-beads. The IP products were subsequently subjected to immunoblot analysis using a PKA-substrate antibody. We found that reproterol treatment can increase the phosphorylation of wild-type form of 17β-HSD13, but failed to induce the phosphorylation of the S33A mutant 17β-HSD13 in both WT and *hsd17b13* gene deficient mice (Fig. 8d). Taken together, these results demonstrate that reproterol can induce the S33 phosphorylation of 17β-HSD13 in a PKA-dependent manner both in vitro and in vivo.

Since reproterol is capable of phosphorylating the S33 residue in 17β-HSD13, we then tested whether it affects the pathogenesis of NAFLD/NASH. Mice were fed a high-fat diet (HFD) for 16 weeks and received the treatment with or without reproterol for the last 6 weeks (Fig. 8e). HFD feeding resulted in constant body weight and liver weight gain, increased LD number and size, excessive TG accumulation, and moderate fibrosis, all of which were markedly attenuated by reproterol treatment (Fig. 8e–h). HFD-induced hepatomegaly and liver triglyceride accumulation were also reversed by reproterol treatment (Fig. 8I, j). In addition, reproterol significantly ameliorated HFD-induced obesity, glucose tolerance, and insulin resistance (Fig. 8k and Fig. S10a, b), reduced glycogen deposition (Fig. S10c) and reduced serum insulin levels (Fig. S10d), unaltered liver cholesterol contents (Fig. 8l) and serum cholesterol and triglyceride levels (Fig. S10e, f), with a marked suppression of liver glucose-6-phosphatase expression (Fig. S10g). Lipid synthesis-related genes including FAS, ACC were markedly reduced (Fig. 8m), while lipid uptake- and secretion-related genes were not significantly changed (Fig. 8n). Among the lipid oxidation genes, acyl-CoA oxidase (AOX) was increased by 3-fold, suggesting an enhanced lipid oxidation upon reproterol treatment (Fig. 8o). Immunoblotting results showed that the active form of SREBP1 was markedly reduced after reproterol treatment (Fig. 8p). In addition, ATGL protein expression levels were significantly increased, indicating an increased lipolysis in response to reproterol treatment (Fig. 8p). Collectively, these findings demonstrate that reproterol is a potent PKA-activating agent in facilitating the S33 phosphorylation of 17β-HSD13 and possesses a strong capacity in attenuating HFD-induced obesity, insulin resistance and NAFLD/NASH.

To further confirm the lipolysis-stimulating activity of reproterol, HepG2 cells were loaded with 200 μM oleic acid (OA) for 24 h with or without reproterol at the concentration of 10 μM. Reproterol significantly reduced OA-induced triglyceride accumulation (Fig. S11a). In addition, primary hepatocytes were cultured in the presence of OA to mimic steatosis and treated with reproterol for 24 h. Consistent with the in vivo data, reproterol treatment greatly suppressed the cleaved SREBP1 protein expression (Fig. S11b). With the use of a Seahorse flux analyzer, we further showed that reproterol markedly increased the mitochondrial oxidative consumption rate (OCR), suggesting reproterol can reverse FA-induced impaired mitochondrial respiration and enhance fatty acid oxidation (Fig. S11c, d). To address whether the lipolysis-stimulating property of reproterol is dependent on the S33 phosphorylation of 17β-HSD13 and increased ATGL activity, primary hepatocytes of WT and *Hsd17b13*^*33A/A*^ mice were isolated and incubated with 10 μM forskolin or 20 μM reproterol for 24 h. Both forskolin and reproterol treatment led to a marked reduction in LD accumulation in WT hepatocytes, but not in the *Hsd17b13*^*33A/A*^ hepatocytes (Fig. S11e, f). Furthermore, pretreatment of an ATGL inhibitor for 1 h completely abolished the effect of forskolin and reproterol in WT hepatocytes (Fig. S11e, f). All together, these results demonstrate that reproterol can promote hepatocyte LD lipolysis via increasing the phosphorylation of 17β-HSD13 at Ser33 in an ATGL-dependent manner. Based on the findings of the present study, we proposed the following working model. Normally, the S33 dephosphorylated 17β-HSD13 binds tightly to ATGL on the surface of LDs and sequesters CGI-58 to reduce ATGL lipolytic activity, leading to hepatocyte lipid accumulation. Upon PKA activation, the Ser33 residue of 17β-HSD13 is phosphorylated, which allows more physical interaction between ATGL and CGI-58 to increase ATGL activity, thereby enhance mitochondria respiration and β-oxidation (Fig. 8q). Therefore, increasing hepatic PKA activity or the S33 phosphorylation of 17β-HSD13 may be a promising target in NAFLD/NASH treatment.

## Discussion

In our previous work, we identified 17β-HSD13 as a liver-enriched, hepatocyte-specific LD-associated protein that is induced in the livers of mice and patients with NAFLD^7^. We further unraveled that hepatic 17β-HSD13 expression is upregulated by LXRα in an SREBP1c-dependent manner^18^ and overexpression of 17β-HSD13 leads to excessive lipid accumulation in association with increased transcriptional activity of SREBP1c^7^, suggesting a vicious cycle between 17β-HSD13 and SREBP1c in NAFLD. Recently, human genetic studies have repeatedly reported a robust association of 17β-HSD13 single nucleotide polymorphisms (SNPs) with the development and severity of NAFLD/NASH and other chronic liver diseases (CLDs)^9, 19–21^. These findings demonstrate that 17β-HSD13 may act as a potential therapeutic target for the treatment of chronic liver diseases, especially NASH. Although both preclinical and population-based GWAS studies provide compelling evidence supporting a critical role of 17β-HSD13 in NAFLD/NASH, the function and underlying mechanism of 17β-HSD13 in regulating liver lipid homeostasis remains largely unknown. More importantly, the potential of 17β-HSD13 as an intervention target for the treatment of human NAFLD/NASH also needs to be clarified.

The biogenesis and expansion of lipid droplets (LDs) are critical pathophysiological processes in the development of NAFLD/NASH. In the past years, a large body of evidence has indicated that LD-associated proteins may represent potential therapeutic targets for the treatment of NAFLD/NASH. Among them, patatin-like phospholipase domain-containing protein 3 (PNPLA3) and 17β-HSD13 have recently attracted numerous attention^9, 22, 23^. PNPLA3 possesses triglyceride hydrolase activity in hepatocytes and retinyl esterase activity in stellate cells^24^. The common I148M mutation of PNPLA3 results in loss of function of the protein with lipid retention in hepatocytes and retinol retention in hepatic stellate cells^25^. In addition, the I148M variant evades ubiquitylation and proteasomal degradation and accumulates on LDs where it competes with ATGL to interact with ABHD5 (CGI-58), leading to reduced ATGL activity^26–28^. In the present study, we identified the serine 33 (Ser33) residue of 17β-HSD13 as an evolutionally conserved cyclic AMP (cAMP)-dependent protein kinase (PKA) target site, where its phosphorylation facilitates ATGL-mediated LD lipolysis by promoting the binding of CGI-58 to ATGL on LDs in hepatocytes. Conversely, mutation of the Ser33 to Ala (S33A) leads to dephosphorylation of 17β-HSD13 at Ser33, which markedly decreases ATGL-dependent lipolysis by reducing CGI-58-mediated ATGL activation. The critical role of 17β-HSD13 phosphorylation at Ser33 in promoting hepatocyte lipolysis was further confirmed by a study using a transgenic knock-in mouse strain carrying the 17β-HSD13 S33A mutation (*Hsd17b13*^*33A/A*^). The *Hsd17b13*^*33A/A*^ mice spontaneously developed severe hepatic steatosis with reduced lipolysis, enlarged LD size, increased inflammation, and elevated ALT and AST levels and were more susceptible to high-fat diet-induced NAFLD/NASH. Together, these findings provide insight into the mechanism by which 17β-HSD13 regulates hepatocyte lipid metabolism via modulating ATGL activity on the surface of LDs at the posttranslational level. These results also suggest the possibility that pharmacological induction of the phosphorylation of 17β-HSD13 at Ser33 may have therapeutic effect on NAFLD/NASH.

To test whether the phosphorylation of 17β-HSD13 at Ser33 can serve as a potential intervention approach for NAFLD/NASH, molecular docking analysis was performed using a FDA-approved small molecule library and a simulated structure of 17β-HSD13 protein. We identified reproterol, a FDA-approved β2-adrenoceptor agonist for asthma, as a potential 17β-HSD13 modulator. Reproterol treatment can phosphorylate wild-type 17β-HSD13 at Ser 33 in a PKA-dependent manner both in vitro and in vivo. However, it failed to phosphorylate the S33A mutant. These results demonstrate that reproterol can effectively induce the Ser33 phosphorylation of 17β-HSD13 protein. As expected, treatment of reproterol significantly attenuated oleic acid-induced LD biogenesis and HFD-induced hepatic steatosis likely via increasing ATGL-mediated lipolysis and decreasing SREBP1c transcriptional activity. Multiple mechanisms may be involved in the beneficial effect of reproterol on liver steatosis. Both systemic and hepatic actions may play a role. As a β2-agonist, reproterol has a great impact on body weight and fat mass which might be helpful in attenuating excessive liver lipid accumulation. However, direct action of reproterol on liver metabolism may also contribute to its anti-seatotic effect, since reproterol treatment can increase the S33 phosphorylation of HSD17B13 in cultured hepatocyte and the livers of mice in a PKA-dependent manner. Collectively, these findings provide convincing evidence that reproterol can ameliorate NAFLD/NASH at least in part through inducing the Ser33 phosphorylation of 17β-HSD13.

It has been well known that PKA plays a crucial role in the regulation of lipid metabolism. The dysregulation of the PKA signaling is associated with the pathogenesis of various metabolic diseases including NAFLD/NASH^29, 30^. A large body of pharmacological studies shows that cAMP is produced following GPCR-mediated activation of the stimulatory Gα protein (Gαs), which in turn activates adenylate cyclase to convert ATP into cAMP, leading to PKA activation. PKA can phosphorylate the key serine residues in its protein substrates, including adenosine monophosphate-activated protein kinase (AMPK) and cAMP-response element binding protein (CREB) and hormone-sensitive lipase (HSL), all of which play a significant role in regulating liver lipid metabolism^31–33^. A recent study has demonstrated that hepatic PKA inhibition promotes liver lipid accumulation and accelerates high-fat diet-induced NAFLD^29^, suggesting PKA activation may exert hepatoprotection against NAFLD/NASH. In this study, we found the S33 residue of 17β-HSD13 can be phosphorylated by PKA, which plays a key role in 17β-HSD13 regulation of hepatic lipolysis. Reproterol, a small molecule anti-asthma drug, can elicit PKA activity and the phosphorylation of 17β-HSD13 at Ser33, leading to a marked attenuation of NAFLD. This finding provides further evidence in support of activation of hepatic PKA and in particular phosphorylation of 17β-HSD13 may serve as potential therapeutic options for NAFLD/NASH. It is worth mentioning that although reproterol is a well-tolerated selective β2-agonist which showed relatively safe profile^34^, one should be aware of many undesirable side effects including body weight and fat mass reduction and well-known cardiovascular and pleiotropic actions. Therefore, molecules targeting HSD17B13 Ser33 phosphorylation in a liver-specific manner may represent therapeutic agents in the treatment of NAFLD/NASH^35^.

In the present study, we observed a mild weight gain under basal condition in the S33A mutant mice. As we and others previously reported, HSD17B13 is a liver-enriched hepatocyte-specific lipid droplet-associated protein which is mainly expressed in hepatocytes but not in the adipose tissues^7, 36, 37^. We speculate that this extra-hepatic effect of the HSD17B13 S33A mutation is secondary to hepatic insulin resistance caused by liver steatosis, which reflects a critical role of HSD17B13 in hepatocytes, consistent with the highly selective expression of HSD17B13 in the liver. However, the exact underlying mechanism by which the HSD17B13 S33A mutant mice exhibit a slight gain of body weight and epigonadal fat mass requires further investigation.

In summary, in the present study, we identified the serine 33 (Ser33) residue of 17β-HSD13 as an evolutionally conserved PKA target site and its phosphorylation significantly facilitates lipolysis by promoting its interaction with ATGL on the lipid droplets of hepatocytes. Dephosphorylation of 17β-HSD13 at Ser33 decreased ATGL-dependent hepatocyte lipolysis both in vitro and in vivo. We also found reproterol, a potential 17β-HSD13 modulator and FDA-approved drug, conferred a protection against NAFLD/NASH via PKA-mediated Ser33 phosphorylation of 17β-HSD13. Therefore, pharmacological induction of the Ser33 phosphorylation of 17β-HSD13 could represent an approach to treat NAFLD/NASH.

## Methods

### Animal

All animals were maintained and used in accordance with the research ethics guidelines of the Institutional Animal Care and Use Committee of Shenzhen University Health Science Center and Animal Experimentation Ethics Committee of Shenzhen University Health Science Center. The studies were approved of the Institutional Animal Care and Use Committee of Shenzhen University Health Science Center and Animal Experimentation Ethics Committee of Shenzhen University Health Science Center (AEWC-202200018).

17β-HSD13 S33A mutant mice (*Hsd17b13*^*33A/A*^) of C57BL/6J background were generated by CRISPR/Cas-mediated genome engineering (Cyagen Biosciences). The S33 located on exon 1 was selected as a target site. Cas9 mRNA, gRNA targeting vector to mouse *hsd17b13* gene, and donor oligo were designed and co-injected into fertilized eggs to generate targeted knockin offspring. The S33A (TCT to GCT) mutation sites in donor oligo was introduced into exon 1 by homology-directed repair. The gRNA target sequence is: 5′CGGAGAAGGAAATCTGTGACCGG3′. Primers used for PCR include the followings: F1 (5′ CTCTGAGCAGAGGTGTCTGTTGTGAG3′) and R1 (5′CGTTGGTTCCATCTTGTTCACCTG3′). The expected transgene product size is 418 bp. The genotype was identified by sequencing with a sequence primer (5′ AAAGCCAGCACTAAGTTACGTCTCT3′). Global *Hsd17b13* gene deficient (*hsd17b13*^−/−^) mice of C57BL/6J background were constructed using the embryo micro-injection method (GemPharmatech, China).

Male mice were used in the study and treated as described in the Figure legends. 6–8 weeks male *Hsd17b13*^*33A/A*^ mice and age matched littermate control (WT) were fed either normal chow diet (Guangdong Medical Laboratory Animal Center, #AIN-93M) (WT: *n* = 9; S33A: *n* = 10) or a 45 kcal% HFD (Research Diets, #D12451) (WT: *n* = 9; S33A: *n* = 10) for 3 months. For the AAV overexpression experiment, 8-week global *hsd17b13*^−^^/−^ gene deficient (KO) mice were used and treated with HFD for 2 months before injected with an AAV9 empty vector (AAV-vector-myc, V, *n* = 5), an AAV carrying a full-length of WT 17β-HSD13 (AAV-WT-myc, WT, *n* = 5) or the S33A mutant (AAV-S33A-myc, S33A, *n* = 5) via the tail vein for one month. For the reproterol experiment, C57BL/6J mice were fed a high-fat diet (HFD) for 16 weeks and received the treatment with or without reproterol for the last 6 weeks at the dose of 5 mg/kg B.W per day (*n* = 5 per group). The experimental animals were monitored every day and weighted every week. If there were changes in physical condition such as loss of body weight, abnormal coat condition or posture; lameness; excessive licking or scratching of any mice, we will give more frequent observations. Humane endpoint was set includes rapid weight loss of 15–20 percent within a few days, dehydration, loss of ability to ambulate (inability to access food or water). Since our NAFLD model is to treat animal with high fat diet which induces body weight increase and the drug reproterol is an FDA-approved drug which exerts litter side effect, we did not observe any mice reached the humane endpoint. The mice were euthanized with CO_2_ inhalation at the end of experiment. All mice were maintained in a controlled environment of 20–23 °C, 50–60% humidity, with a 12/12 h light/dark cycle, and permitted *ad libitum* consumption of water and diet, except for fasting experiments.

### Immunoprecipitation

293 cells and Huh7 cells expressing GFP, GFP-17β-HSD13 WT, GFP-17β-HSD13 S33A mutant, S61A mutant, myc-17β-HSD13 WT, S33A mutant, S33E mutant were harvest and proceeded for immunoprecipitation as indicated. Briefly, the cells were lysed with ice-cold lysis buffer (P0013; Beyotime) supplemented with protease inhibitor cocktail (Roche) and Phos-stop (Roche) for 30 min on ice. After being centrifuged at 12,000 × *g* for 10 min at 4 °C, insoluble material was removed and the lysates were incubated with indicated anti-GFP or anti-mCherry beads for 1 h, followed by washing 5 times with a washing buffer (containing 250 mM NaCl). The beads were then boiled with the loading buffer and analyzed by western blot.

### In vitro PKA Cα phosphorylation assay

The in vitro kinase assay was undertaken as previously reported^38^. Briefly, 30 μl immunoprecipitated 17β-HSD13 product was incubated with 0.15 μg purified recombinant active PKA C*α* protein in the kinase buffer (35 mM Tris-HCl, pH 7.5, 10 mM MgCl_2_, 0.5 mM EGTA, and 0.1 mM CaCl_2_) containing 100 μM ATP for 30 min at 30 °C in a final volume of 40 μl. Reactions were terminated by addition of concentrated sample buffer and proceeded to western blot.

### Identification of the phosphorylation sites by LS-MS/MS

A GFP-tagged full-length wild-type 17β-HSD13 was transiently expressed in 293T cells by transfection. After incubation with foskolin for 30 min, the cells were harvested and proteins were released with the RIPA buffer, and then immunoprecipitated (IP) using anti-GFP beads (KTSM1301, Shenzhen Health Life Tec.). Proteins from IP were separated by SDS-PAGE and stained with Coomasie brilliant blue and then cut for MS assay. MS assay was performed by Wininnovate Bio., Shenzhen. The protein band containing the GFP-17β-HSD13 was cut, washed 3 times with ddH2O, and destained. Proteins were then digested in the gel by Trypsin Gold (V5280, Promega Biotech Ltd.) for 8 h at 37 °C. Peptides were extracted from the gels with 70% acetonitrile (0.02% trifluoroacetic acid), lyophilized, and re-suspended in ddH2O (0.1% formic acid). 2 μL of the peptide mixture was loaded onto a nanoViper C18 (Acclaim PepMap 100, 75 μm × 2 cm) trap column, and online chromatography separated on the Easy nLC 1200 system (ThermoFisher, USA). Tandem mass spectra (MS) were acquired on a Q Exactive mass spectrometer (ThermoFisher, USA) equipped with a Nano Flex ion source. The MS/MS data were analyzed for protein identification using PEAKS Studio 8.5. Mass tolerance for precursor and fragment were set at 10 ppm and 0.05 Da, respectively. Phospho-peptides and phosphor-sites localization was carried out using the Mascot Server, where the neutral loss of phosphate (H3PO4, 98 Da, or HPO3, 80 Da) in intact peptide was a characteristic indicator for the identification of the phosphorylated residues.

### Protein modeling

The homology modeling was conducted by Modeller 9.24^39^ with multiple structures as the templates. For the N-terminal domain (residues 7-252) of ATGL (UNP ID: Q96AD5), crystal structures from the patatin-like phospholipase domain family (InterPro: IPR002641) were used as the templates, including 1OXW, 3TU3, 4AKF, and 4KYI. For 17β-HSD13, crystal structures from other 17-beta-hydroxysteroid dehydrogenases were used as the building templates, including 1A27, 1YB1, 5EN4, and 4FAL. The modeling results were rescored with DFIRE2, and the model with the top-rank of DFIRE2^40^ and DOPE scores was selected to run a 100 ns molecular dynamics (MD) simulation to relax the conformation. The Alphafold2 structure (Jumper et al. 2021) of CGI-58 (residues 49-349) was taken to run a 100 ns MD simulation to relax the conformation for the further study.

### Protein–protein interaction prediction

The relaxed modeling structures from MD simulations were used for predicting the protein–protein interaction. The APBS software^41^ and VMD 1.9.4^42^ were used to calculate the electricity of protein surface. The protein-protein docking process was conducted with Zdock 3.0.2^43^, and the Rosetta clustering protocol^44^ was used to cluster and rescore the complex structures. For each system, the complex structure with the top-rank Rosetta score and Zdock score in the largest cluster was selected to do the further MD simulation and structural analysis.

### Molecular dynamics simulation

All of the MD simulations were performed by Gromacs 2020.4 with Amber14 force field^45^. The complex was solvated in a cubic TIP3P water box with 1 nm distance from the edge, which was neutralized by sodium ions. After two steps of energy minimization, the temperature of the system was gradually heated to 300 K over 100 ps to perform the 2 ns NVT equilibration and 5 ns NPT equilibration subsequently. Finally, the 200 ns MD simulations at 300 K and 1 atm were carried out with the LINCS algorithm to restrain the hydrogen positions at their equilibrium distances, which allowed the use of an integration time step of 2 fs.

### Lipid droplet isolation

Liver lipid droplets (LD) were purified as described previously^7^. Briefly, liver tissues (left lobe) from the 17β-HSD13 WT (*Hsd17b13*^*33+/+*^) and 17β-HSD13 S33A (*Hsd17b13*^*33A/A*^) mice were cut into small pieces in ice-cold PBS containing 0.2 mM PMSF and connective tissue were discarded. Then, the liver tissues were transferred to 10 ml buffer A (25 mM tricine, pH 7.6, 250 mM sucrose) plus 0.2 mM PMSF and homogenized with a Dounce type glass-Teflon homogenizer on ice for 10 times followed by centrifugation at 3000 × *g* for 10 min. The supernatant was loaded into an SW40 Ti tube and loaded with 5 ml Buffer B (20 mM Hepes, pH 7.4, 100 mM KCl, and 2 mM MgCl_2_). The samples were then centrifuged at 8000 × *g* for 1 h at 4 °C. The LD fraction on the top of the gradient was collected and washed for 3 times. 10 μl of LDs at the last wash was stained with Nile red for 5 min. The morphology of LDs was examined by a Nikon Eclipse Ti microscope. Protein was extracted for western blot analysis.

### Glucose tolerance test, insulin tolerance test and pyruvate tolerance test

For glucose tolerance test (GTT), mice were starved for 2h^46^, followed by intraperitoneal injection of glucose (1.5 g/kg body weight). For insulin tolerance test (ITT)^47^, mice were fasted for 2 h prior to the test and received an injection of human regular insulin (0.5 U/kg body weight). For pyruvate tolerance test (PTT), mice were fasted for 6 h prior to the test and received an injection of pyruvate (2 g/kg body weight). Blood glucose levels were recorded at 0, 30, 60, 90, and 120 min using a glucometer.

### Cell experiments

The 293T cell line, HepG2 cell line and Huh7 cell line were purchased from the Cell Bank of Chinese Academy of Sciences (Shanghai, China). and maintained in complete minimum essential medium (10% FBS, penicillin/streptomycin, minimal essential amino acids) at 37 °C in 5% CO_2_. The expression vectors encoding a full-length wild-type human 17β-HSD13, 17β-HSD13 S33A mutant, and ATGL were generated by cloning the corresponding cDNA sequences into the pLVX vector. To generate lentivirus, the vectors encoding human pLVX-17β-HSD13, pLVX-17β-HSD13 S33A mutant, Plvx-ATGL were co-transfected with psPAX2 and pMD2.G into 293T cells. After the cells were incubated at 37 °C, 5% CO_2_ for 48–72 h, the media containing lentiviral particles were harvested. For lentiviral infection, Huh7 cells or HepG2 cells were cultured in basal medium until 70% confluence and then infected with indicated lentivirus-containing medium for overnight in the presence of 8 μg ml^−1^ polybrene. For the lipid droplets morphology analysis, Huh7 cells infected with the GFP, GFP-tagged 17β-HSD13 WT, or GFP-tagged 17β-HSD13 S33A lentivirus with or without mCherry-tagged ATGL lentivirus were seeded in 12-well plate. The cells were fixed and stained with Nile red, Lipi-blue, or DAPI (as specified in each experiment), then examined with ZEISS-LSM880. The 3D reconstruction and video of the GFP-17β-HSD13 WT and GFP-17β-HSD13 S33A were acquired using a Nikon-SIM microscope.

### Western blot analysis

Total protein was prepared as described before^48^. A total of 40 μg of protein were separated by 10% SDS-PAGE and transferred to a PVDF membrane. After being blocked with 5% skim milk, the membrane was subsequently incubated with indicated primary antibodies overnight at 4 °C. After being washed three times, the membrane was incubated for 1 h at room temperature with horseradish peroxidase (HRP)-conjugated secondary antibodies.

### RNA extraction and quantitative RT-PCR

Total RNA was isolated from mouse tissues or HepG2 cells using Trizol method (Takara). Two micrograms of total RNA were used for reverse transcription using the RevertAid First Strand cDNA synthesis kit (Thermo). SYBR Green-based real-time PCR was performed using LightCycler 96 (Roche) with SYBR Premix Ex Taq II (Takara). The quantity of mRNA was calculated using the ΔΔCt method. All reactions were performed in duplicate. The primers used for quantitative PCR are listed in Supplementary Table 1.

### Histology, immunohistochemistry, scanning, and analysis

Liver samples were fixed in 4% paraformaldehyde and paraffin-embedded at Department of Pathology Core of Shenzhen University. 5μm sections were prepared and stained with Hematoxylin/Eosin or specific antibodies as indicated. Slides were scanned with a digital slice scanner (OCUS, GRUNDIUM) and semi-quantified with the image J software. the NAS score was examined by a board-certified pathologist by using H&E and Masson’s Trichrome staining according to Kleiner^49^.

### Antibodies, drugs, plasmids, and vectors

For the antibodies, phospho-PKA substrate antibody (catalog no. 9624, 1:1000 dilution), phospho-HSL (Ser-660) antibody (catalog no. 4126, 1:1000 dilution), myc (catalog no. 2276, 1:2000 dilution) and ATGL antibody (catalog no. 2138, 1:1000 dilution) were from Cell Signaling Technology. The phospho-ATGL (Ser-406) antibody (catalog no. 135093, 1:1000 dilution), ADRP (catalog no. ab181452, 1:2000 dilution), PNPLA3 (catalog no. ab81874, 1:1000 dilution), CGI-58 (catalog no. ab183739, 1:1000 dilution), β-actin (catalog no. ab8226, 1:2000 dilution), F4/80 (catalog no. ab6640, 1:400 dilution for IHC staining), α-SMA (catalog no. ab5694, 1:400 dilution for IHC staining) and CD68 (catalog no. ab125212, 1:400 dilution for IHC staining) were from Abcam. GFP (catalog no. GTX113617, 1:2000 dilution), mCherry (catalog no. GTX128508, 1:2000 dilution) were from Genetex, 17β-HSD13 (OAAN01691, 1:1000 dilution) was from Aviva. The HRP conjugated secondary antibodies goat anti mouse IgG (ZB-2305, 1:10000 dilution) and goat anti mouse IgG (ZB-2301, 1:4000 dilution) were from ZSGB-BIO. Oil Red O (catalog no. O0625), isoproterenol (catalog no. I2760), forskolin (catalog no. F6886), IBMX (catalog no. I7018), and H89 (catalog no. B1427), Nile red were from Sigma, Lipi-Blue (LD01, Dojindo), the ATGL inhibitor atglistatin (catalog no. 15284) and the HSL inhibitor CAY10499 (CAS catalog no. 359714-55-9) were from MCE.

### Primary hepatocyte culture

Primary hepatocytes were isolated from the livers of male WT and *Hsd17b13*^*33A/A*^ mice (8 weeks old) on C57BL/6J background as previously reported^7^. Briefly, mice were anesthetized with an intraperitoneal injection of bromethol. The livers were subjected to a two-step collagenase perfusion through the portal vein and were excised. Hepatocytes were lashed out and filtered through a nylon filter (Falcom). The cells were washed with ice-cold RPMI 1640 for 3 times. Cell viability was usually 85–95%, as evaluated by trypan blue exclusion. Then hepatocytes were counted and seeded in rat tail collagen-coated plates and cultured with RPMI 1640 medium containing 10% (vol/vol) FBS, 2.5 nM insulin, 1 nM dexamethasone, 50 U/ml penicillin, and 50 μg/ml streptomycin. After 3 h, unattached cells were washed away with PBS and added with fresh medium for subsequent analysis.

### Measurement of liver triglycerides and cholesterol

Total liver triglyceride (TG) and cholesterol (CHO) were extracted from mouse liver and quantitated by use of TG and CHO assay kits (Applygen Technologies, Inc, Beijing, China) according to the manufacturer’s instructions.

### Analytical procedures and chemicals

The serum was collected for analysis of TG (Biosino), TC (Biosino), alanine aminotransferase (ALT) (Nanjing Jiancheng), and aspartate aminotransaminase (AST) (Nanjing Jiancheng) and insulin (Alpco). All measurements were performed according to the manufacturer’s instructions.

### Seahorse analysis

Cellular respiration rates were performed using an XF24 flux analyzer (Seahorse Bioscience Inc. North Billerica, MA, USA) following the manufacturer’s instructions.

### The Mito stress test

The OCR of the Huh7 cells stably expressing either wild-type (WT) or S33A HSD17B13 was measured using the Seahorse XF24 Extracellular Flux Analyzer (Agilent Technologies) according to the manufacturer’s protocol. The cells were plated onto XF24 cell culture micro plates at a density of 2.5 × 10^4^ per well (Agilent Technologies). The cells incubated in the XF base medium supplemented with 1 mM pyruvate, 2 mM glutamine and 10 mM glucose were moved to a CO_2_-free incubator 1 h prior to the assay. The OCR was measured using the Mito Stress Test Kit (Agilent Technologies) with following additions: oligomycin (1 μM), FCCP (1 μM), and rotenone/antimycin A (0.5 μM). Three 3 min cycles were measured for each condition.

### The substrate oxidation stress test

The Huh7 cells stably expressing either wild-type (WT) or S33A HSD17B13 were plated and grown overnight, followed by a serum starvation for 4 h in the XF base medium (Agilent Technologies) supplemented with 0.5 mM glucose, 1 mM glutamine and 0.5 mM carnitine. The cells were then moved to a CO2-free incubator and incubated for another 1 h in the XF base medium supplemented with 0.5 mM glucose and 0.5 mM carnitine, after which the cells were treated with 0.17 mM fatty acid (FA)-free BSA or 0.16 mM palmitic acid (Sigma-Aldrich) conjugated with the FA-free BSA. The OCR was measured using the Substrate Oxidation Stress Test Kit (103672-100, Agilent Technologies) with following additions: etomoxir (6 μM), oligomycin (1 μM), FCCP (1 μM), and rotenone/antimycin A (0.5 μM). Three 3 min cycles were measured for each condition. The data were analyzed using online web-based Agilent Seahorse software platform which automatically calculates the XF Palmitate Oxidation Stress Test parameters including basal respiration, acute response to inhibitor, maximal respiration, and maximal response.

### Electron microscopy

Fresh liver tissues from WT and *Hsd17b13*^*33A/A*^ mice were dissected, minced into 1 mm^3^ cubes, and prefixed in 3% glutaraldehyde for 2 days at 4 °C. Post-fixation was performed in 1% osmium tetroxide in Millonig buffer for 1 h at 4 °C followed by dehydration in an ascending concentration series of acetone at room temperature. The tissues were embedded in epoxy resin and polymerized at 60 °C for 48 h. Ultrathin sections were cut and mounted on naked copper grids before staining with 2% uranyl acetate and lead citrate solutions then examined under a transmission electron microscope (JEM-1400, Japan). 10 random cells (electron microscopy) or microscopic fields (light microscopy) from 3 WT or 3 *Hsd17b13*^*33A/A*^ mice were examined and reported by an experienced EM pathologist in a blind analysis.

### Metabolic cages

Mice were individually housed in Harvard Metabolic cages for 3 days before data collection. In direct calorimetric and energy balance parameters including VO_2_ (oxygen consumption, in l/min), VCO_2_ (carbon dioxide expiration, in l/min) and food intake were assessed for four days. Energy expenditure (EE, in kJ/min) was calculated by using the Weir’s equation. Values of EE were normalized to the body weight raised to the power 0.75.

### Lipidomic analyses

Lipids of each sample was extracted by chloroform methanol (vol/vol, 2:1) method and dry in vacuum. Samples were dissolved in isopropanol for LC-MS (Suzhou Bionovogene). Chromatographic separation was accomplished in a Thermo Vanquish system equipped with an ACQUITY UPLC BEH C18 (100 × 2.1 mm, 1.7 μm, Waters) column. The ESI-MSn experiments were executed on the Thermo Q Exactive Focus mass spectrometer with the spray voltage of 3.5 kV and −2.5 kV in positive and negative modes, respectively. analyzed and identified using LipidSearch software. 20 μL liquid from each sample was used as quality control (QC).

### Statistical analysis

All results are given as means ± SEMs. Prism software (version 8.4.2, GraphPad Software, Inc) was used to assess statistical significance between two groups. Unpaired two-tailed Student’s t-test or Two-way analysis ANOVA followed by post hoc Bonferroni test for multiple groups comparisons calculated in GraphPad Prism 8 version 8.4.2. A *p* value < 0.05 was considered as significant. N values are also indicated within figure legends and refer to biological replicates.

### Reporting summary

Further information on research design is available in the Nature Research Reporting Summary linked to this article.

## Supplementary information

Supplementary Information

Reporting Summary

## Data Availability

A reporting summary for this article is available as Supplementary Information file. All data generated or analyzed during this study are included in this published article. The phosphorylation MS data has been submitted to PRIDE (PXD037262). The lipidomic data generated in the study have been deposited in the Metabolights under accession code MTBLS6094. The other source data are provided as a Source Data file. Source data are provided with this paper.

## Source data

Source Data
