# Supplementary material for: Phosphorylation of 17β-hydroxysteroid dehydrogenase 13 at serine 33 attenuates nonalcoholic fatty liver disease in mice
Source: Nat Commun. 2022 Nov 2;13:6577. doi: 10.1038/s41467-022-34299-1 (PMC9630536; doi:10.1038/s41467-022-34299-1)

Phosphorylation of 17β-hydroxysteroid dehydrogenase 13 at serine 33 attenuates nonalcoholic fatty liver disease in mice

Supplemental figures

a

GFP-17β-HSD13

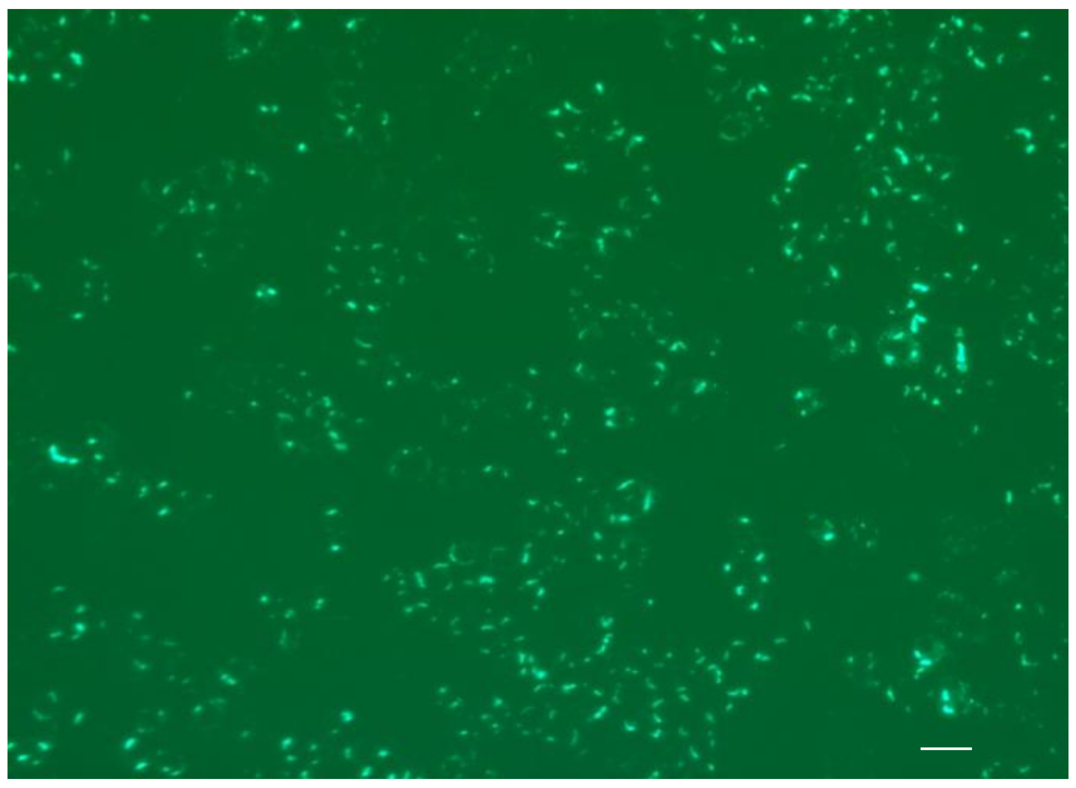

293T

Figure S1. Expression of a GFP-tagged 17β-HSD13-WT protein in HEK293 cells. Scale bar=100μm.

Figure S2.

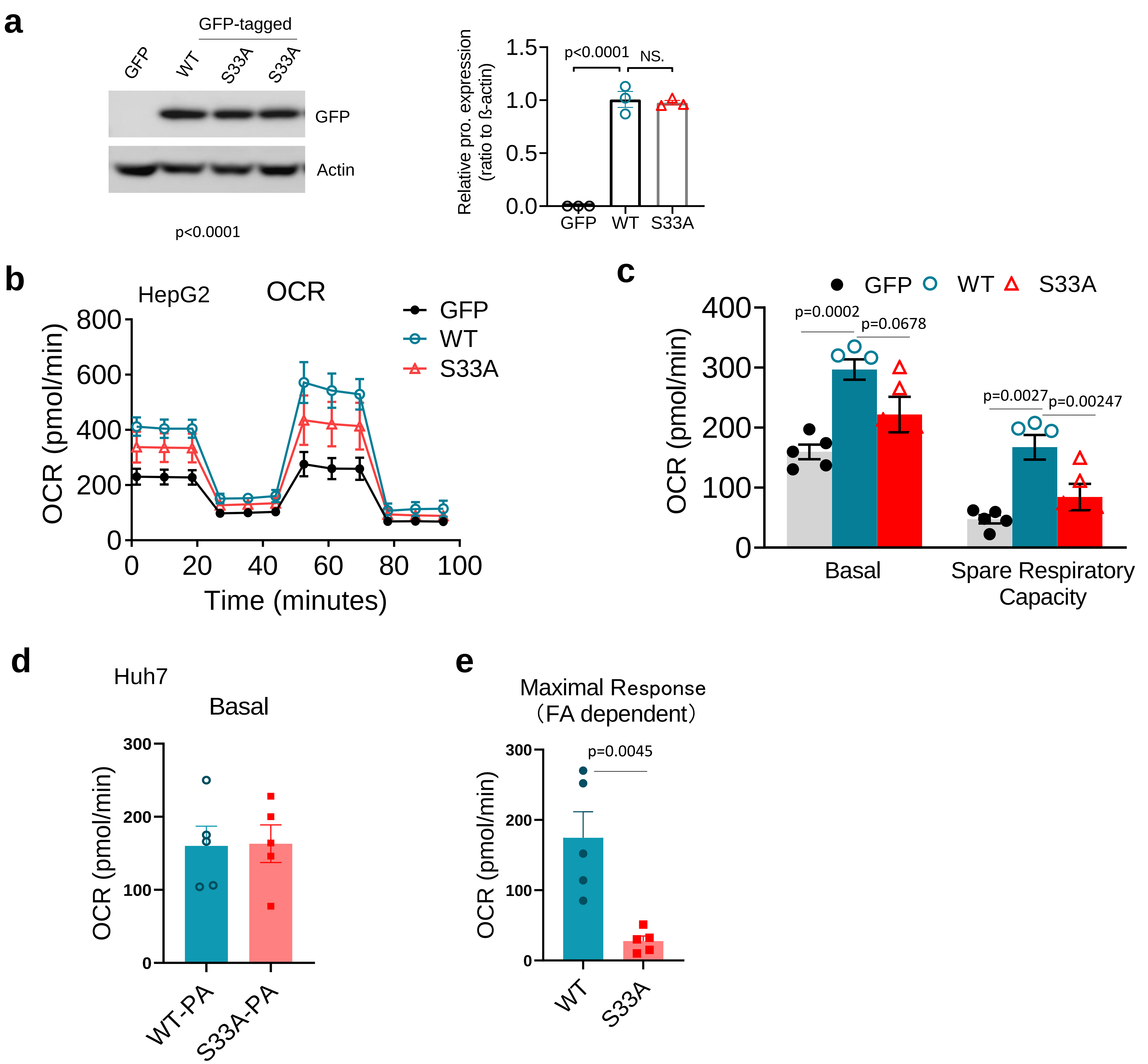

Figure S2. The 17β-HSD13-S33A mutant cells are resistant to lipolysis.

(a) Huh7 cells were infected with GFP, GFP-tagged 17β-HSD13-WT (WT) or 17β-HSD13-S33A (S33A) lentiviruses. Exogenous HSD17B13 protein expression levels were examined with an anti-GFP antibody and quantified. GFP: *n* = 3; WT: *n* = 3; S33A: *n* = 3 biologically independent cells.

(b&c) HepG2 cells were stably infected with the lentiviruses carrying a full-length GFP (GFP), GFP-tagged 17β-HSD13-WT (WT) or GFP-tagged 17β-HSD13-S33A mutant (S33A). Mitochondrial respiration was analyzed in real-time using the Seahorse XF24 Extracellular Flux Analyzer. The oxygen consumption rate (OCR) (**b**) at different stages of respiration, basal and spare respiratory capacity (**c**) were measured as described in the "Methods" section. GFP: *n*=5; WT: *n* = 4; S33A: *n* = 4 biologically independent cells.

(d&e) Oxygen consumption rate (OCR) in the presence of exogenous palmitic acid in WT and the S33A mutant Huh7 cells. Advanced ORC was measured in real-time using the Seahorse® metabolic flux analyzer as described in the section of "Materials and Methods". (**d**) Basal OCR due to utilization of exogenous fatty acids in the WT-PA group and S33A-PA group. (**e**) Maximal respiration due to the addition of FCCP. WT-PA: *n*=5; Medium-S33A-PA: *n*=4.

Data represent mean ± SEM; Significance was calculated by one-way ANOVA with Bonferroni post hoc analysis (**a, c**) or two-tailed student's t test (**d-e**).

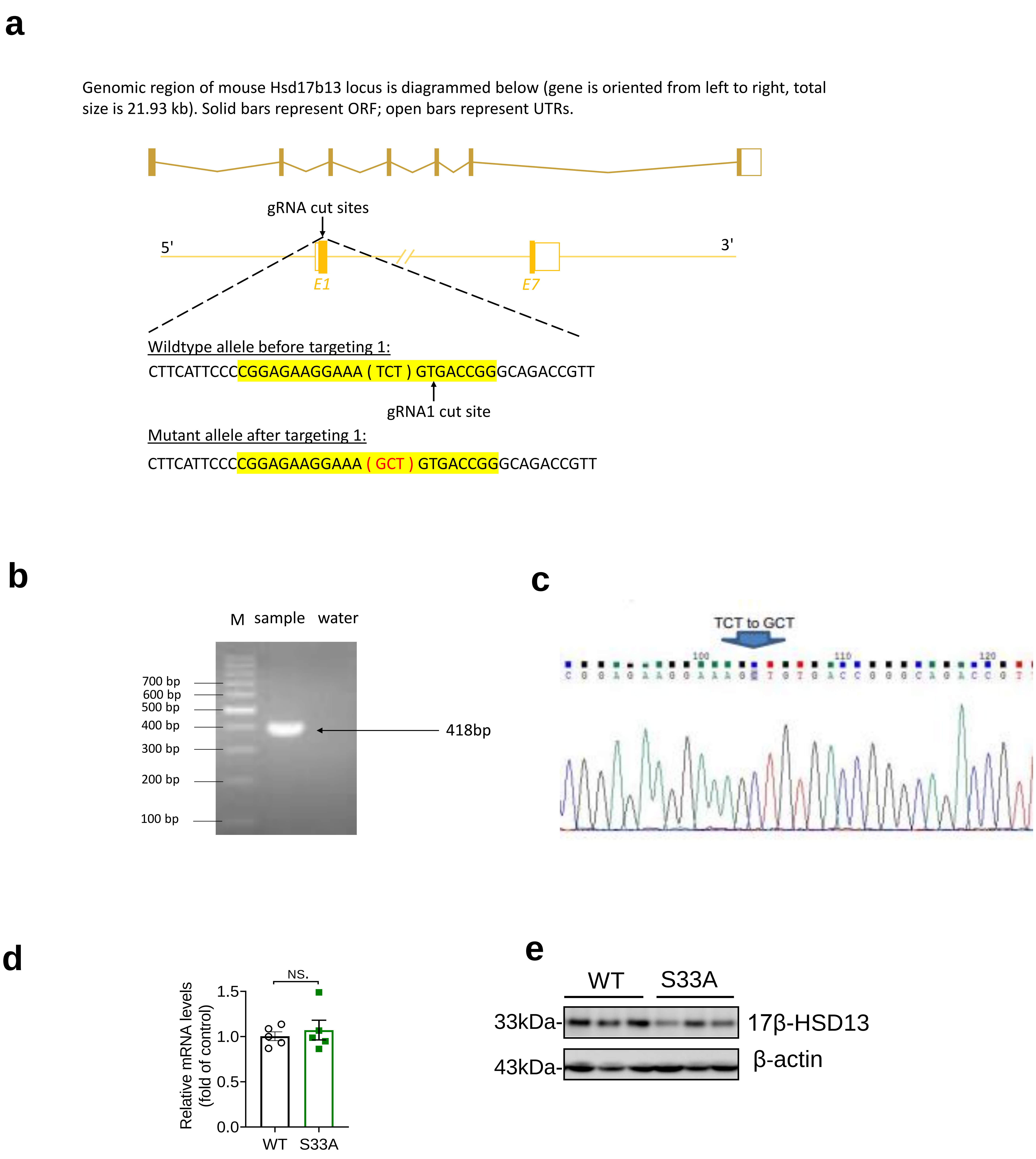

Fig. S3. Generation and genotyping of 17β-HSD13-S33A knockin mouse.
(a) A knockin mouse with a point mutation (S33A) in *hsd17b13* gene on C57BL/6 background was created by CRISPR/Cas9-mediated genome engineering.
(b) PCR-based genotyping of the mouse with the S33A point mutation.
(c) PCR product was sequenced to validate the genotype of homozygous mice (*Hsd17B13*$^{33A/A}$). The sequence primer used for PCR product is 5'-AAA GCC AGC ACT AAT TTA CGT CTCT-3'.
(**d&e**) 17β-HSD13 mRNA (**d**) (WT: *n* = 5; S33A: *n* = 5 biologically independent animals) and protein levels (**e**) (WT: *n* =3; S33A: *n* = 3 biologically independent animals) in the livers of male (12-week old) *Hsd17B13*$^{+/+}$ (WT) and *Hsd17B13*$^{33A/A}$ (S33A) mice fed a chow diet.
Data represent mean ± SEM; Significance was calculated by two-tailed student's t test.

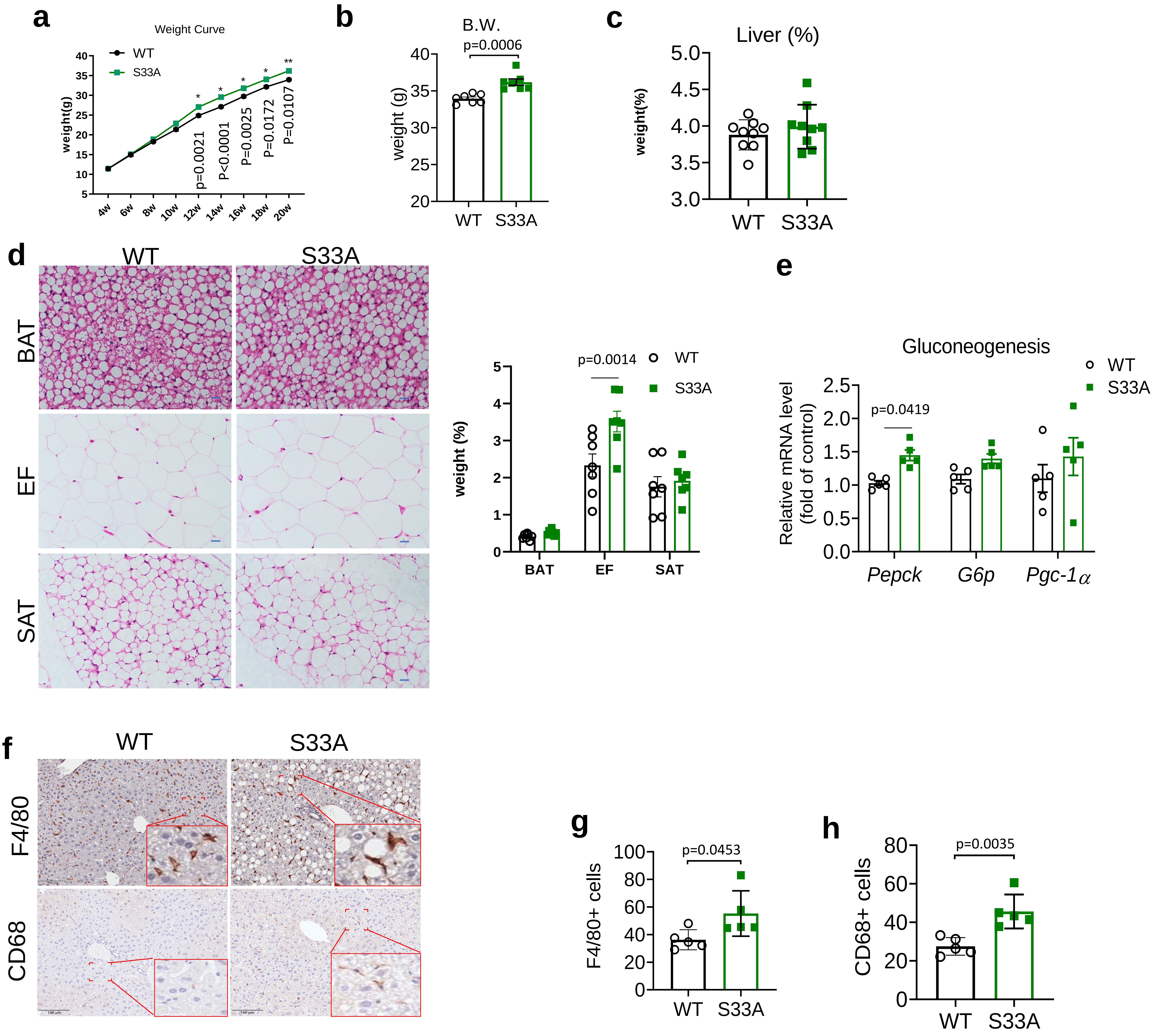

Figure S4. Metabolic parameters of the *Hsd17b13*$^{+/+}$ and *Hsd17b13*$^{33A/A}$ mice.

Hsd17b13$^{+/+}$ (WT) and *Hsd17b13*$^{33A/A}$ (S33A) mice were fed a chow diet for 5 months. (**a-c**) Body weight curve (**a**), body weight at the age of 20 weeks (**b**), liver weight (%) (**c**) of the mice. WT: *n*=7 (a-b) or *n*=9 (c); S33A: *n*=7 (a-b) or *n*=9 (c) biologically independent animals. (**d**) H&E staining and weight ratios of brown fat tissue (BAT), epidydimal fat (EF) and subcutaneous adipose tissue (SAT) in WT and S33A mice. WT: *n*=7; S33A: *n*=7 biologically independent animals. (**e**) Quantitative RT-PCR analysis showing the expression of genes involved in gluconeogenesis in the WT and S33A mice. WT: *n*=5; S33A: *n*=5 biologically independent animals. (**f-h**) Immunostaining of F4/80 and CD68, specific markers of macrophages in WT and S33A mice at the age of 5 months (**f**). Quantification data of F4/80^{+} cells (**g**) and CD68^{+} cells (**h**) were displayed. WT: *n*=5; S33A: *n*=5 biologically independent animals.

Data represent mean ± SEM; Significance was calculated by two-tailed student's *t* test (**b&c**), (**g&h**) or two-way ANOVA with Bonferroni post hoc analysis (**d&e**).

a

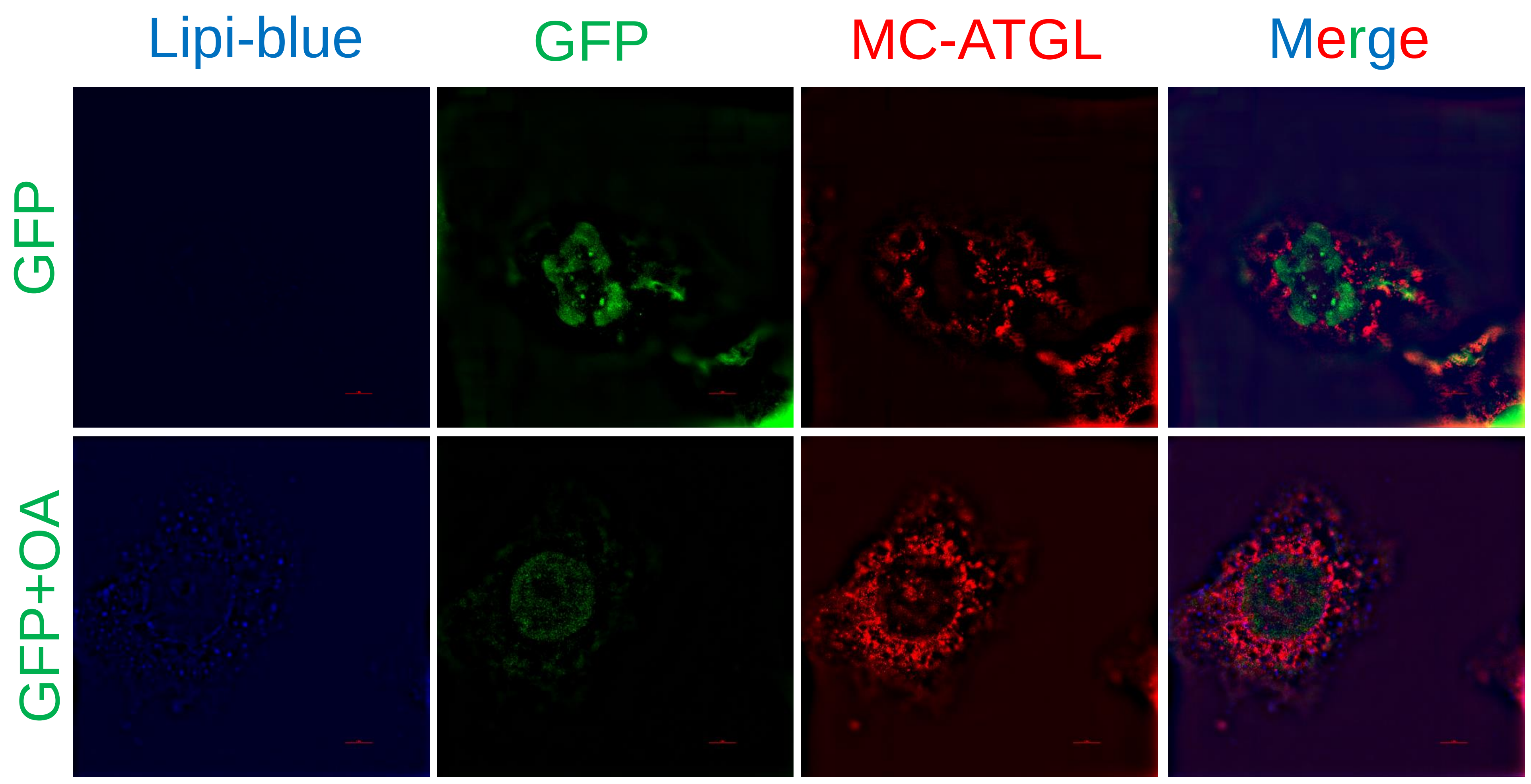

Figure S5. 17β-HSD13 directly binds to ATGL *in vitro*.
Huh7 cells were stably infected with GFP lentivirus together with MC-ATGL lentivirus. 150mm OA was loaded for 16h. The cells were fixed and LDs were stained with Lipi-blue, an LD tracker. Cytosolic ATGL was translocated on the LD surface after OA loading; scale bar=5 μm.

a

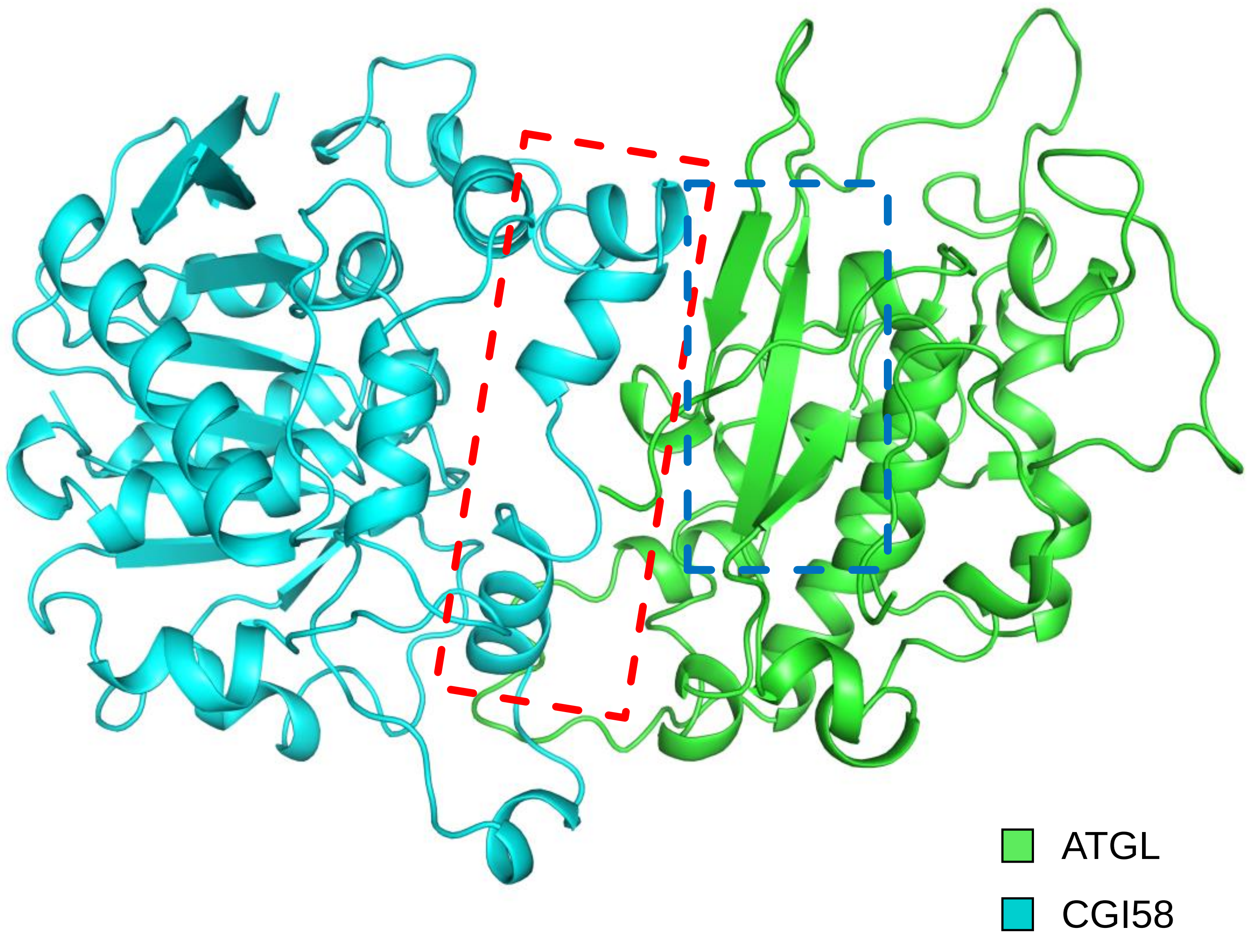

b

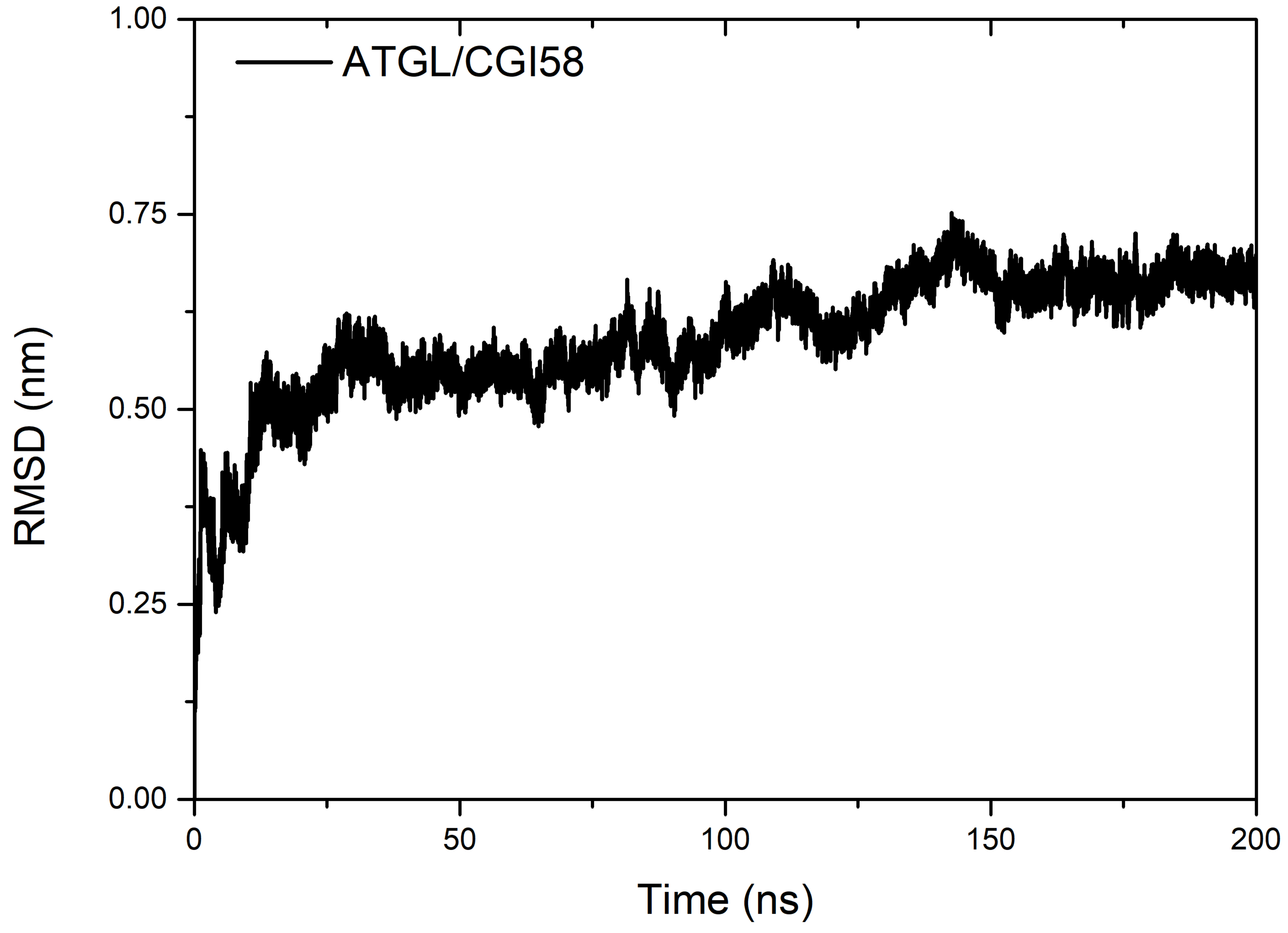

Figure S6. The protein docking analysis of ATGL and CGI-58 interaction.
(a) The structure of an ATGL-CGI58 dimer obtained from the protein-protein docking and the subsequent MD relaxation.
(**b**) The RMSD curve of the ATGL-CGI58 complex system along with the time trace from the MD simulation.

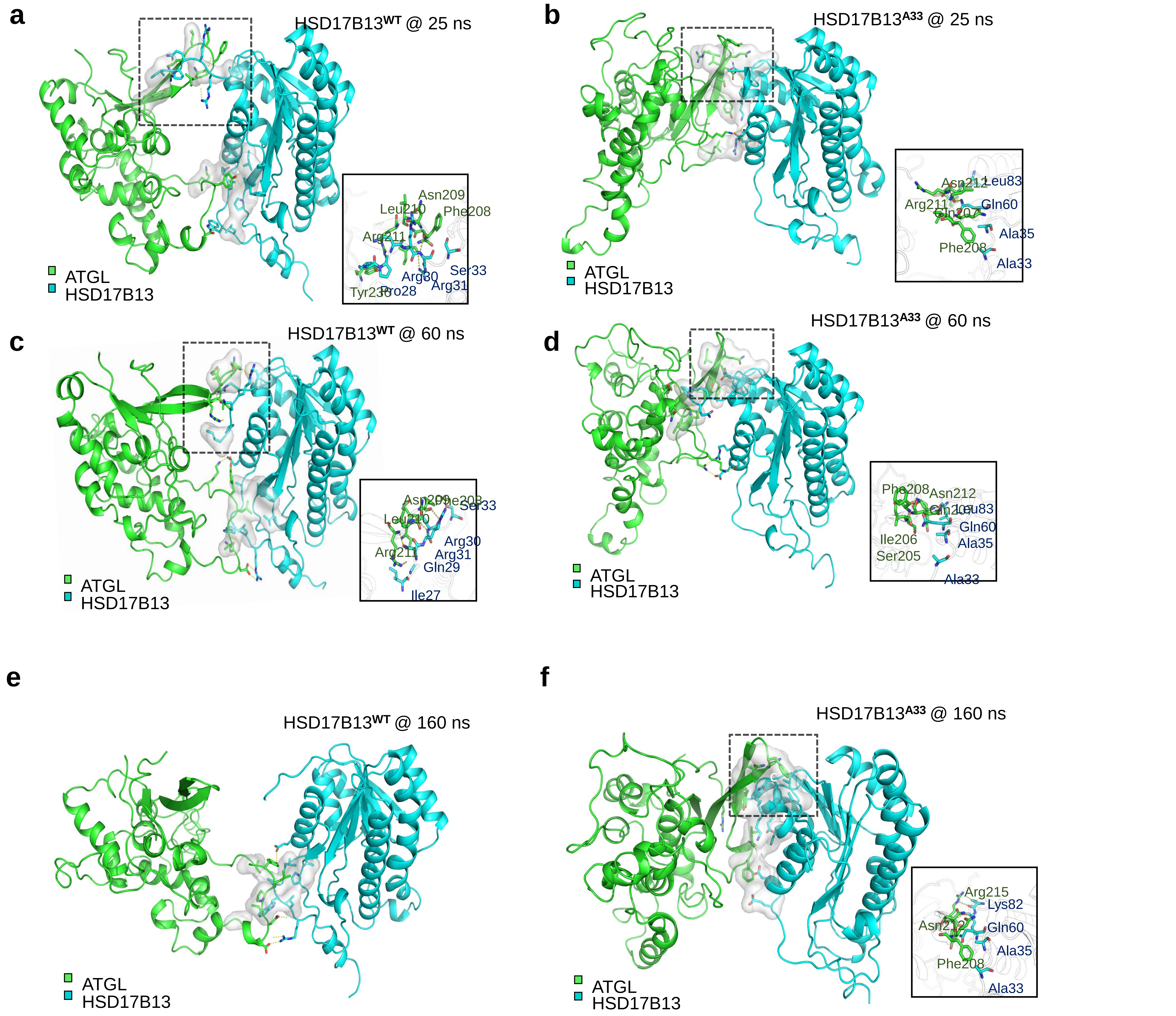

Figure S7. Possible interaction sites of ATGL with 17β-HSD13-WT and 17β-HSD13-S33A.
The snapshots of the MD simulation at 25ns of 17β-HSD13-WT-ATGL with the local structure around the S33 in 17β-HSD13-WT (**a**) or around the A33 in 17β-HSD13-S33A (**b**); The snapshots of the MD simulation at 60ns of 17β-HSD13-WT-ATGL with the local structure around the S33 in 17β-HSD13-WT (**c**) or around the A33 in 17β-HSD13-S33A (**d**); The snapshots of the MD simulation at 160ns of 17β-HSD13-WT-ATGL with the local structure around the S33 in 17β-HSD13-WT (**e**) or around the A33 in 17β-HSD13-S33A (**f**).

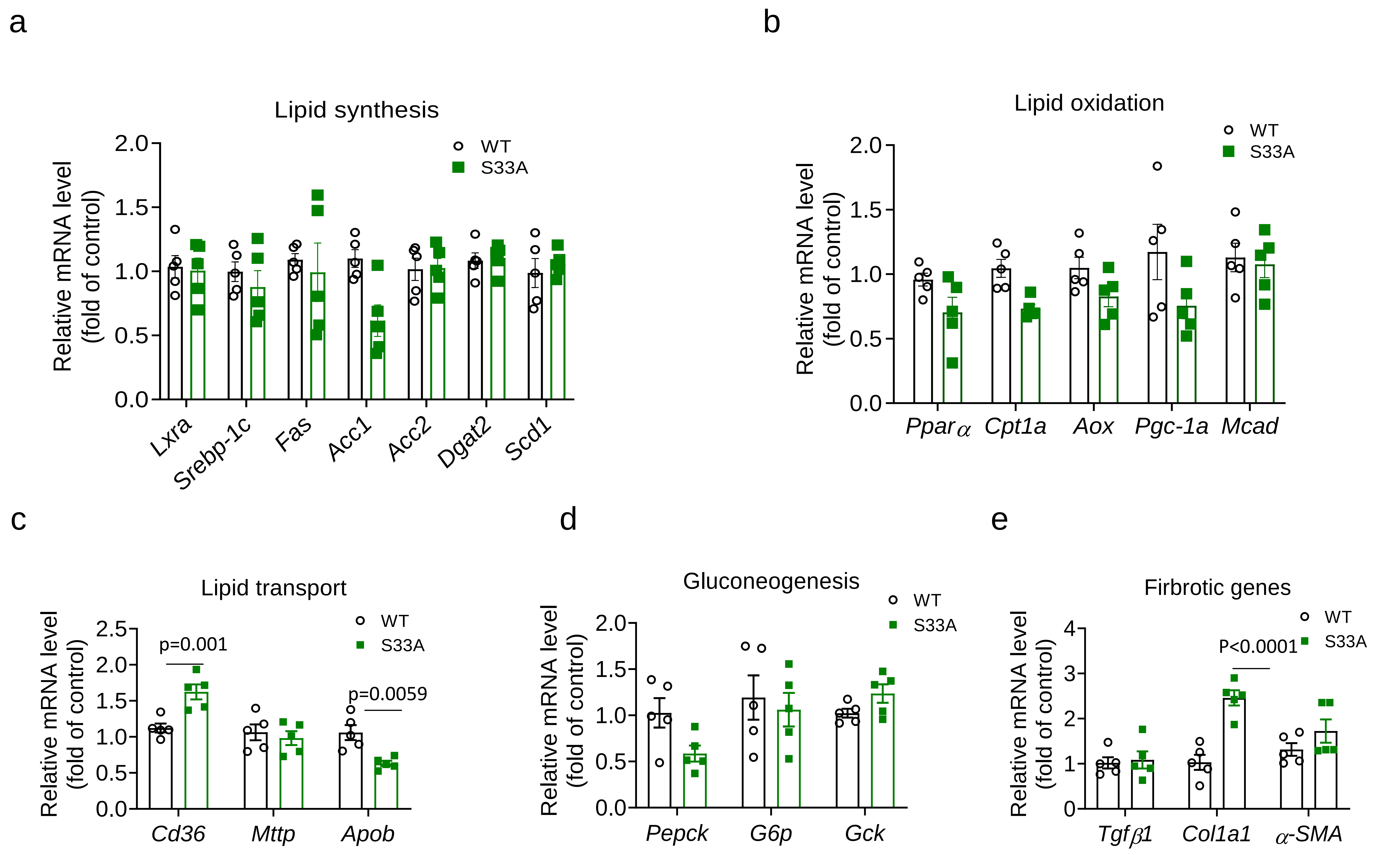

Figure S8. Expression of genes involved in lipid metabolism and transport and gluconeogenesis in the livers of the *Hsd17B13*$^{+/+}$ and *Hsd17b13*$^{33A/A}$ mice fed a high-fat diet.

Expression of genes related to lipid synthesis (**a**), lipid oxidation (**b**), lipid transport (**c**), gluconeogenesis (**d**), and fibrosis (**e**) in the livers of the *Hsd17b13*$^{+/+}$ (WT) and *Hsd17b13*$^{33A+/+}$ (S33A) mice fed with a high-fat diet (HFD) for 3 months was quantified by qPCR. WT: *n*=5; S33A: *n*=5 biologically independent animals. Data represent mean ± SEM; Significance was calculated by two-way ANOVA with Bonferroni post hoc analysis.

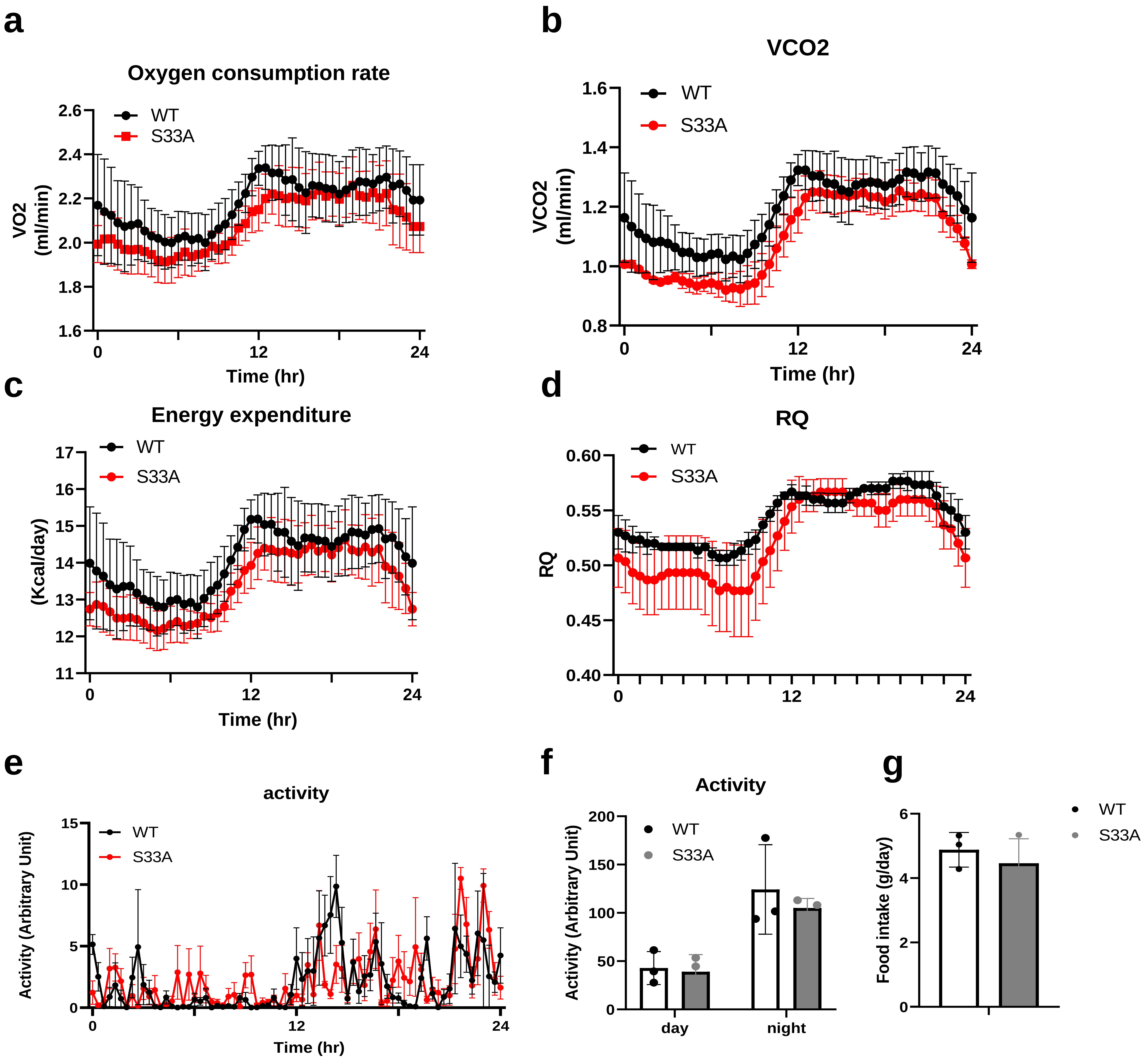

Figure S9. Whole-body energy metabolism in the *Hsd17b13*$^{+/+}$ and *Hsd17b13*$^{33A/A}$ mice fed an HFD. Metabolic cage studies were performed in the *Hsd17b13*$^{+/+}$ and *Hsd17b13*$^{33A/A}$ mice. O2 consumption (VO2) (**a**), CO2 production (VCO2) (**b**), energy expenditure (**c**), RQ (**d**), activity (**e, f**) and daily food intake (**g**) in the *Hsd17b13*$^{+/+}$ (WT) and *Hsd17b13*$^{33A+/+}$ (S33A) mice after feeding a high-fat diet for 3 months. WT: *n*=5; S33A: *n*=5 biologically independent animals. Data represent mean ± SEM; ANCOVA analysis was performed. No difference in EE was observed between two genotypes.

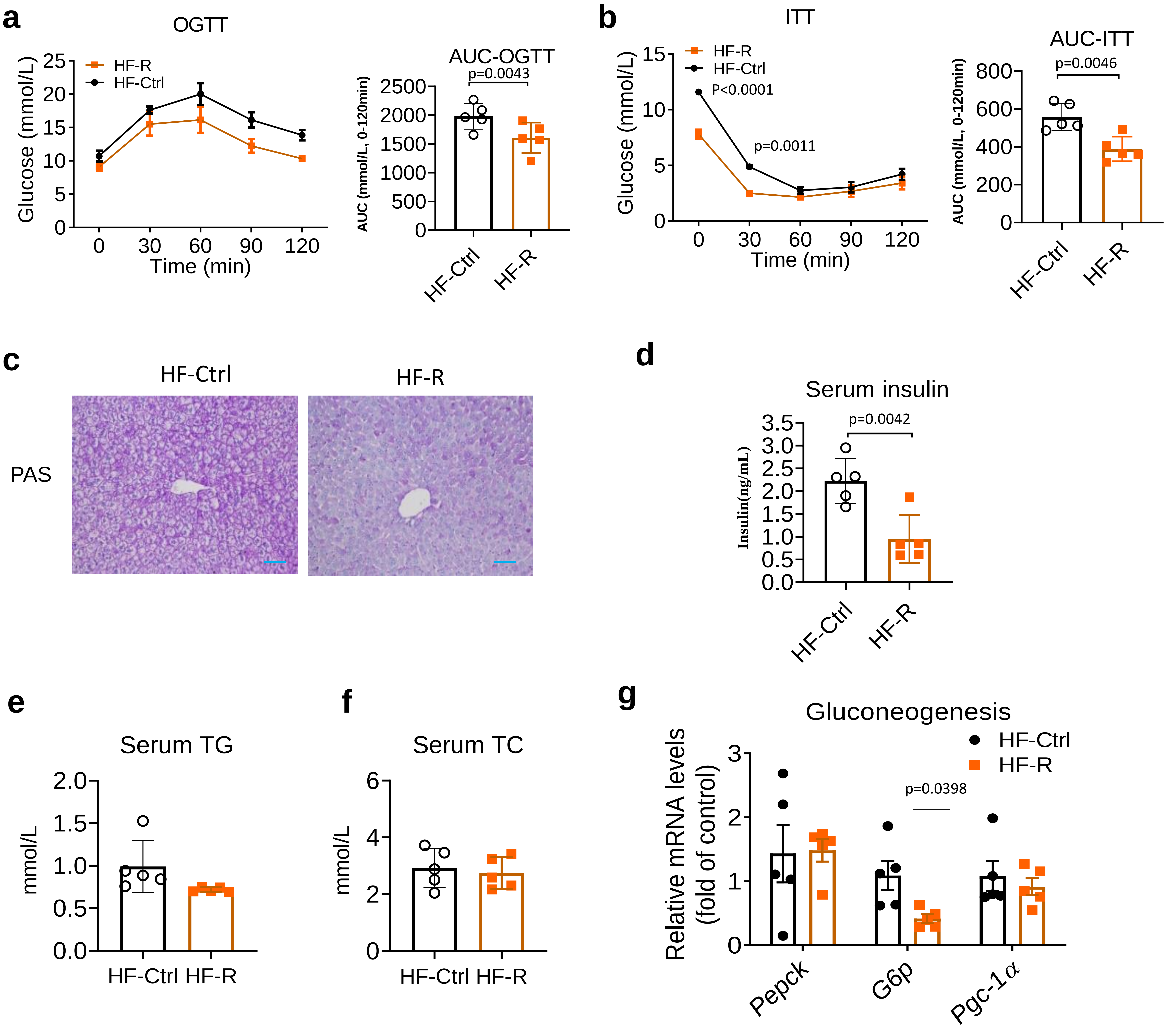

Figure S10. Reproterol ameliorates HFD-induced insulin resistance.

Male C57BL/6 mice at the age of 6 weeks were fed with a HFD for 16 weeks. Ten weeks after HFD treatment, mice began to receive reproterol treatment via intragastric administration at the dosage of 5mg/kg body weight daily for 6 weeks. HF-R, mice receiving both HFD and reproterol; HF-Ctrl, mice only receiving HFD. OGTT (**a**) and ITT (**b**) were performed after 1-month reproterol treatment. PAS staining (**c**) of HF-Ctrl and HF-R group (scale bar, 50 μm). Serum insulin (**d**), serum triglycerides (TG) (**e**) and cholesterol (TC) (**f**) levels were measured. Quantitative PCR assay showing the changes in mRNA expression levels of genes involved in gluconeogenesis (**g**).

HF-Ctrl: *n*=5; HF-R: *n*=5 biologically independent animals. Data represent mean ± SEM; Significance was calculated by two-tailed student's t test (**d-f**) or two-way ANOVA with Bonferroni post hoc analysis (**a-b**), (**g**).

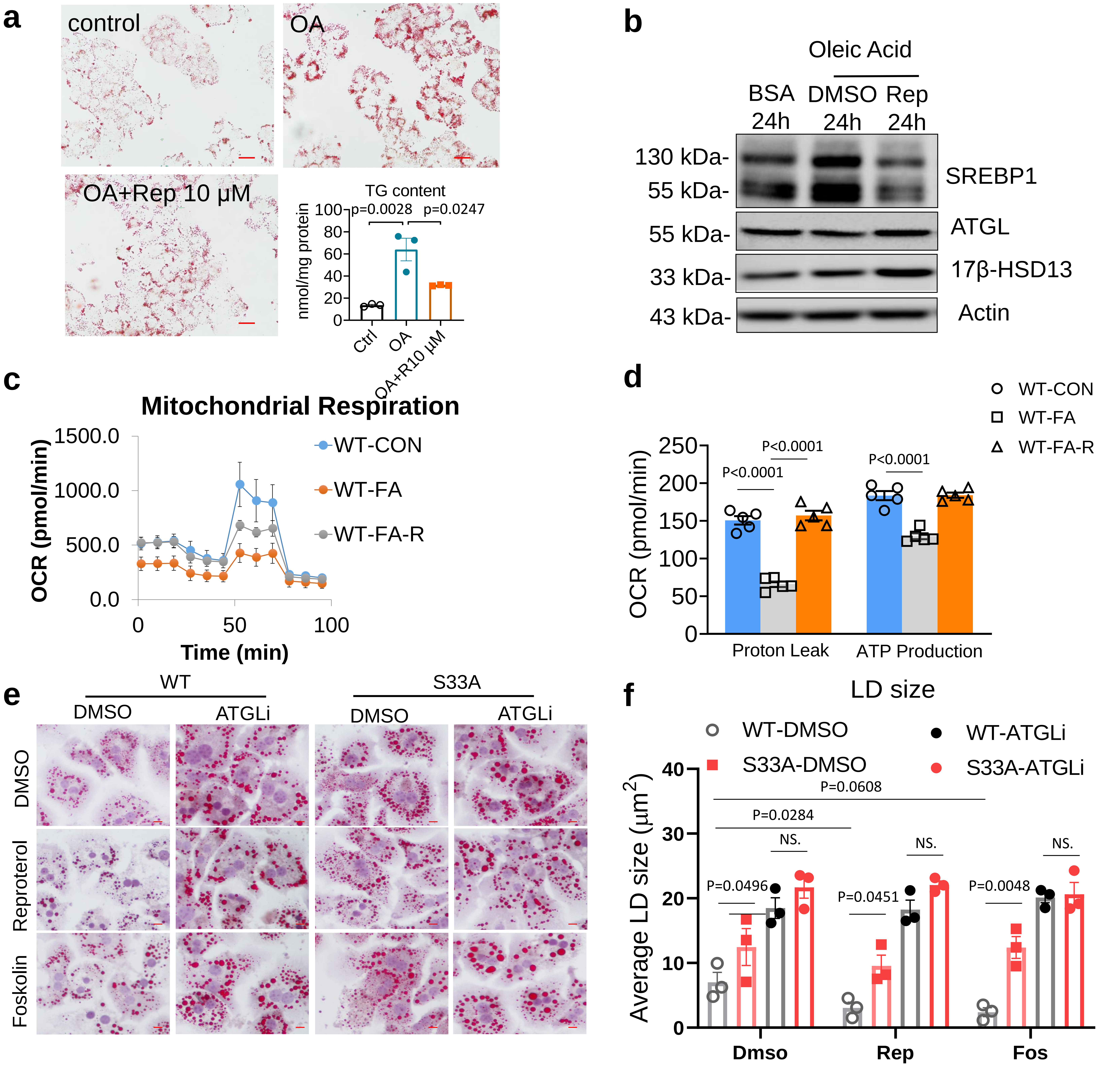

Figure S11. Reproterol activates hepatic lipolysis by phosphorylating 17β-HSD13 at Ser33 in a PKA-dependent manner.

(**a**) HepG2 cells were loaded with 200 μM oleic acid (OA) for 24h with or without reproterol at the concentration of 10 μm. Triglyceride (TG) contents were assessed in control (Ctrl), OA, and OA plus 10 μm reproterol (Rep). Ctrl: $n = 3$; OA: $n = 3$; OA+R10 μm: n=3 biologically independent cells. (**b**) Primary hepatocytes were cultured and treated with 10 μm reproterol for 24h. The protein expression of SREBP-1c, ATGL, 17β-HSD13, and β-actin were detected. BSA: $n = 3$; OA-DMSO: $n = 3$; OA-Rep: n=3 biologically independent cells.(**c-d**) Primary hepatocytes were seeded in 24 Seahorse plate and treated with palmitate acid (FA) plus DMSO or reproterol. The mitochondrial respiration (**c**) and OCR (**d**) were measured. WT-CON: $n = 3$; WT-FA: $n = 3$; WT-FA-R: n=4 biologically independent cells. (**e-f**) Primary hepatocytes from the WT and S33A mice were treated with 10 μm of reproterol or 10 μm forskolin for 24h and then processed to Oil Red O staining. Average LD size was analyzed (**f**).

Data presented are the mean ± SEM of 3 replicates. One-way ANOVA with Bonferroni post hoc analysis was performed. Significance was calculated by two-tailed student's t test (**a**) or two-way ANOVA with Bonferroni post hoc analysis (**d, f**).

Supplemental Table 1. Primer list for the mice experiments.

Gene	Forward primer sequence (5'-3')	Reverse primer sequence (5'-3')
Hsd17b13	CTCTGGCCACATTGTCACAG	TGCAACTTCTTCCGGCTCTA
Ppara	CACGATGCTGTCCTCCTTGAT	GCCAGGCCGATCTCCA
Cpt1a	ATCGTGGTGGTGGGTGTGATAT	ACGCCACTCACGATGTTCTTC
Aox	TGTCATTCCTACCAACTGTC	CCATCTTCTCAACTAACACTC
Pgc-1a	CGCCGTGTGATTTACGTTGG	GCTGTCTCCATCATCCCGC
Lcad	CCGCCCGATGTTCTCATTCT	CGCCATGTTTCTCTGCGATG
Mcad	GCTAGTGGAGCACCAAGGAG	CCAGGCTGCTCTCTGGTAAC
Lxrα	ATCGCCTTGCTGAAGACCTCTG	GATGGGGTTGATGAACTCCACC
Srebp-1c	GGGCAAGTACACAGGAGGAC	AGATCTCTGCCAGTGTTGCC
Fasn	GCCATGCCCAGAGGGTGGTT	AGGGTCGACCTGGTCCTCA
Acc1	TGGAGCTAAACCAGCACTCC	GCCAAACCATCCTGTAAGCC
Acc2	CCAGTCTTCCGTGCCTTTGTAC	CTCATCCCTCGCTCTGAACG
Dgat2	AGTGGCAATGCTATCATCATCGT	AAGGAATAAGTGGGAACCAGA
Scd1	CATGCGATCTATCCGTCGGT	CCTCCAGGCACTGGAACATAG
Tgfβ1	TGACGTCACTGGAGTTGTACGG	GGTTCATGTCATGGATGGTGC
Col1a1	CAATGCAATGAAGAACTGGACTGT	TCCTACATCTTCTGAGTTTGGTGA
α-sma	CTGACAGAGGCACCACTGAA	CATCTCCAGAGTCCAGCACA
Pepck	CTGAAGGTGTCCCCCTTGTC	GATCTTGCCCTTGTGTTCTGC
G6p	CTGTTTGGACAACGCCCGTAT	AGGTGACAGGGAACTGCTTTA
Gck	GAAGCACCGACTGTGACTGA	TGCCAGGATCTGCTCTACCT
Cd36	GTGCAAAACCCAGATGACGT	TCCAACAGACAGTGAAGGCT
Mttp	GAGCGGTCTGGATTTACAACG	GTAGGTAGTGACAGATGTGGCTTTTG
Apob	TCACCCCCGGGATCAAG	TCCAAGGACACAGAGGGCTTT
Fatp1	CGCTTTCTGCGTATCGTCTG	GATGCACGGGATCGTGTCT
Fatp4	ACTGTTCTCCAAGCTAGTGCT	GATGAAGACCCGGATGAAACG
Fatp5	CTACGCTGGCTGCATATAGATG	CCACAAAGGTCTCTGGAGGAT
β-actin	GAGAGGGAAATCGTGCGTGAC	CATCTGCTGGAAGGTGGACA

Fig S2a

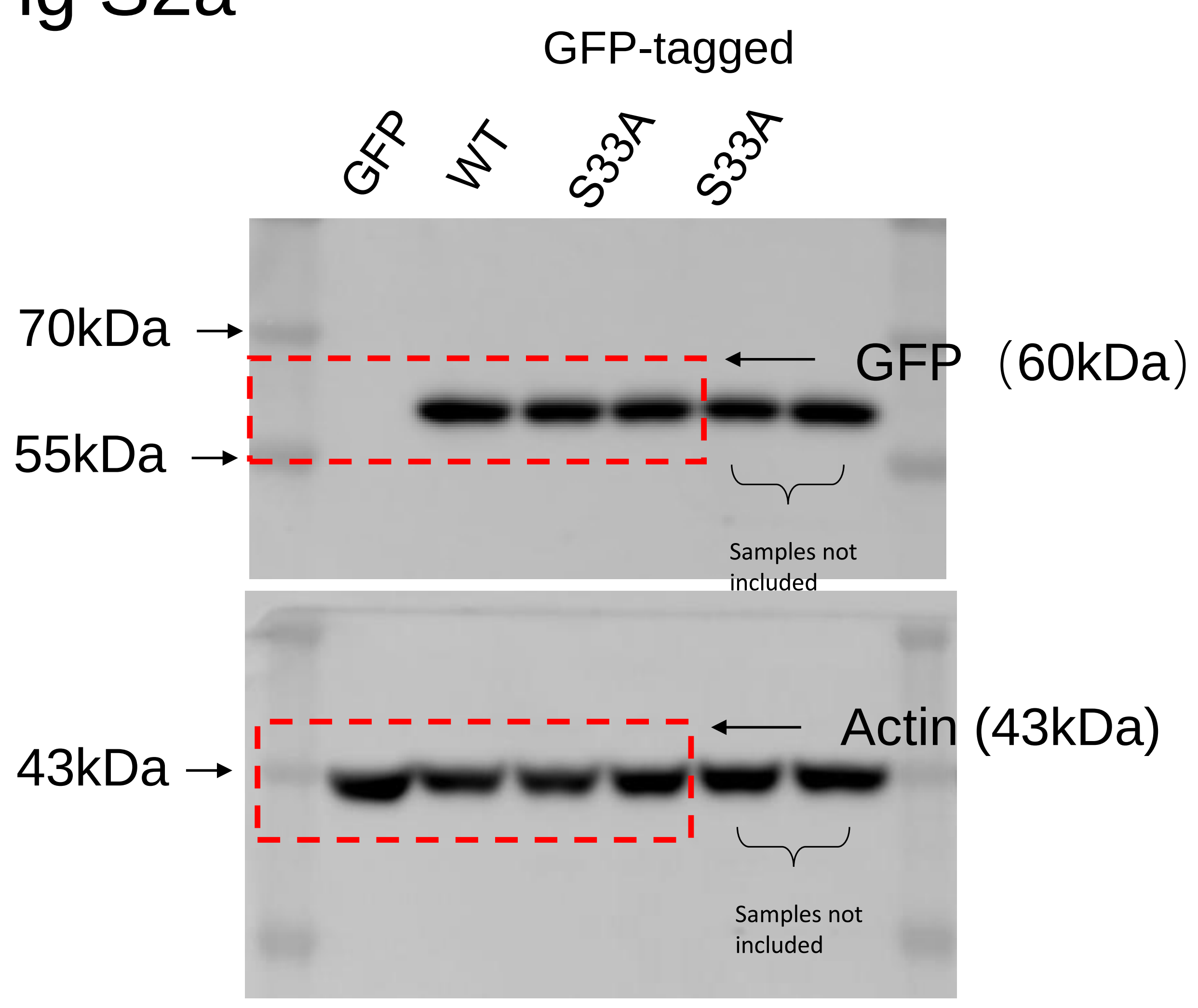

Fig S3e

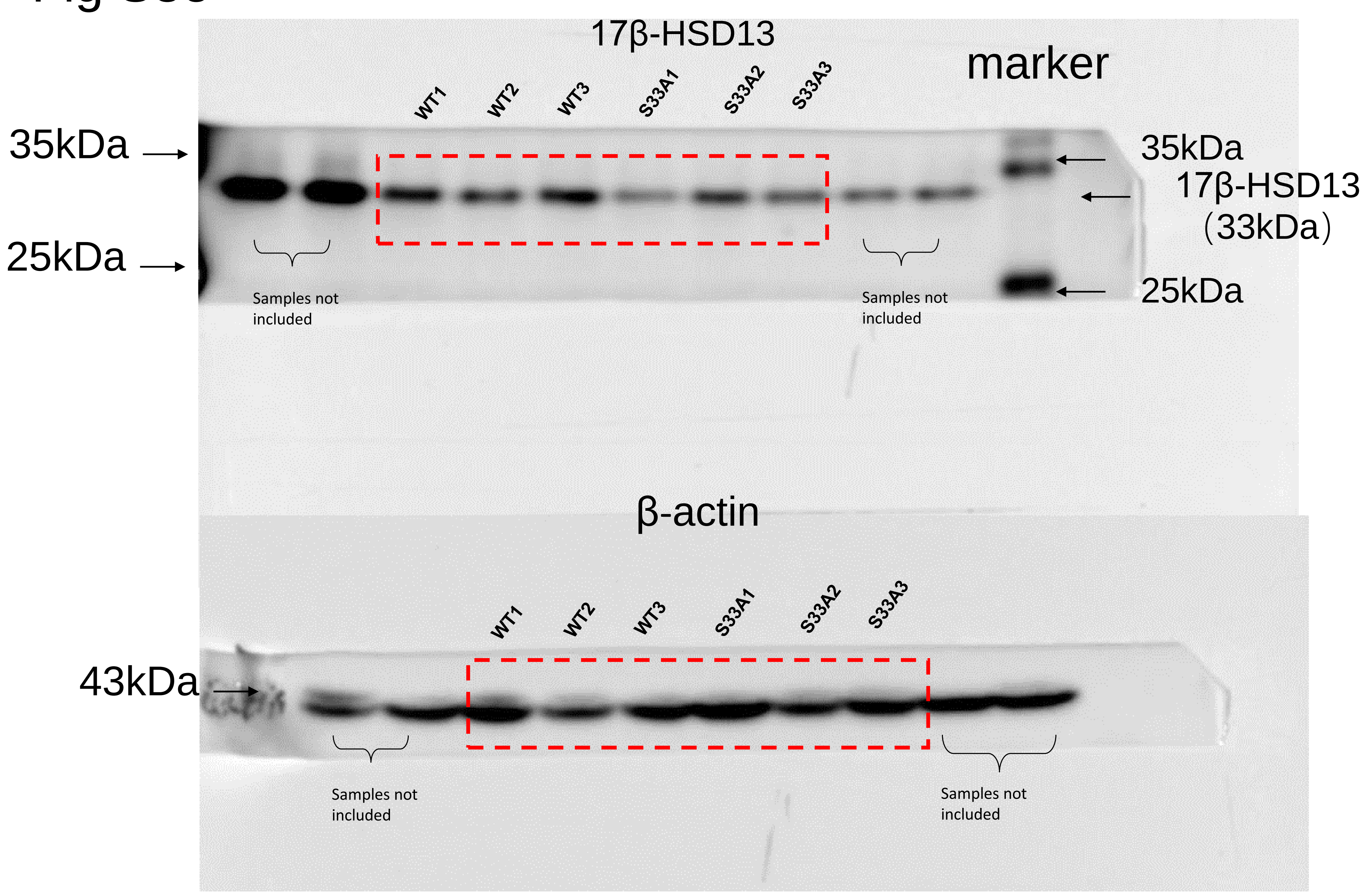

Fig S11b

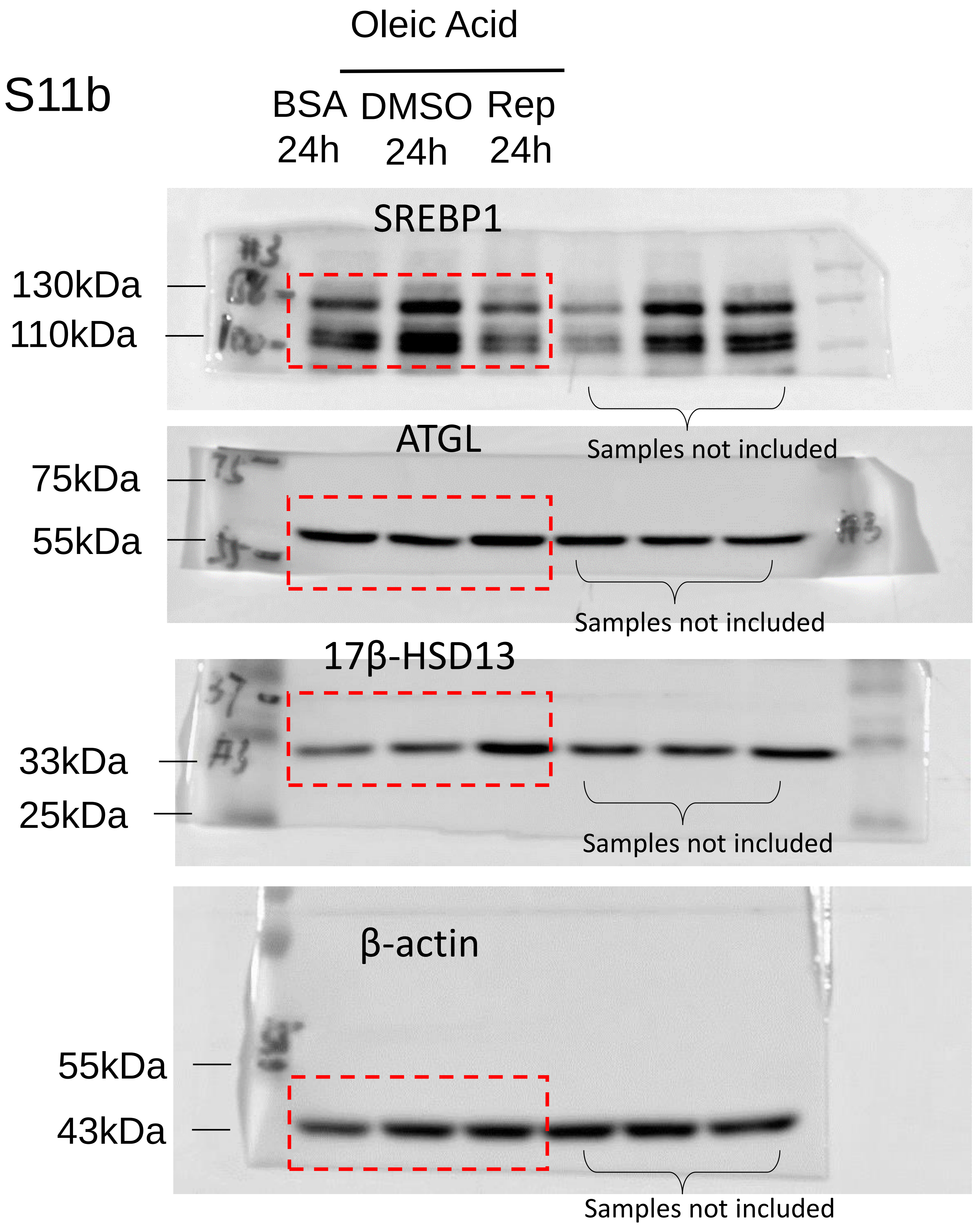

Supplement: Supplementary file 1 — Supplementary Information [file 41467_2022_34299_MOESM1_ESM.pdf]
