# Supplementary material for: Phosphorylation of 17β-hydroxysteroid dehydrogenase 13 at serine 33 attenuates nonalcoholic fatty liver disease in mice
Source: Nat Commun. 2022 Nov 2;13:6577. doi: 10.1038/s41467-022-34299-1 (PMC9630536; doi:10.1038/s41467-022-34299-1)
Supplement: Supplementary file 2 — Reporting Summary [file 41467_2022_34299_MOESM2_ESM.pdf]

nature portfolio

Corresponding author(s): Wen Su

Last updated by author(s): Oct 7, 2022

Reporting Summary

Nature Portfolio wishes to improve the reproducibility of the work that we publish. This form provides structure for consistency and transparency in reporting. For further information on Nature Portfolio policies, see our Editorial Policies and the Editorial Policy Checklist.

Statistics

For all statistical analyses, confirm that the following items are present in the figure legend, table legend, main text, or Methods section.

n/a	Confirmed	
☐	☒	The exact sample size (*n*) for each experimental group/condition, given as a discrete number and unit of measurement
☐	☒	A statement on whether measurements were taken from distinct samples or whether the same sample was measured repeatedly
☐	☒	The statistical test(s) used AND whether they are one- or two-sided<br>*Only common tests should be described solely by name; describe more complex techniques in the Methods section.*
☒	☐	A description of all covariates tested
☐	☒	A description of any assumptions or corrections, such as tests of normality and adjustment for multiple comparisons
☐	☒	A full description of the statistical parameters including central tendency (e.g. means) or other basic estimates (e.g. regression coefficient) AND variation (e.g. standard deviation) or associated estimates of uncertainty (e.g. confidence intervals)
☐	☒	For null hypothesis testing, the test statistic (e.g. *F*, *t*, *r*) with confidence intervals, effect sizes, degrees of freedom and *P* value noted<br>*Give P values as exact values whenever suitable.*
☒	☐	For Bayesian analysis, information on the choice of priors and Markov chain Monte Carlo settings
☒	☐	For hierarchical and complex designs, identification of the appropriate level for tests and full reporting of outcomes
☒	☐	Estimates of effect sizes (e.g. Cohen's *d*, Pearson's *r*), indicating how they were calculated

Our web collection on statistics for biologists contains articles on many of the points above.

Software and code

Policy information about availability of computer code

Data collection	Confocal microscopy: Zeiss LSM880; Nikon-N-SIM; RT-PCR: Thermo quant studio 5; Western blot: GE, E-blot (Beijing Kaichuang, China)
Data analysis	The following software were used in this study: Excel (2007), GraphPad Prism (V. 8.4) , Image J 1.48v, LipidSearch Software 4.0, has been used for group analyses.

For manuscripts utilizing custom algorithms or software that are central to the research but not yet described in published literature, software must be made available to editors and reviewers. We strongly encourage code deposition in a community repository (e.g. GitHub). See the Nature Portfolio guidelines for submitting code & software for further information.

Data

Policy information about availability of data

All manuscripts must include a data availability statement. This statement should provide the following information, where applicable:

- Accession codes, unique identifiers, or web links for publicly available datasets
- A description of any restrictions on data availability
- For clinical datasets or third party data, please ensure that the statement adheres to our policy

All data generated or analyzed during this study are included in this published article. The phosphorylation MS data has been submitted to PRIDE (1-20221006-13165). The lipidomic data generated in the study have been deposited in the Metabolights under accession code MTBLS6094. The other source data are provided as a Source Data file.

Human research participants

Policy information about studies involving human research participants and Sex and Gender in Research.

Reporting on sex and gender	n/a
Population characteristics	n/a
Recruitment	n/a
Ethics oversight	n/a

Note that full information on the approval of the study protocol must also be provided in the manuscript.

Field-specific reporting

Please select the one below that is the best fit for your research. If you are not sure, read the appropriate sections before making your selection.

- [x] Life sciences
- [ ] Behavioural & social sciences
- [ ] Ecological, evolutionary & environmental sciences

For a reference copy of the document with all sections, see nature.com/documents/nr-reporting-summary-flat.pdf

Life sciences study design

All studies must disclose on these points even when the disclosure is negative.

Sample size	No sample size was performed. Sample sizes were chosen to satisfy statistical power based on previous experience and knowledge (PMID:32779242). At least 5 mice/group was used in this study. For normal diet experiment, 10 HSD17B13-S33A mutant mice and 9 HSD17B13-wild type littermate WT controls were used. For high fat diet experiment 10 HSD17B13-s33a mice and 9 wild type littermates were used. (The sample size increased to meet the reviewer's concern about the statistical power.) For the AAV- overexpression experiment and Reproterol treatment experiment, 5 male mice (6-8 weeks) were used.
Data exclusions	No data were excluded
Replication	All replication were successful and are biological replicates. For IP experiments, at least 2 independently experiments were performed. For other experiments related to statistical test, at least 3 biological independent experiments were performed. The detailed information was provided in corresponding figure legends.
Randomization	samples were allocated randomly
Blinding	The investigators were blinded to group allocation during data collection and analysis

Reporting for specific materials, systems and methods

We require information from authors about some types of materials, experimental systems and methods used in many studies. Here, indicate whether each material, system or method listed is relevant to your study. If you are not sure if a list item applies to your research, read the appropriate section before selecting a response.

Materials & experimental systems

n/a	Involved in the study	
[ ]	[x]	Antibodies
[ ]	[x]	Eukaryotic cell lines
[x]	[ ]	Palaeontology and archaeology
[ ]	[x]	Animals and other organisms
[x]	[ ]	Clinical data
[x]	[ ]	Dual use research of concern

Methods

n/a	Involved in the study	
[x]	[ ]	ChIP-seq
[x]	[ ]	Flow cytometry
[x]	[ ]	MRI-based neuroimaging

Antibodies

Antibodies used	For the antibodies, phospho-PKA substrate antibody (catalog no. 9624, 1:1000 dilution), phospho-HSL (Ser-660) antibody (catalog no. 4126, 1:1000 dilution), myc (catalog no. 2276, 1:2000 dilution) and ATGL antibody (catalog no. 2138, 1:1000 dilution) were from

Cell Signaling Technology. The phospho-ATGL (Ser-406) antibody (catalog no. 135093, 1:1000 dilution), ADRP (catalog no. ab181452, 1:2000 dilution), PNPLA3 (catalog no.ab81874, 1:1000 dilution), CGI-58 (catalog no. ab183739, 1:1000 dilution), β-actin (catalog no. ab8226, 1:2000 dilution), F4/80 (catalog no. ab6640, 1:400 dilution for IHC staining), α-SMA (catalog no. ab5694, 1:400 dilution for IHC staining) and CD68 (catalog no. ab125212, 1:400 dilution for IHC staining) were from Abcam. GFP (catalog no. GTX113617, 1:2000 dilution), mCherry (catalog no. GTX128508, 1:2000 dilution) were from Genetex, 17β-HSD13 (OAAN01691, 1:1000 dilution) was from Aviva. The HRP conjugated secondary antibodies goat anti mouse IgG (ZB-2305, 1:10000 dilution) and goat anti mouse IgG (ZB-2301, 1:4000 dilution) were from ZSGB-BIO.

Validation

All antibodies were used per commercial sources and chosen based upon available validation studies. Available on the manufacturers' websites:
Anti-phospho-PKA substrate: (https://www.cellsignal.cn/products/primary-antibodies/phospho-pka-substrate-rrxs-t-100g7e-rabbit-mab/9624?site-search-type=Products&N=4294956287&Ntt=9624&fromPage=plp&_requestid=312236)
Anti-phospho-HSL (Ser-660): (https://www.cellsignal.cn/products/primary-antibodies/phospho-hsl-ser660-antibody/4126?site-search-type=Products&N=4294956287&Ntt=4126&fromPage=plp&_requestid=312477)
Anti -Myc: (https://www.cellsignal.cn/products/primary-antibodies/myc-tag-9b11-mouse-mab/2276?site-search-type=Products&N=4294956287&Ntt=2276&fromPage=plp)
Anti-ATGL: (https://www.cellsignal.cn/products/primary-antibodies/atgl-antibody/2138?site-search-type=Products&N=4294956287&Ntt=2138&fromPage=plp&_requestid=312772.)
Anti-phospho-ATGL: (Ser-406): (https://www.abcam.cn/adipose-triglyceride-lipase-phospho-s406-antibody-ab135093.html),
Anti-ADRP: (https://www.abcam.cn/adfp-antibody-2c5h8-ab181452.html),
Anti-PNPLA3: (https://www.abcam.cn/pnpla3-antibody-ab81874.html),
Anti-CGI-58 : (https://www.abcam.cn/nav/primary-antibodies/rabbit-monoclonal-antibodies/abhd5cgi-58-antibody-epr12621-ab183739.html),
Anti-F4/80: (https://www.abcam.cn/f480-antibody-cia3-1-macrophage-marker-ab6640.html),
Anti-α-SMA: (https://www.abcam.cn/alpha-smooth-muscle-actin-antibody-ab5694.html),
Anti-CD68 : (https://www.abcam.cn/cd68-antibody-ab125212.html),
Anti-β-actin: (https://www.abcam.cn/beta-actin-antibody-mabcam-8226-loading-control-ab8226.html).
Anti-GFP: (https://www.genetex.cn/Product/Detail/GFP-antibody/GTX113617),
Anti-mCherry : (https://www.genetex.cn/Product/Detail/mCherry-antibody/GTX128508),
Anti-17β-HSD13: (https://www.avivasysbio.com/hsd17b13-antibody-oaan01691.html),
Besides, antibody against HSD17B13 were also validated for immunoblotting in our lab by using KO mice.

Eukaryotic cell lines

Policy information about cell lines and Sex and Gender in Research

Cell line source(s)	The 293T, HepG2 and Huh-7 cells were purchased from the Cell Bank of Chinese Academy of Sciences (Shanghai, China)
Authentication	The cell lines were authenticated using STR method by the suppliers.
Mycoplasma contamination	All cell lines tested negative for mycoplasma contamination
Commonly misidentified lines (See ICLAC register)	No commonly misidentified lines were used

Animals and other research organisms

Policy information about studies involving animals; ARRIVE guidelines recommended for reporting animal research, and Sex and Gender in Research

Laboratory animals	17β-HSD13-S33A mutant mice (Hsd17b1333A/A) of C57BL/6J background were generated by CRISPR/Cas-mediated genome engineering (Cyagen Biosciences). Global Hsd17b13 gene deficient (hsd17b13-/-) mice of C57BL/6J background were constructed using the embryo micro-injection method (GemPharmatech, China). 8-week-old were obtained from Cyagen Biosciences Inc. All mice were maintained in a controlled environment of 20–23 °C, 50-60% humidity, with a 12/12 h light/dark cycle, and permitted ad libitum consumption of water and diet.
Wild animals	No wild animals were used in this study.
Reporting on sex	Male mice were used in the study. Previous study have reported that HSD17B13 is not involved in the metabolism of sex steroids and the regulation of reproductive functions.
Field-collected samples	The study did not involve samples collected from the field.
Ethics oversight	All animals received humane care in accordance with the guidelines established and approved by the Animal Experimentation Committee of Shenzhen University Health Science Center.

Note that full information on the approval of the study protocol must also be provided in the manuscript.
