# Supplementary material for: Phosphorylation of 17β-hydroxysteroid dehydrogenase 13 at serine 33 attenuates nonalcoholic fatty liver disease in mice
Source: Nat Commun. 2022 Nov 2;13:6577. doi: 10.1038/s41467-022-34299-1 (PMC9630536; doi:10.1038/s41467-022-34299-1)
Supplement: Supplementary file 3 — Source Data [file 41467_2022_34299_MOESM3_ESM.zip › Source data/Source Data 1.pdf]

Fig 1d

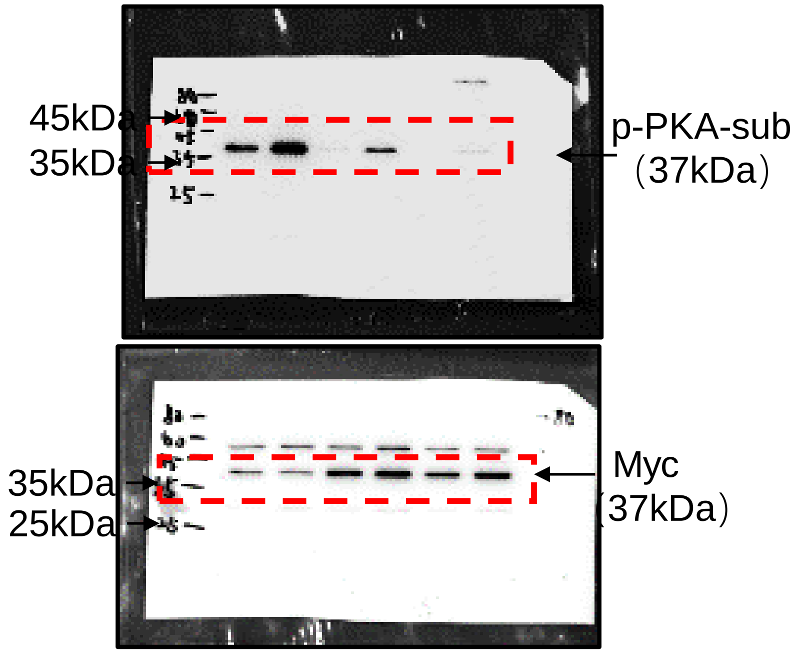

Fig 1e

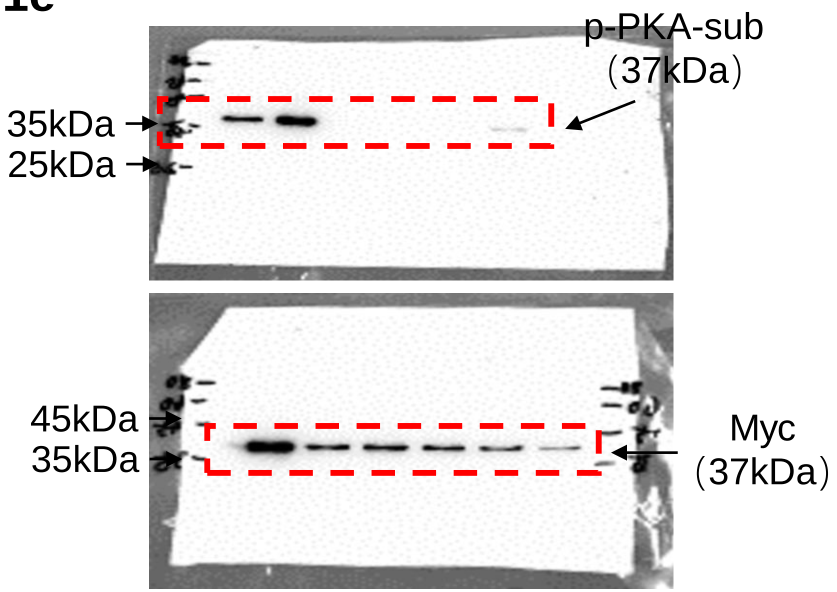

Fig 1f

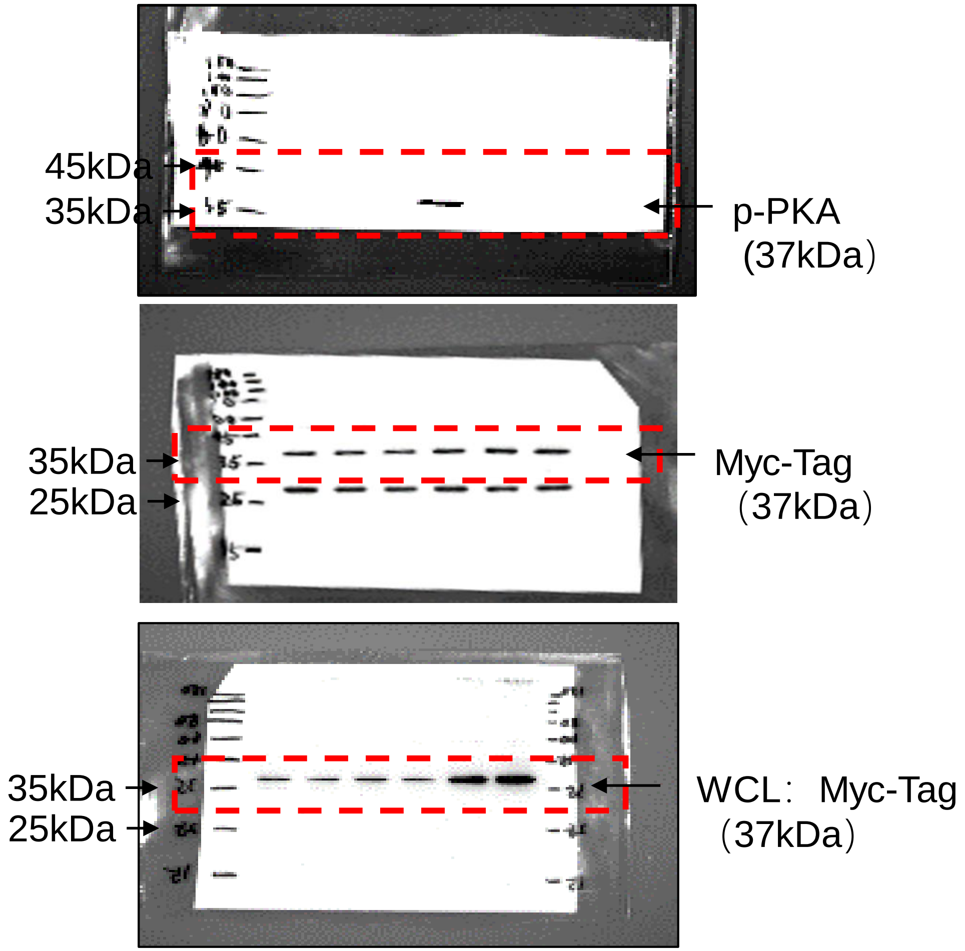

Fig 1g

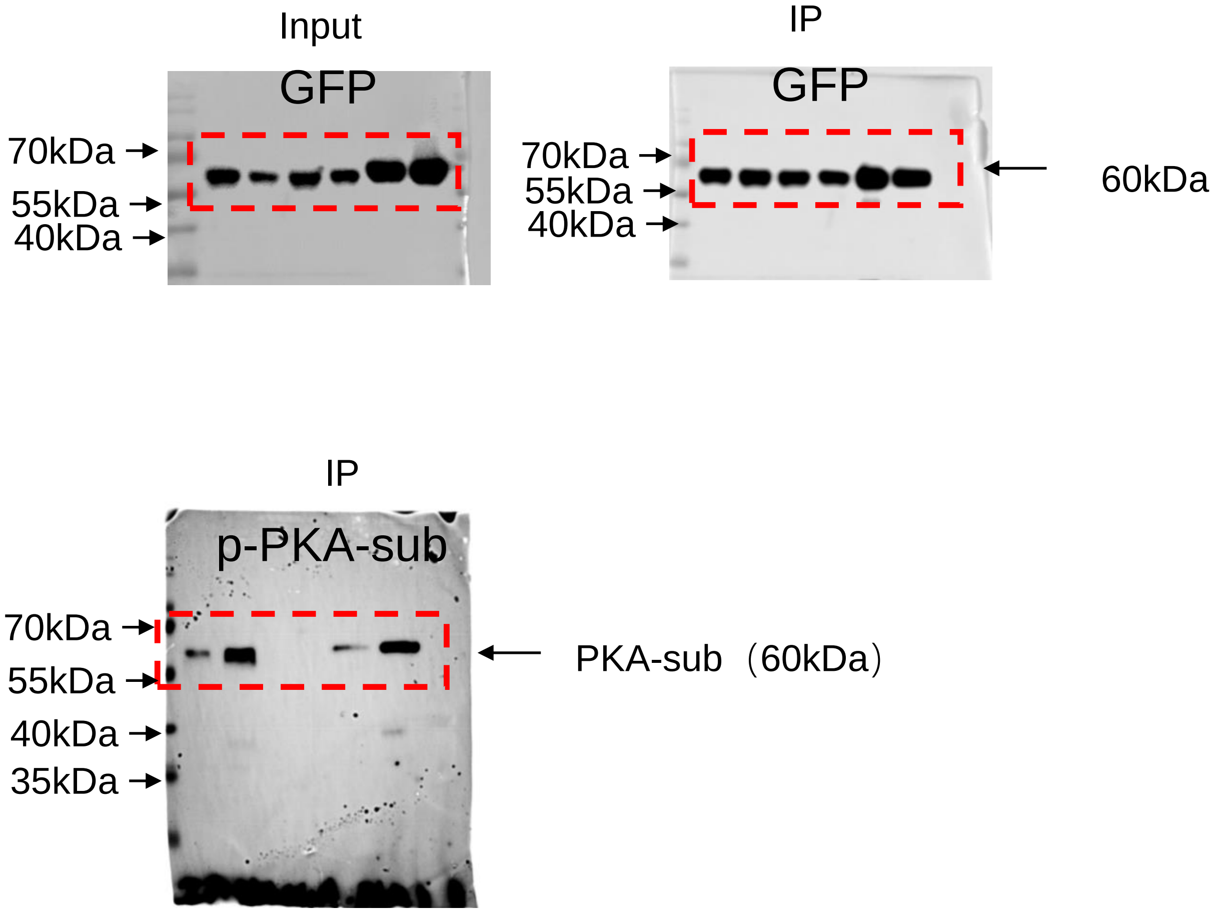
Input
GFP
IP
GFP
70kDa
55kDa
40kDa
70kDa
55kDa
40kDa
60kDa
IP
p-PKA-sub
70kDa
55kDa
40kDa
35kDa
PKA-sub（60kDa）

Fig 1h

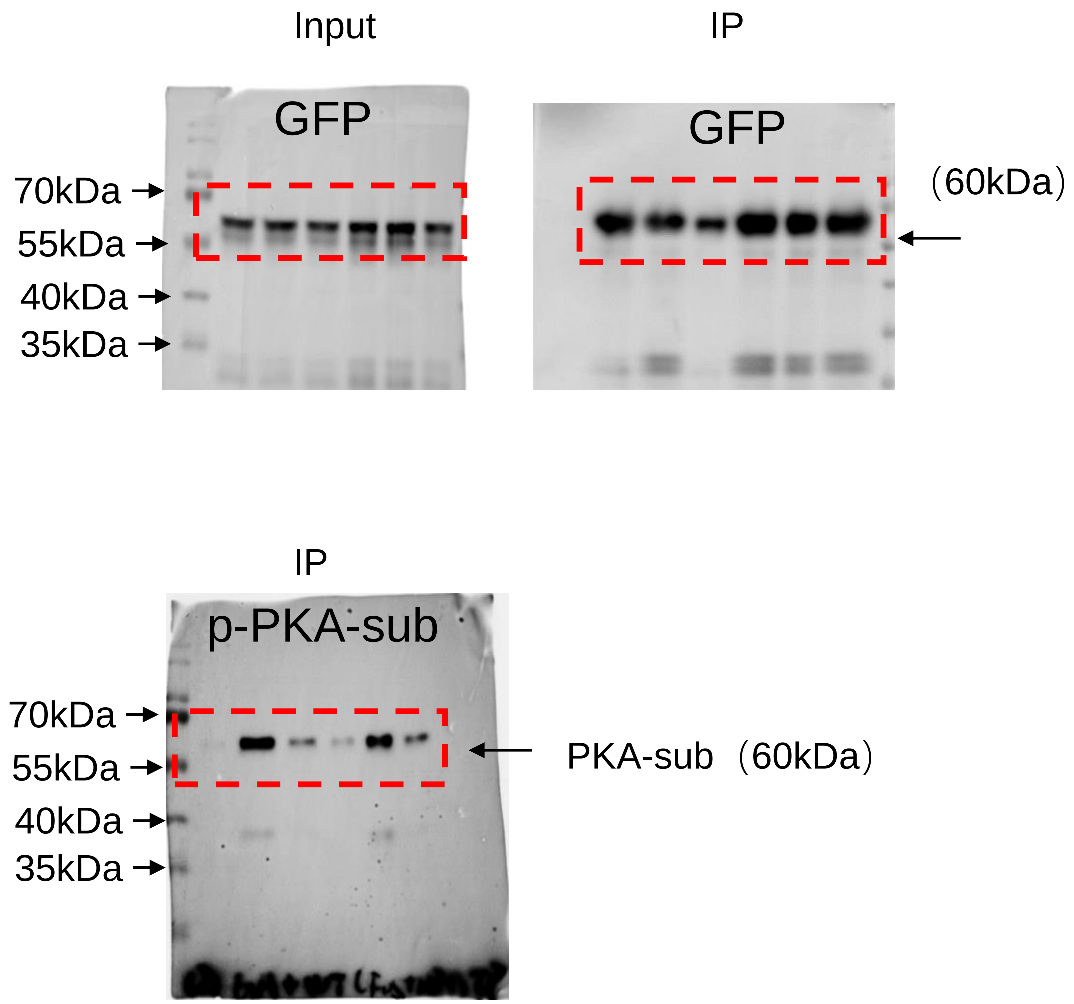
Input
IP
GFP
GFP
70kDa
55kDa
40kDa
35kDa
（60kDa）
IP
p-PKA-sub
70kDa
55kDa
40kDa
35kDa
PKA-sub（60kDa）

Fig 4g

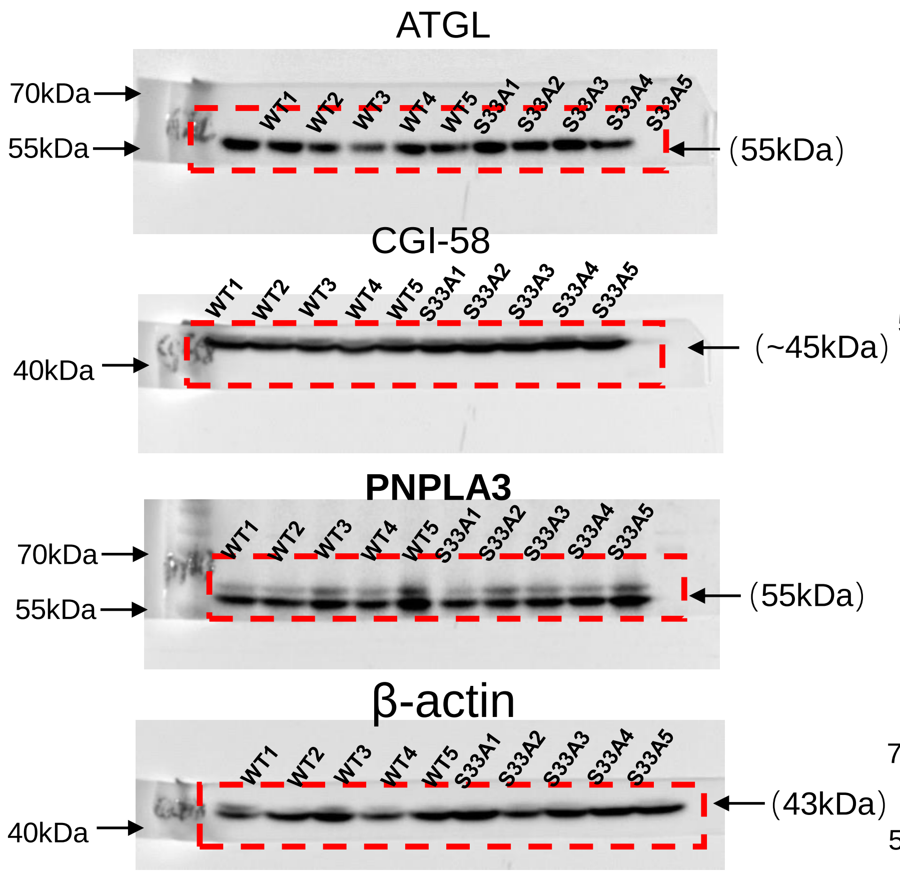

Fig 4h

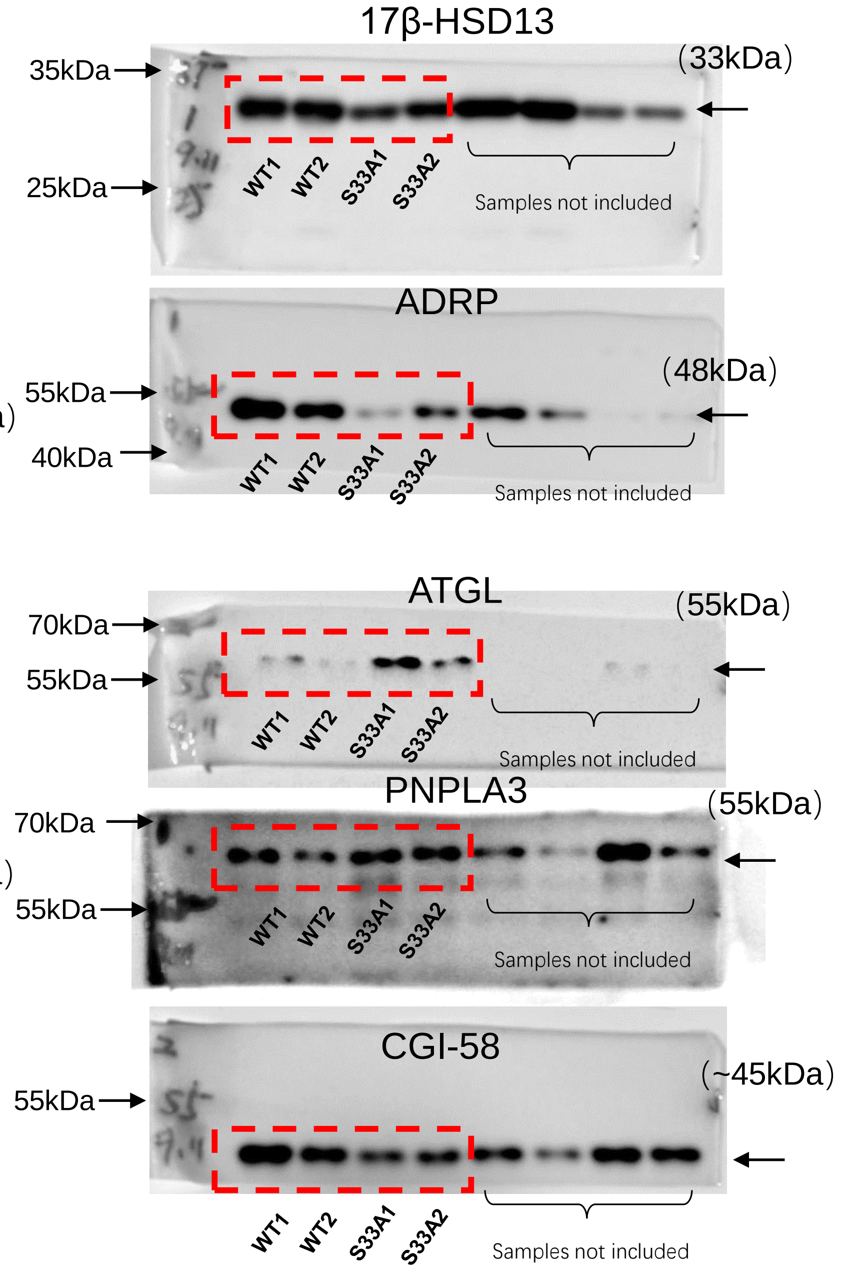

Silver stain

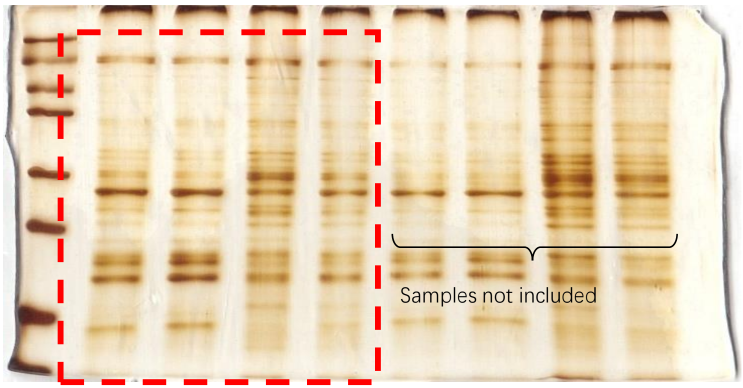

Fig 4i

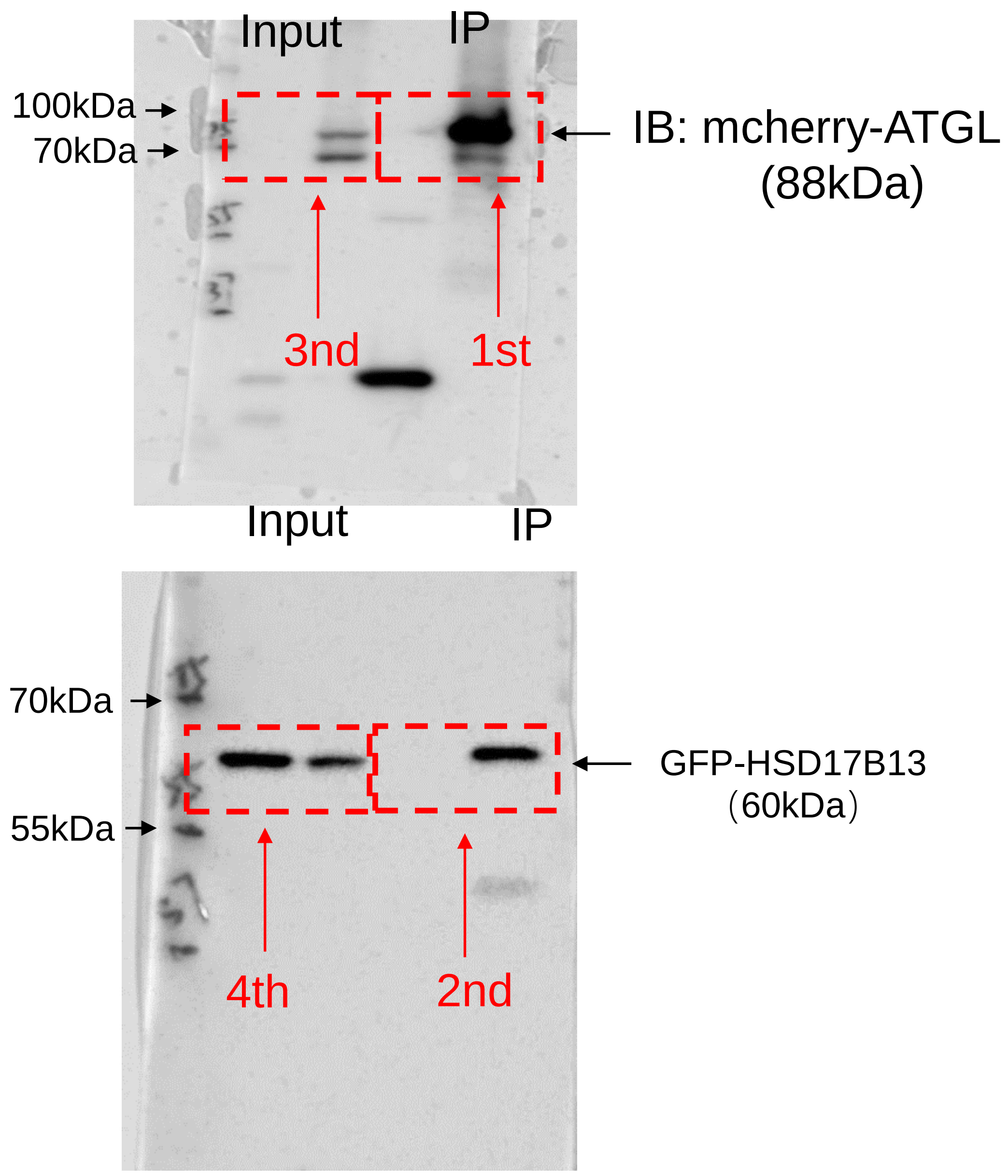
Input
IP
100kDa
70kDa
IB: mcherry-ATGL
(88kDa)
3nd
1st
Input
IP
70kDa
55kDa
GFP-HSD17B13
(60kDa)
4th
2nd

Fig 4j

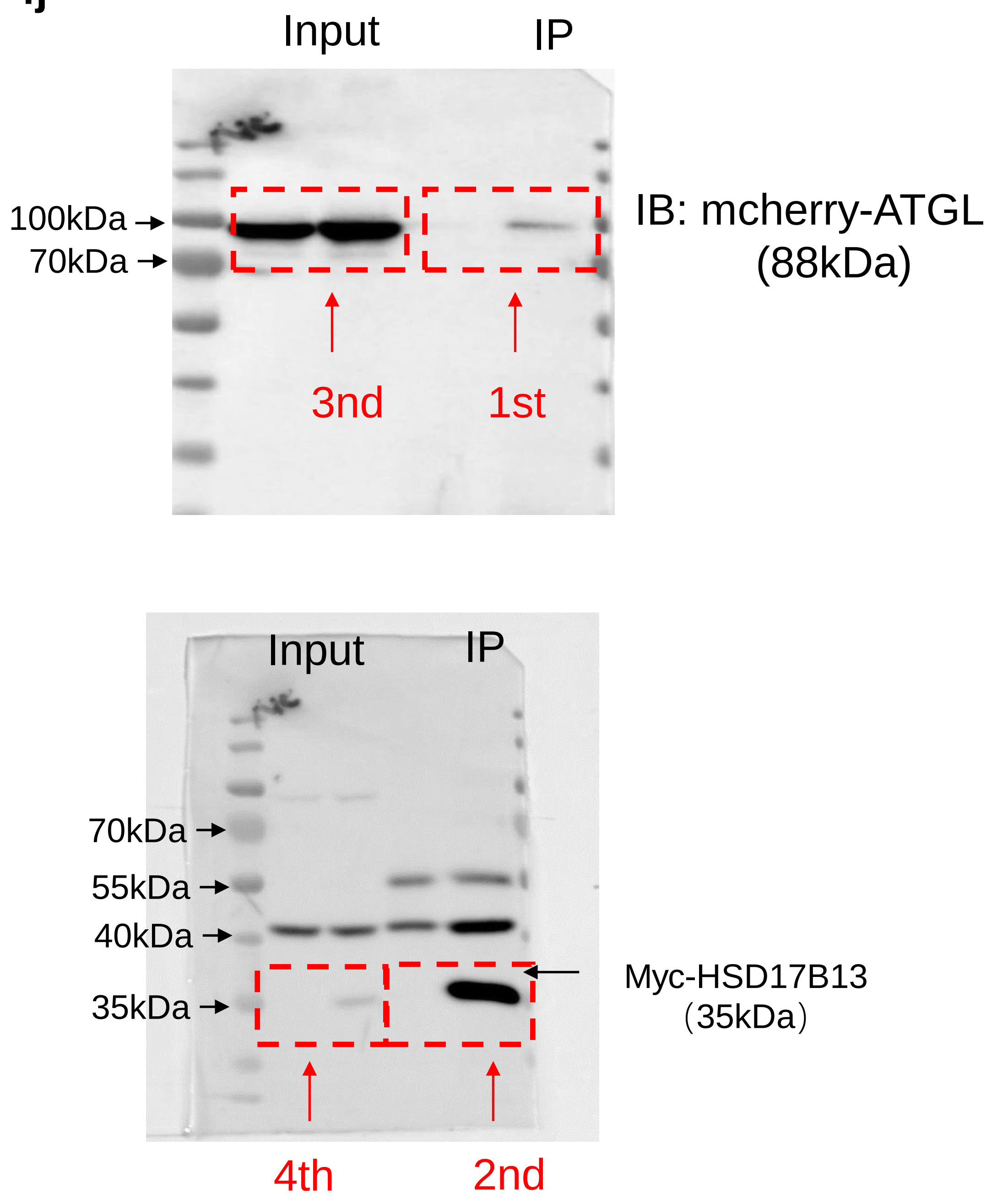
Input
IP
100kDa
70kDa
IB: mcherry-ATGL
(88kDa)
3nd
1st
Input
IP
70kDa
55kDa
40kDa
35kDa
Myc-HSD17B13
(35kDa)
4th
2nd

Fig 4k

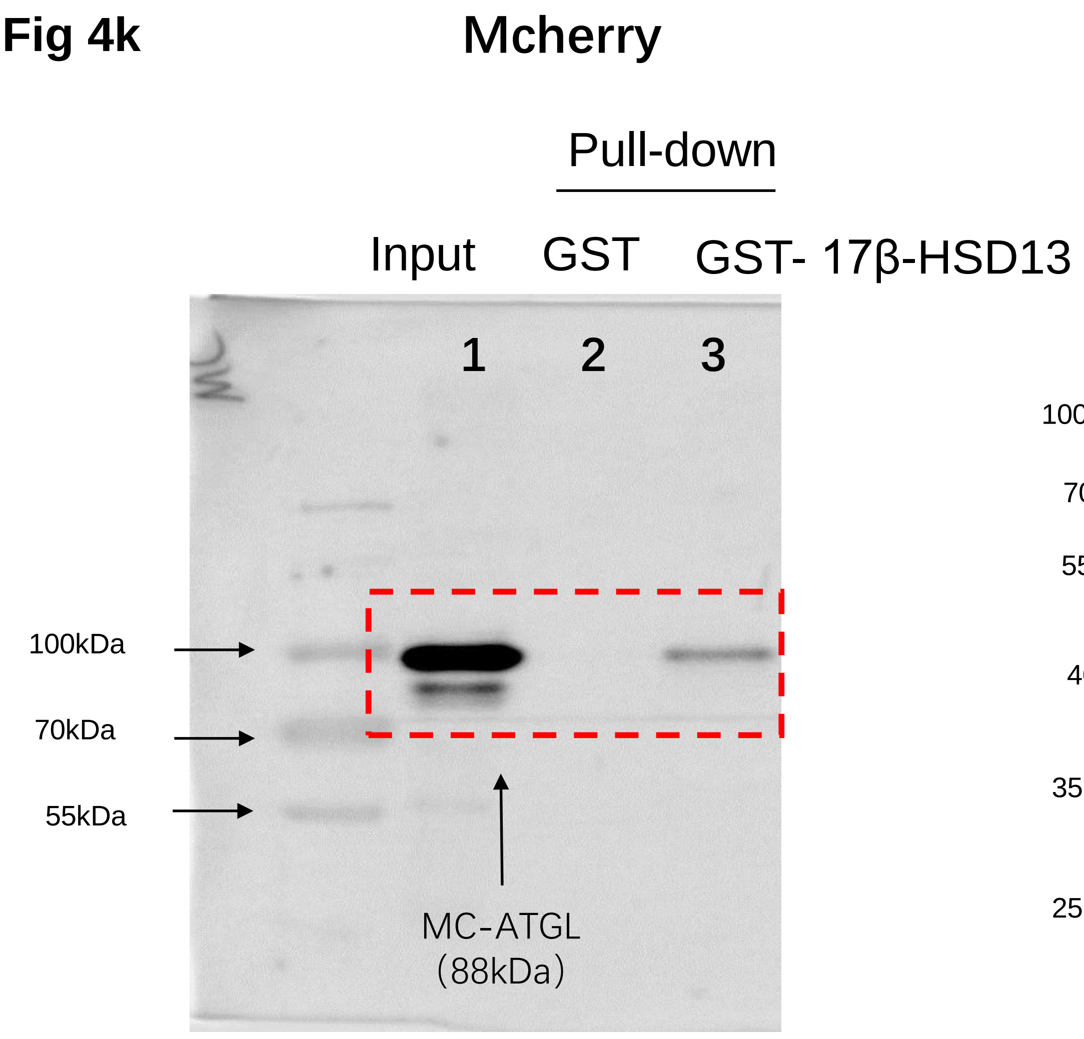
Mcherry
Pull-down
Input
GST
GST- 17β-HSD13
1
2
3
100kDa
70kDa
55kDa
MC-ATGL
(88kDa)

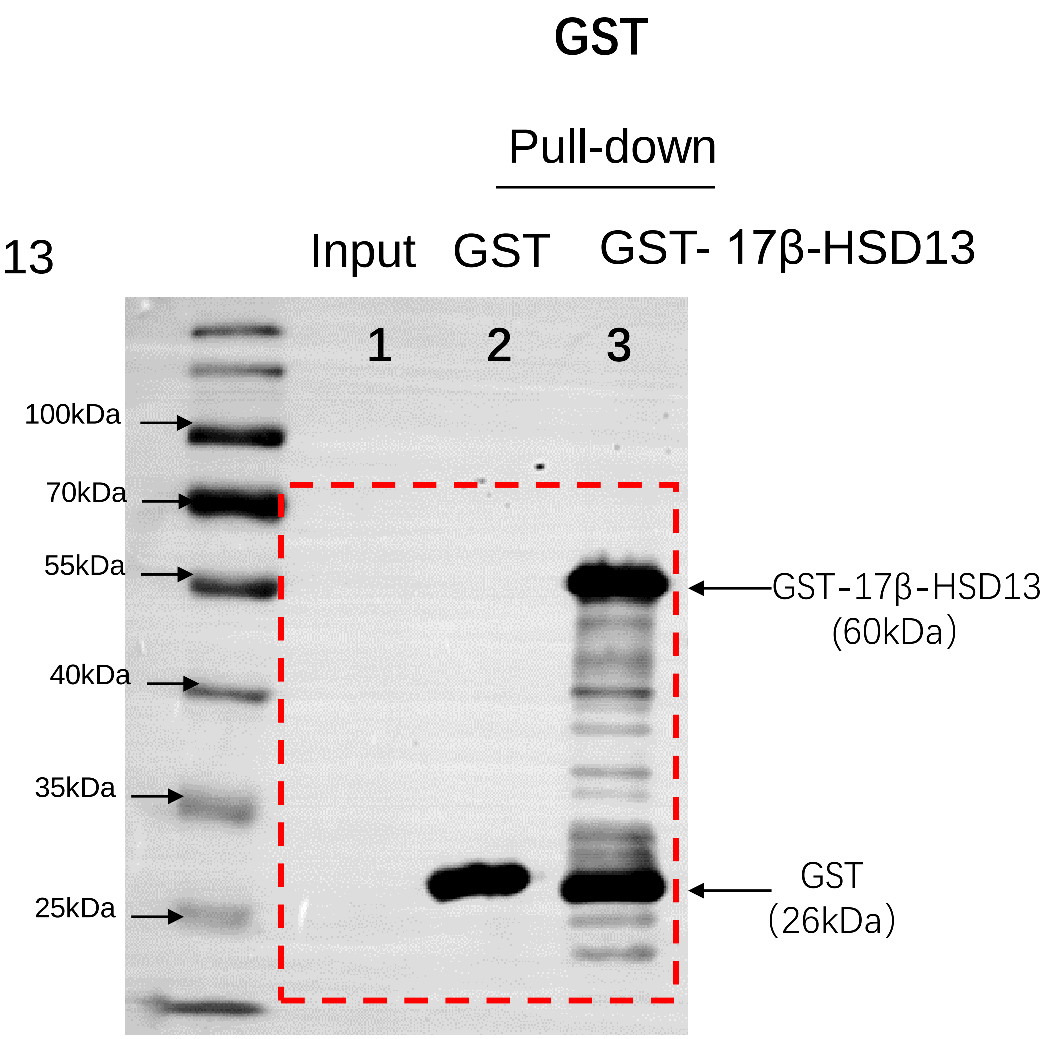
GST
Pull-down
Input
GST
GST- 17β-HSD13
1
2
3
100kDa
70kDa
55kDa
40kDa
35kDa
25kDa
GST-17β-HSD13
(60kDa)
GST
(26kDa)

Fig 5b

Input

GFP	+	-	-
GFP-17β-HSD13	-	+	-
GFP-17β-HSD13-S33A	-	-	+
MC-ATGL	+	+	+

IP

+	-	-
-	+	-
-	-	+
+	+	+

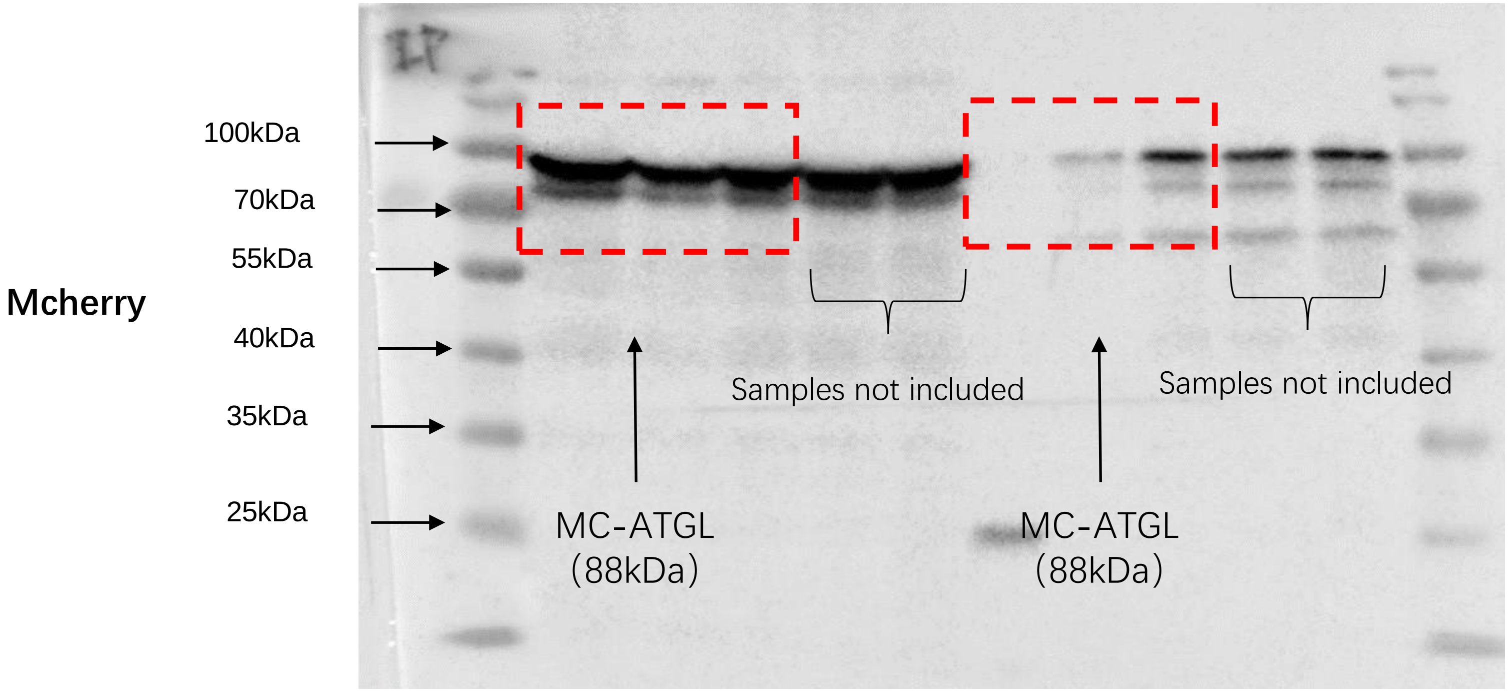

Fig 5g

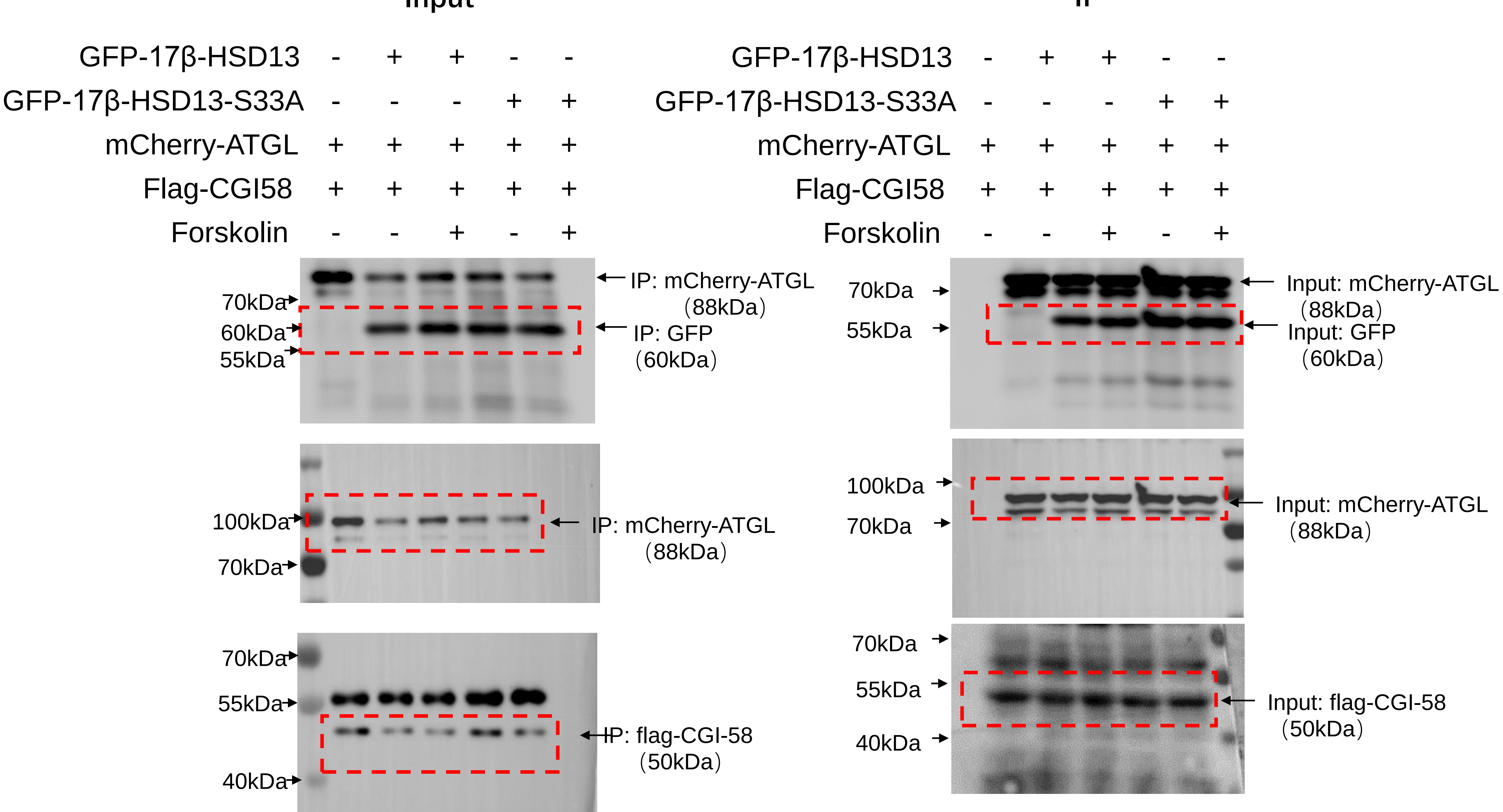
Input
IP
GFP-17β-HSD13 - + + - -
GFP-17β-HSD13-S33A - - - + +
mCherry-ATGL + + + + +
Flag-CGI58 + + + + +
Forskolin - - + - +
70kDa
60kDa
55kDa
IP: mCherry-ATGL (88kDa)
IP: GFP (60kDa)
100kDa
70kDa
IP: mCherry-ATGL (88kDa)
70kDa
55kDa
40kDa
IP: flag-CGI-58 (50kDa)
GFP-17β-HSD13 - + + - -
GFP-17β-HSD13-S33A - - - + +
mCherry-ATGL + + + + +
Flag-CGI58 + + + + +
Forskolin - - + - +
70kDa
55kDa
Input: mCherry-ATGL (88kDa)
Input: GFP (60kDa)
100kDa
70kDa
Input: mCherry-ATGL (88kDa)
70kDa
55kDa
40kDa
Input: flag-CGI-58 (50kDa)

Fig 8b

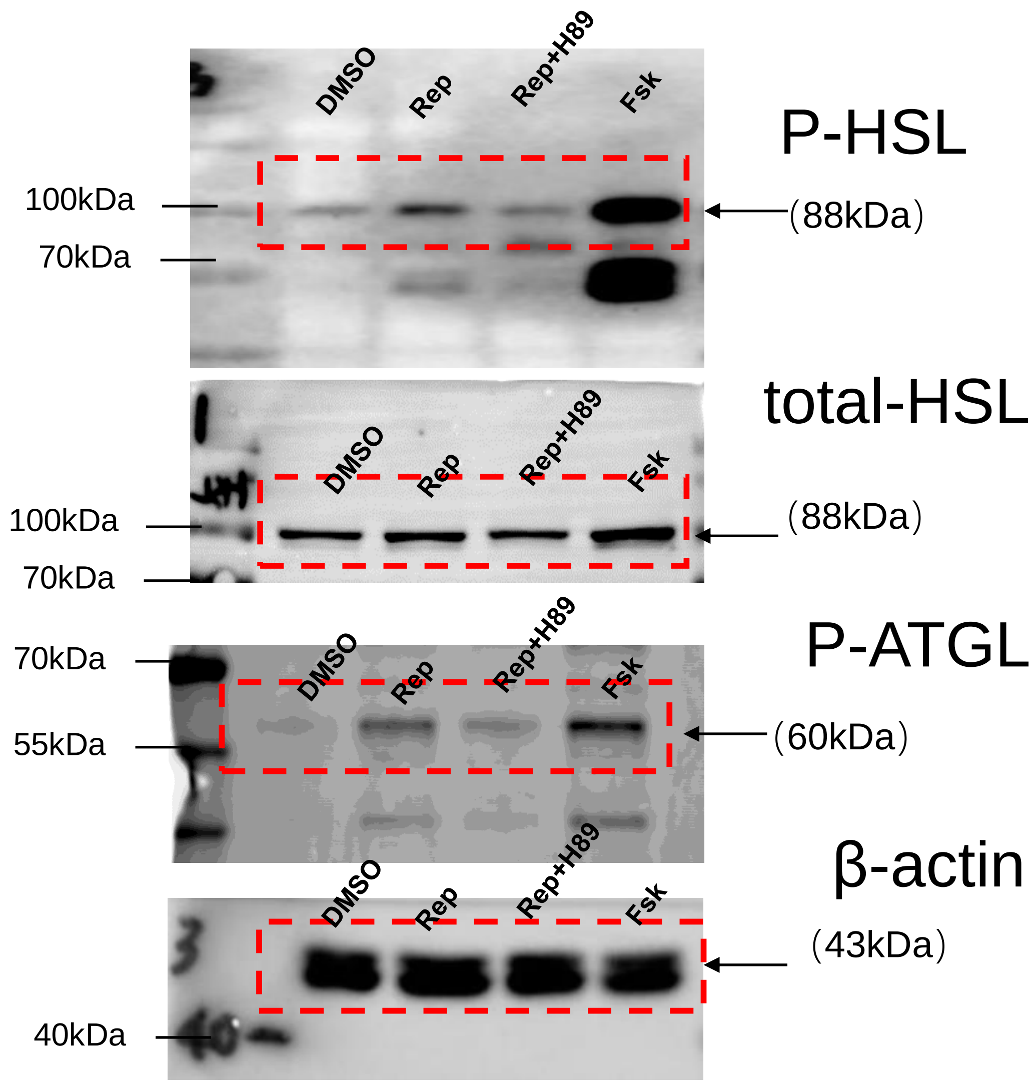
DMSO
Rep
Rep+H89
Fsk
P-HSL
100kDa
70kDa
(88kDa)
total-HSL
100kDa
70kDa
(88kDa)
P-ATGL
70kDa
55kDa
(60kDa)
β-actin
(43kDa)
40kDa

Fig 8c

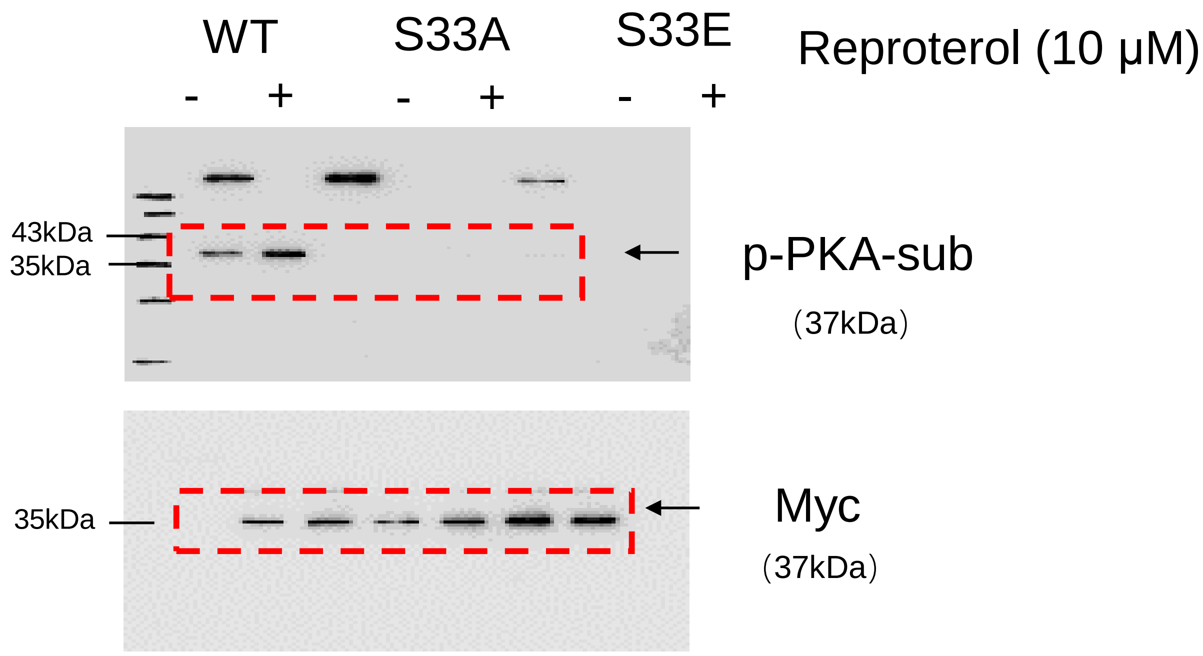
WT
S33A
S33E
Reproterol (10 μM)
-
+
-
+
-
+
43kDa
35kDa
p-PKA-sub
(37kDa)
35kDa
Myc
(37kDa)

Fig 8d

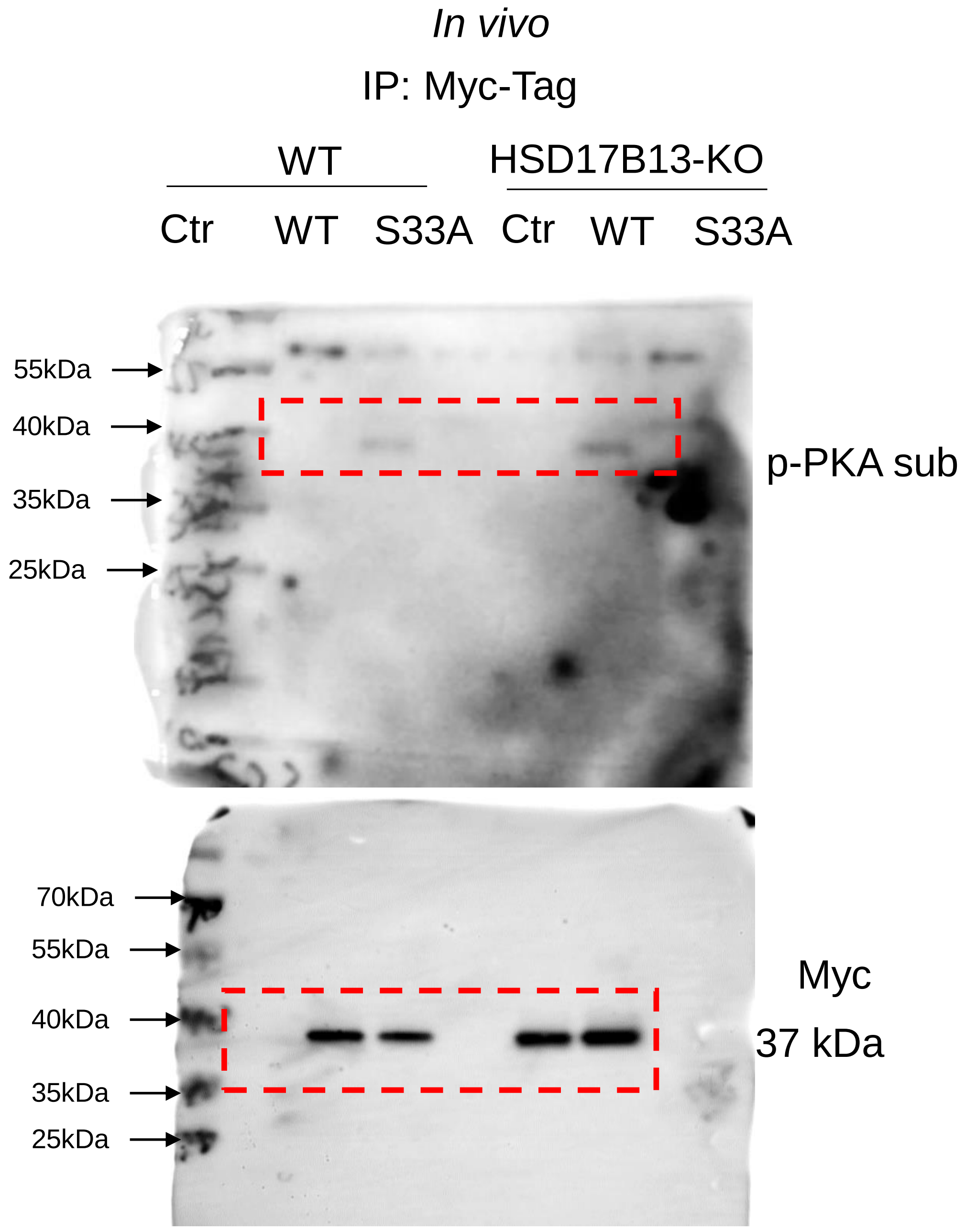

In vivo
IP: Myc-Tag
WT
HSD17B13-KO
Ctr
WT
S33A
Ctr
WT
S33A
55kDa
40kDa
35kDa
25kDa
p-PKA sub
70kDa
55kDa
40kDa
35kDa
25kDa
Myc
37 kDa

Fig 8p

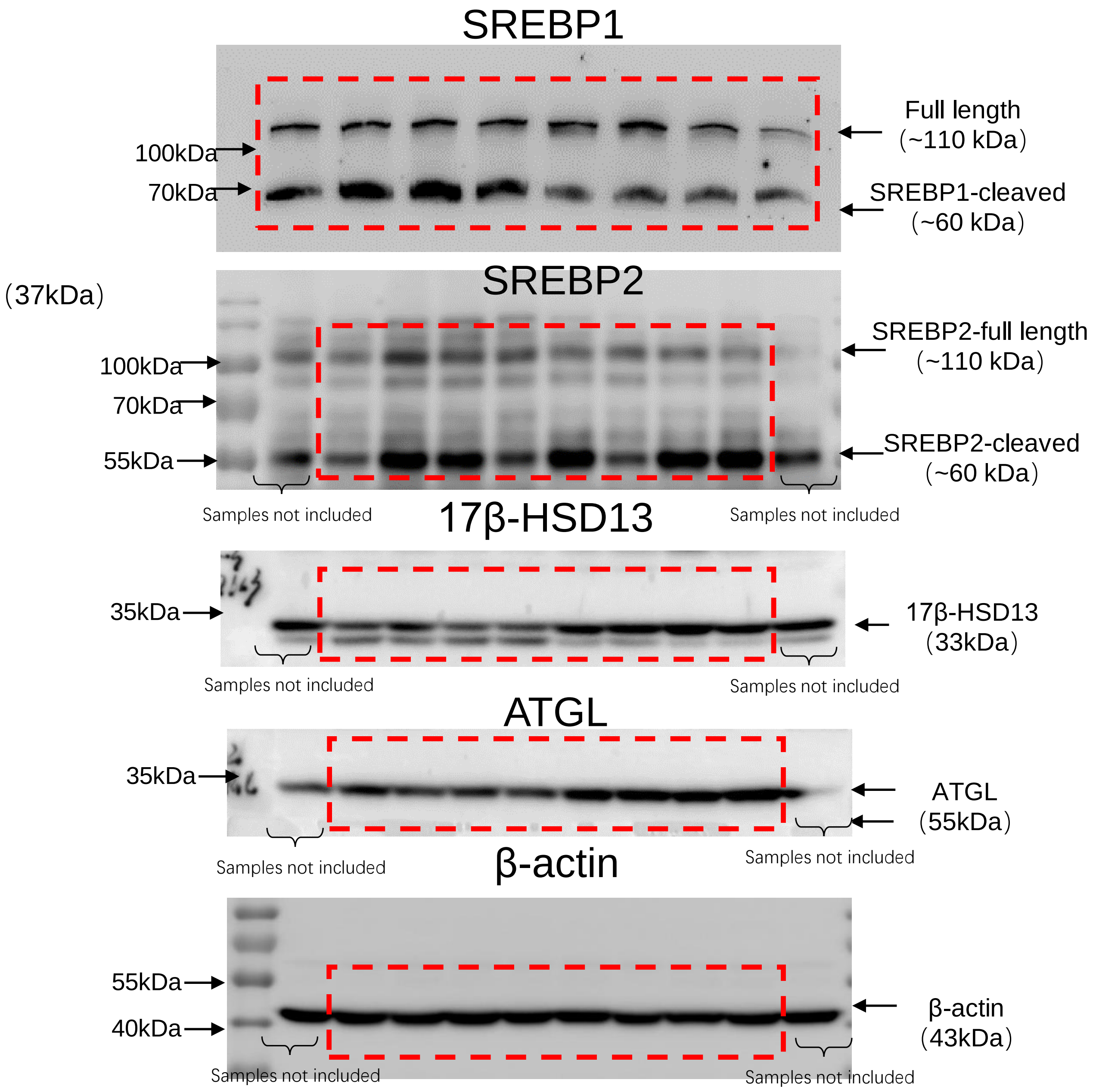

SREBP1
100kDa
70kDa
Full length
(~110 kDa)
SREBP1-cleaved
(~60 kDa)
(37kDa)
SREBP2
100kDa
70kDa
55kDa
SREBP2-full length
(~110 kDa)
SREBP2-cleaved
(~60 kDa)
Samples not included
Samples not included
17β-HSD13
35kDa
17β-HSD13
(33kDa)
Samples not included
Samples not included
ATGL
35kDa
ATGL
(55kDa)
Samples not included
Samples not included
β-actin
55kDa
40kDa
β-actin
(43kDa)
Samples not included
Samples not included
